# Integrative genomics analysis of various omics data and networks identify risk genes and variants vulnerable to childhood-onset asthma

**DOI:** 10.1186/s12920-020-00768-z

**Published:** 2020-08-31

**Authors:** Xiuqing Ma, Peilan Wang, Guobing Xu, Fang Yu, Yunlong Ma

**Affiliations:** 1 Department of Pulmonary & Critical Care Medicine, Chinese PLA General Hospital, Beijing, 100853 China; 2 Outpatient Department, Chinese PLA General Hospital, Beijing, 100853 China; 3 Department of Cardiovascular Medicine, Zhongxiang People’s Hospital, Zhongxiang, 431900 Hubei Province China; 4 Department of Pediatrics, Chinese PLA General Hospital, Beijing, 100853 China; 5 Institute of Biomedical Big Data, School of Biomedical Engineering, School of Ophthalmology & Optometry and Eye Hospital, Wenzhou Medical University, Wenzhou, 325027 P. R. China; 6 State Key Laboratory for Diagnosis and Treatment of Infectious Diseases, The First Affiliated Hospital, Collaborative Innovation Center for Diagnosis and Treatment of Infectious Diseases, Zhejiang University School of Medicine, Hangzhou, China

**Keywords:** Genetic variants, GWAS, Risk genes, Gene expression, Asthma

## Abstract

**Background:**

Childhood-onset asthma is highly affected by genetic components. In recent years, many genome-wide association studies (GWAS) have reported a large group of genetic variants and susceptible genes associated with asthma-related phenotypes including childhood-onset asthma. However, the regulatory mechanisms of these genetic variants for childhood-onset asthma susceptibility remain largely unknown.

**Methods:**

In the current investigation, we conducted a two-stage designed Sherlock-based integrative genomics analysis to explore the cis- and/or trans-regulatory effects of genome-wide SNPs on gene expression as well as childhood-onset asthma risk through incorporating a large-scale GWAS data (*N* = 314,633) and two independent expression quantitative trait loci (eQTL) datasets (*N* = 1890). Furthermore, we applied various bioinformatics analyses, including MAGMA gene-based analysis, pathway enrichment analysis, drug/disease-based enrichment analysis, computer-based permutation analysis, PPI network analysis, gene co-expression analysis and differential gene expression analysis, to prioritize susceptible genes associated with childhood-onset asthma.

**Results:**

Based on comprehensive genomics analyses, we found 31 genes with multiple eSNPs to be convincing candidates for childhood-onset asthma risk; such as, *PSMB9* (cis-rs4148882 and cis-rs2071534) and *TAP2* (cis-rs9267798, cis-rs4148882, cis-rs241456, and trans-10,447,456). These 31 genes were functionally interacted with each other in our PPI network analysis. Our pathway enrichment analysis showed that numerous KEGG pathways including antigen processing and presentation, type I diabetes mellitus, and asthma were significantly enriched to involve in childhood-onset asthma risk. The co-expression patterns among 31 genes were remarkably altered according to asthma status, and 25 of 31 genes (25/31 = 80.65%) showed significantly or suggestively differential expression between asthma group and control group.

**Conclusions:**

We provide strong evidence to highlight 31 candidate genes for childhood-onset asthma risk, and offer a new insight into the genetic pathogenesis of childhood-onset asthma.

## Background

Asthma is a complex and chronic respiratory disease that is diagnosed by evaluating the presence of reversible airflow obstruction and clinical symptoms, including cough, wheeze, and episodic shortness of breath [1]. Around 334 million individuals worldwide suffer from asthma, affecting 14% of children in the whole world [2]. Childhood asthma is a known risk factor for decreased lung function and chronic obstructive pulmonary disease (COPD) in adults [3–5]. Childhood asthma is significantly affected by genetic determinants [6–8]. The heritability of childhood asthma is estimated to range from 68 to 92% [8–10]. Thereby, there have been considerable interests in characterizing the genetic components that exert crucial effects on the aetiology of childhood-onset asthma, which may promote the development of better asthma control and effective treatments.

In the past decade, a plenty of genetic studies including candidate gene-based association studies, positional cloning studies, and genome-wide association studies (GWAS) have been performed to investigate the genetic architecture of both childhood-onset and adult-onset asthma [11]. Especially in recent years, with the advance of microarray and sequencing technology, GWAS as an effective and powerful method has been extensively employed. Since the first asthma-relevant GWAS was reported in the year of 2007 [12], subsequent many GWAS studies [7, 13–21] have been conducted and a growing number of genetic loci have been identified to be associated with asthma-related phenotypes including age of asthma onset and severe to moderate asthma. Very recently, two GWAS studies [15, 16] using data from the UK Biobank database were performed to identify shared and distinct genetic risk loci for adult-onset asthma and childhood-onset asthma. The genetic correlation between adult-onset asthma and childhood-onset asthma was estimated to be 0.67 [16]. Pividori and coworkers [15] identified 61 independent genetic loci significantly associated with asthma. Among these independent loci, 23 were specific to childhood-onset asthma, one was specific to adult-onset asthma, and 37 were common between both traits. Since GWAS generally concentrates on examining the genetic associations of individual SNPs and only reports top-ranked disease-associated SNPs with significantly statistical evidence for disease risk, many common variants with small marginal effects but rather act jointly or interact with together were ignored due to stringent multiple correction of GWAS [22]. Although GWASs have been successful in detecting newly genetic variants, the genetic components detected hitherto elucidate only a small part of asthma susceptibility.

To complement the typical GWAS analysis for individual SNPs, more integrative genomics studies by integrating GWAS data with other layers of omics data are warranted to identify sets of functional genes for childhood-onset asthma risk. Previous studies [23, 24] have showed that the vast majority of GWAS-identified SNPs are mapped within non-coding genomic regions. Thus, these SNPs predisposed to have *cis*- and/or *trans*-regulatory roles in modulating the expression level of a specific gene [25]. For example, Moffatt and colleagues [12] have demonstrated that genetic variants strongly and significantly associated in *cis* with transcript levels of *ORMDL3* are determinants of susceptibility to childhood asthma. Accumulating genomics studies have reported to explore whether GWAS-nominated genes whose differential changes of transcription levels are correlated with complex diseases due to pleiotropy [26–30]. Recently, He and coworkers [26] introduced a Sherlock integrative genomics analysis based on a Bayesian-based inference method to integrate genetic data from GWAS with existing eQTL data. Comparison of typical GWAS approach that generally abandon a large number of common genetic variants with moderate-to-small effects, Sherlock analysis is an effective and powerful tool for utilizing these abandoned common variants in GWAS. By using this tool, many novel risk genes, which are difficult to be identified by any single typical GWAS, were prioritized to involve in the pathogenesis of numerous complex diseases, including schizophrenia [31], major depressive disorders [32, 33], and gout disease [34].

In current study, the primary goal is designed to identify whether GWAS-nominated SNPs are correlated with both gene expression and childhood-onset asthma risk, and highlight novel susceptible genes. In the discovery stage, we conducted a Sherlock-based integrative genomics analysis by integrating a large-scale GWAS summary dataset with an eQTL dataset to identify expression-associated SNPs and risk genes for childhood-onset asthma. To validate the findings of the discovery stage, we re-performed the Sherlock analysis in an independent eQTL dataset. Furthermore, we employed systematical bioinformatics-based analyses based on multi-layers of evidence to highlight the underlie roles of novel identified genes in the pathogenesis of childhood-onset asthma.

## Methods

### GWAS datasets used in the current investigation

#### Dataset #1 GWAS summary dataset on childhood-onset asthma

In the present study, we employed a large-scale GWAS summary dataset on childhood-onset asthma [16] for identifying susceptibility SNPs and genes. For this GWAS on childhood-onset asthma, there were 13,962 affected individuals and 300,671 controls in the UK Biobank study used for examining the genome-wide association hits. Individuals in the control group did not suffer from any allergic disease, including asthma, eczema, hay fever, or other allergies. To select these non-allergic controls, the question of “Has a doctor ever told you that you have had any of the conditions below?”, which included “hay fever or allergic rhinitis” and “asthma” as possible answers, was used in the UK Biobank data fields (ID: 6152, 20,002, 41,202, 42,104, and 22,127). A number of 9,020,834 directly genotyped or imputed and quality passed autosomal variants were included in the linear mixed model. Discrete covariates included age, gender, and an indicator of the genotyping used. The informed consent was obtained from all participants, and the ethical approval was obtained from the Human Ethics Committee of the QIMR Berghofer Medical Research Institute, the ALSPAC Ethics and Law Committee, and the local research ethics committees.

### eQTL datasets used in the current investigation

#### Dataset #2 eQTL data for discovery

We first employed the monocyte eQTL dataset reported by Zeller et al. [35] as the discovery eQTL dataset to create the links between SNPs and gene expressions relevant to childhood asthma. For this dataset, 1490 unrelated participants with both DNA and RNA available were enrolled from a single-center cohort study of the Gutenberg Heart Study (GHS). Informed consent for each individual was signed. The Affymetrix Genome-wide Human SNP Array 6.0 (http://www.affymetrix.com) containing a total of 900,392 SNPs was employed to do the genome-wide genotyping. After utilizing a strict quality control of HWE, GCR, and MAF, a number of 675,350 SNPs remain for subsequent analysis. In addition, the Illumina HT-12 v3 BeadChip (http://www.Illumina.com) was used to conduct a genome-wide expression analysis for assessing the RNA expression levels of 37,804 genes. Among these genes, there were 22,305 genes obtaining prominent expression. Then, after omitting not well-characterized genes, a number of 12,808 well-characterized genes were chosen in the eQTL analysis.

#### Dataset #3 eQTL data for independent validation

We further used the eQTL dataset published by Dixon et al. [36] to carry out an independent Sherlock Bayesian analysis. With regard to this dataset, a total of 400 children were enrolled from families via a proband with asthma. Written informed consent were obtained for all included children. The ethical approval was obtained from the UK Multicentre Research Ethics Committee. Genome-wide genotyping were performed with the use of manufacturers’ protocols using the Human Hap300 Genotyping BeadChip (Illumina) and the Sentrix Human-1 Genotyping BeadChip in a BeadChip with full automation. In addition, the Affymetrix U133 Plus 2.0 GeneChip was applied to do the genome-wide expression analysis. Based on stringent inclusion criteria, these 400 asthmatic kids with both genotypes and gene expression data based on lymphoblastoid cells were used to generate an eQTL resource containing 54,675 transcripts (20,599 genes) and 408,273 genotyped SNPs.

### The inference method of Sherlock Bayesian integrative analysis

Here, we used the Sherlock analysis [26] by pooling the GWAS summary statistics of Ferreira et al. [16] with Zeller et al. eQTL data based on circulating monocyte samples [35] to reveal childhood-onset asthma-relevant genes. As for the procedure of the Sherlock algorithm, its first step is to utilize eQTL information to search expression-associated SNPs (named eSNPs). Then, the tool will test the association between eSNPs and childhood-onset asthma using GWAS summary dataset. At this step, the tool follows three judgmental scenarios: (1) A positive score would be recorded to an eSNP if this eSNP is significantly associated with childhood-onset asthma in the GWAS. (2) A negative score would be assigned to an eSNP if this eSNP is not significantly associated with childhood-onset asthma in the GWAS. (3) No score would be given if this SNP is not an eSNP. The scoring rubric of Sherlock algorithm increases the total gene score with using an aggregation of the scores of eSNPs. The logarithm of the Bayes Factor (LBF) is used as an important indicator to predict childhood-onset asthma-relevant risk genes. The *P* value is computed by the Sherlock analysis for each gene through simulation. The significance of each gene is adjusted by the Benjamini-Hochberg correction for multiple testing.

### Independent MAGMA gene-based enrichment analysis

As an independent technique for providing supportive evidence of risk genes identified by Sherlock integrative analysis, we employed a gene-based analysis with the use of Multi-marker Analysis of GenoMic Annotation (MAGMA; https://ctg.cncr.nl/software/magma) [37]. The SNP-based *P* values were extracted from childhood-onset asthma GWAS summary data as input for MAGMA gene-level analysis. For the MAGMA software, the multiple regression model was used to incorporate the linkage disequilibrium (LD) information among SNPs within a specific defined gene region and identify multi-variant combined effects. The SNP set of each gene was defined according to the location of the SNP whether located into the gene region or within extended +/− 20 kb downstream or upstream of the gene [38]. We used the data of 1000 Genome European panel as reference to evaluate the LD information between SNPs. The method of Bonferroni correction was employed for adjusting the *P* values.

### Pathway-based enrichment analysis

To annotate biological pathways and molecular functions of these identified genes by Sherlock Bayesian integrative analysis, we employed a pathway-based enrichment analysis with the use of the Database for Annotation, Visualization, and Integrated Discovery (DAVID; http://www.david.niaid.nih.gov) [39]. Based on the powerful pathway database of the Kyoto Encyclopedia of Genes and Genomes (KEGG) [40], we attempted to establish the biological link between risk genes and biochemical pathways. Further, we annotated the biological functions of identified risk genes using gene ontology (GO) database based on three functional categories: biological process (BP), cellular component (CC), and molecular function (MF). The hypergeometric test was employed to calculate the *P* value of each enrichment analysis. We used the Benjamini-Hochberg procedure to compute the false discovery rate (FDR) for multiple testing.

### Functional enrichment analysis based on multiple databases

Based on the identified gene list, we used the online database of WEB-based Gene SeT AnaLysis Toolkit (WebGestalt; http://www.webgestalt.org) [41] to perform a functional annotation enrichment analysis. WebGestalt software supports three well-documented and complementary methods for enrichment analysis, including network topology-based analysis, over-representation analysis, and gene set enrichment analysis. By using the over-representation method, we searched the drug-relevant gene sets of these identified genes from two drug databases of DrugBank [42] and GLAD4U [43], and enriched these genes into disease-related gene sets of DisGeNET [44] and GLAD4U [43] databases. All the enrichment analyses were based on the selected reference set of genome protein-coding genes. The number of genes in each category of gene set ranged from 5 to 2000. We also used the Benjamini-Hochberg FDR for multiple testing.

### Computer-based permutation analysis

As a previous study [45], we here conducted a computer-based permutation analysis (N_total_ = 100,000 times) to determine whether genes identified in the discovery stage were significantly overlapped with that identified from the replication stage and MAGMA analysis (N_i_ = N_1_, N_2_ overlapped gene number for each dataset) by comparison with genes selected from background. By randomly choosing the same number as the significant genes from whole genes as background of each dataset (N_background_ = 9821 ~ 19,233) for 10^5^ times, we counted the number of genes from random selection overlapped with genes identified in the discovery stage (n_j_ = n_1_, n_2_, n_3_…n_100,000_, overlapped gene number for each time random selection). Subsequently, we computed how many times of the number of genes for random selections were larger than the number of genes for real observation. Empirical *P* value = $\frac{\sum \left(\mathrm{nj}>\mathrm{Ni}\right)}{\mathrm{Ntotal}}$. The *P* value less than or equal to 0.05 considers to be significant.

### GeneMANIA-based PPI network analysis

With the use of GeneMANIA software (http://www.genemania.org) [46], we performed a protein-protein interaction (PPI) network-based analysis to identify the functional interaction patterns of these identified childhood-onset asthma-associated genes. Based on the information of inputted gene list, the GeneMANIA tool, a plug-in of Cytoscape platform, would predict genes with similar functions and establish interacted links by integrating current existing genomics and proteomics information, including shared protein domains, genetic interactions, co-expression associations, pathway links, physical interactions, co-localization, and predicted links.

### Identification of childhood-onset asthma-related genes expression profiles

We further downloaded two RNA expression datasets from NCBI GEO database (Accession Nos. GSE123750 and GSE103166) to replicate the functionality of these 31 identified genes. The first analyzed dataset of GSE123750 was based on blood RNA expression profiles that were collected samples from school-aged children who presented to mild-to-moderate asthma (*N* = 37) and severe asthma (*N* = 75) from the Unbiased Biomarkers for the Prediction of Respiratory Disease Outcomes (U-BIOPRED) consortium. Blood samples of this cross-sectional study were collected at baseline. Significance was examined by using Student’s T-test. *P* ≤ 0.05 was considered to be significant. The second used dataset of GSE103166 [47] was based on nasal swab specimens that were collected samples from children with the emergency department with an acute exacerbation of asthma or wheeze (*N* = 56) and age-matched controls (*N* = 31). For this dataset, a group of convalescent samples were also collected from children with follow-up at least 6 weeks after an acute exacerbation of asthma or wheeze (*N* = 19). One-way ANOVA analysis was used to calculate the significance among control, convalescent, and asthma groups. In addition, we performed a co-expression patterns analysis of these identified risk genes among different groups. We made the R script for this co-expression pattern analysis available in the public github website (https://github.com/mayunlong89/CoA/blob/master/co_expression_pattern.R).

## Results

### Integrative genomics analysis in the discovery stage

**Fig. 1:**
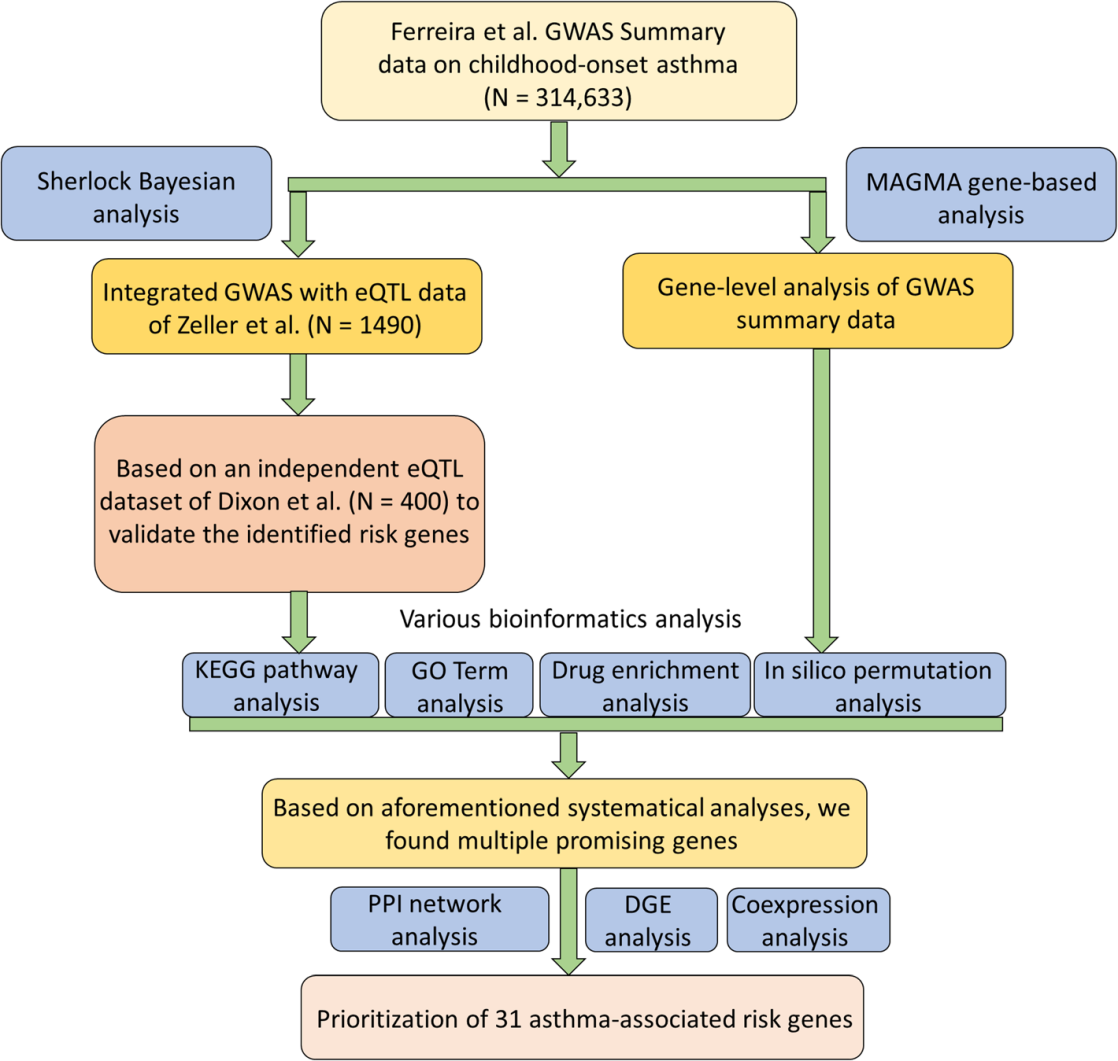
The workflow of current study for all the genomics analysis

In the discovery stage, we integrated GWAS summary statistics (*N* = 313,633) with eQTL data (*N* = 1490) to identify whether abnormal gene expression convey susceptibility to childhood-onset asthma by using the Sherlock Bayesian analysis. Figure 1 shows the workflow of the present integrative genomics study. At this stage, we found that a number of 560 genes were significantly associated with childhood-onset asthma risk after multiple corrections (FDR ≤ 0.05, Supplemental Table S1). For example, the top-ranked asthma-risk genes with eSNPs were identified to be significant: *HLA-DRD3* (FDR = 2.05 × 10^− 4^), *HLA-DQA1* (FDR = 2.05 × 10^− 4^), *HLA-DRB4* (FDR = 2.05 × 10^− 4^), *NOTCH4* (FDR = 2.05 × 10^− 4^), *PSMB9* (FDR = 2.05 × 10^− 4^), *PALZ* (FDR = 2.05 × 10^− 4^), *HLA-DRB5* (FDR = 2.05 × 10^− 4^), *HLA-DPB1* (FDR = 2.05 × 10^− 4^), and *HLA-DRB1* (FDR = 2.05 × 10^− 4^). Of note, 37 of 560 genes have been well-documented in the database of GWAS catalog (Supplemental Table S1).

### Gene-based enrichment analysis of GWAS on childhood-onset asthma

To validate the reliability of these identified childhood-onset asthma-relevant genes, we adopted an independent method of gene-level analysis with the use of MAGMA software. After Bonferroni correction for multiple testing of MAGMA-based analysis, we found 503 genes were associated with childhood-onset asthma (MAGMA-based *P* ≤ 2.56 × 10^− 6^). The top-ranked significant genes of MAGMA-based analysis were yielded by *HLA-DQA1* (MAGMA-based *P* = 3.38 × 10^− 64^), *LOC101928947* (MAGMA-based *P* = 8.07 × 10^− 61^), *ORMDL3* (MAGMA-based *P* = 4.55 × 10^− 58^), *HLA-DQB1* (MAGMA-based *P* = 5.70 × 10^− 57^), *GSDMA* (MAGMA-based *P* = 1.61 × 10^− 53^), *LRRC3C* (MAGMA-based *P* = 1.39 × 10^− 49^), *GSDMB* (MAGMA-based *P* = 7.60 × 10^− 48^), *ERBB2* (MAGMA-based *P* = 8.28 × 10^− 45^), and *IL18R1* (MAGMA-based *P* = 2.61 × 10^− 44^). The genes of *HLA-DQA1*, *NOTCH4*, *HLA-DRB5*, *HLA-DPB1*, *HLA-DRB1*, *ADORA1*, *TLR6*, and *IL18R1* have been previously reported to be associated with asthma risk (Supplemental Table S1). Among them, 83 genes were overlapped with Sherlock-identified genes in the discovery stage (Supplemental Table S2). Of note, 53 of 83 genes not documented in the GWAS Catalog database were newly identified to be associated with childhood-onset asthma in our analysis (Supplemental Table S2).

### Pathway-based enrichment analysis

**Fig. 2:**
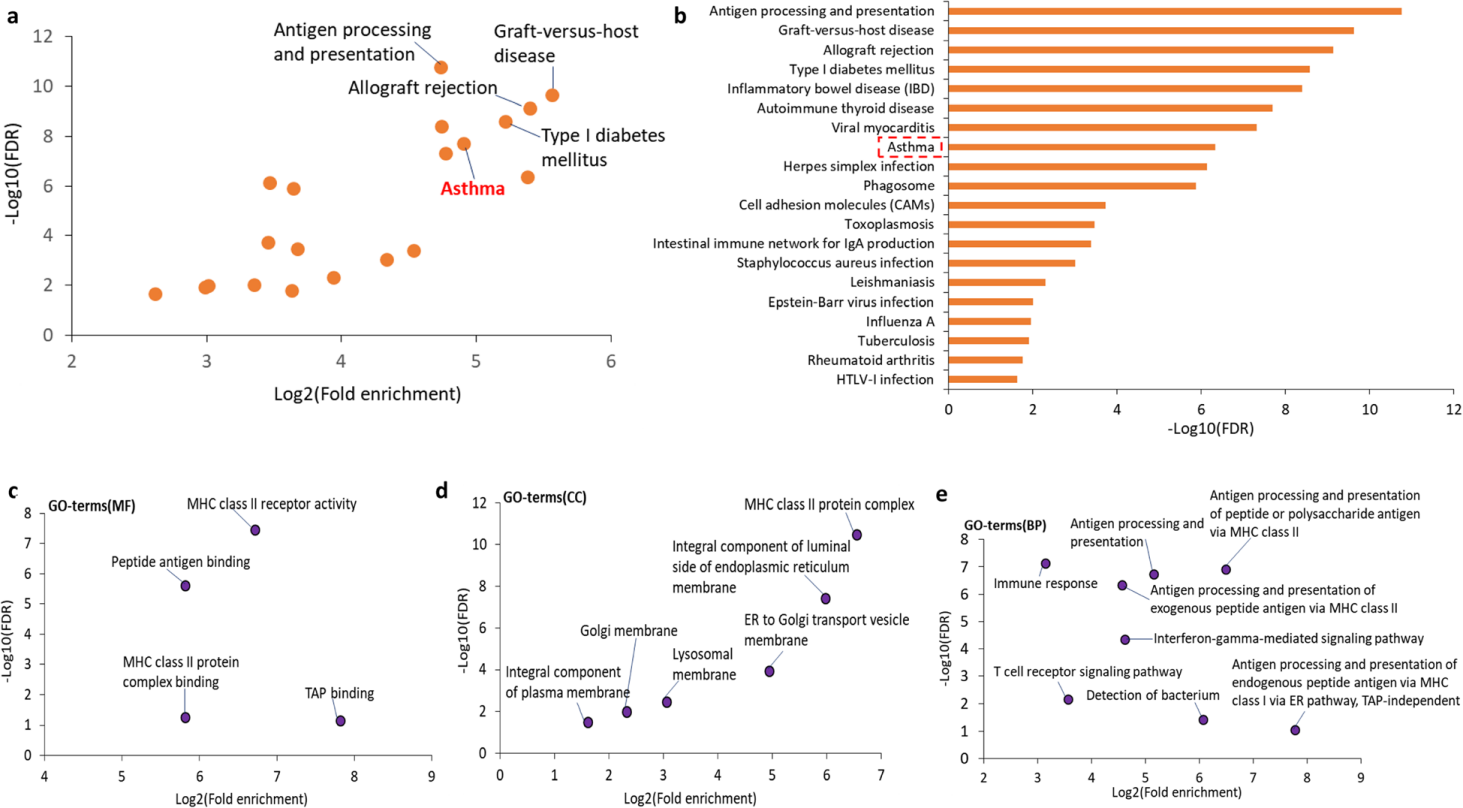
DAVID-based enrichment analysis of 83 childhood-onset asthma-related genes. **a** The scatter diagram shows the enrichment results of KEGG pathway analysis based on 83 genes. y axis represents the significant value of each enriched pathway based on the negative log10(FDR). x axis represents the enrichment value of each enriched pathway based on the log2(Fold enrichment). **b** KEGG pathway enrichment analysis for 83 identified genes with 20 molecular pathways. **c**-**e** The scatter diagram shows the enrichment results of three GO-terms enrichment analysis based on 83 genes. y axis represents the significant value of each enriched GO-term based on the negative log10(FDR). x axis represents the enrichment value of each enriched GO-term based on the log2(Fold enrichment). **c** for molecular function (MF); **d** for cellular component (CC); **e** for biological process (BP)

Furthermore, we performed a pathway-based enrichment analysis by using these identified 83 genes based on the KEGG pathway resource. We found a number of 20 KEGG biological pathways were significantly enriched by these genes (FDR < 0.05, Fig. 2a-b and Supplemental Table S3). The top-ranked significant pathways were Antigen processing and presentation (FDR = 1.77 × 10^− 11^), Graft-versus-host disease (FDR = 2.34 × 10^− 10^), and Allograft rejection (FDR = 7.41 × 10^− 10^). Interestingly, the pathway of asthma (FDR = 4.63 × 10^− 7^) showed a significant enrichment by these identified 83 genes (Fig. 2a-b). In addition, we also performed a gene ontology (GO) analysis based on the categories of molecular function (MF), cellular component (CC), and biological process (BP), separately. For the GO-term of MF, we detected MHC class II receptor activity (FDR = 3.84 × 10^− 8^) and peptide antigen binding (FDR = 2.51 × 10^− 6^) were significantly enriched (Fig. 2c and Supplemental Table S4). With respect to the term of CC, six GO-terms were remarkably enriched by these 83 identified genes (Fig. 2d and Supplemental Table S5); for example, MHC class II protein complex (FDR = 3.16 × 10^− 11^) and integral component of luminal side of endoplasmic reticulum membrane (FDR = 3.81 × 10^− 8^). As for the term of BP, there were seven GO-terms significantly overrepresented by these 83 genes (Fig. 2e and Supplemental Table S6); for example, immune response (FDR = 7.46 × 10^− 8^) and antigen processing and presentation of exogenous peptide antigen via MHC class II (FDR = 4.67 × 10^− 7^).

### Functional enrichment analysis of disease- and drug-relevant gene sets

**Fig. 3:**
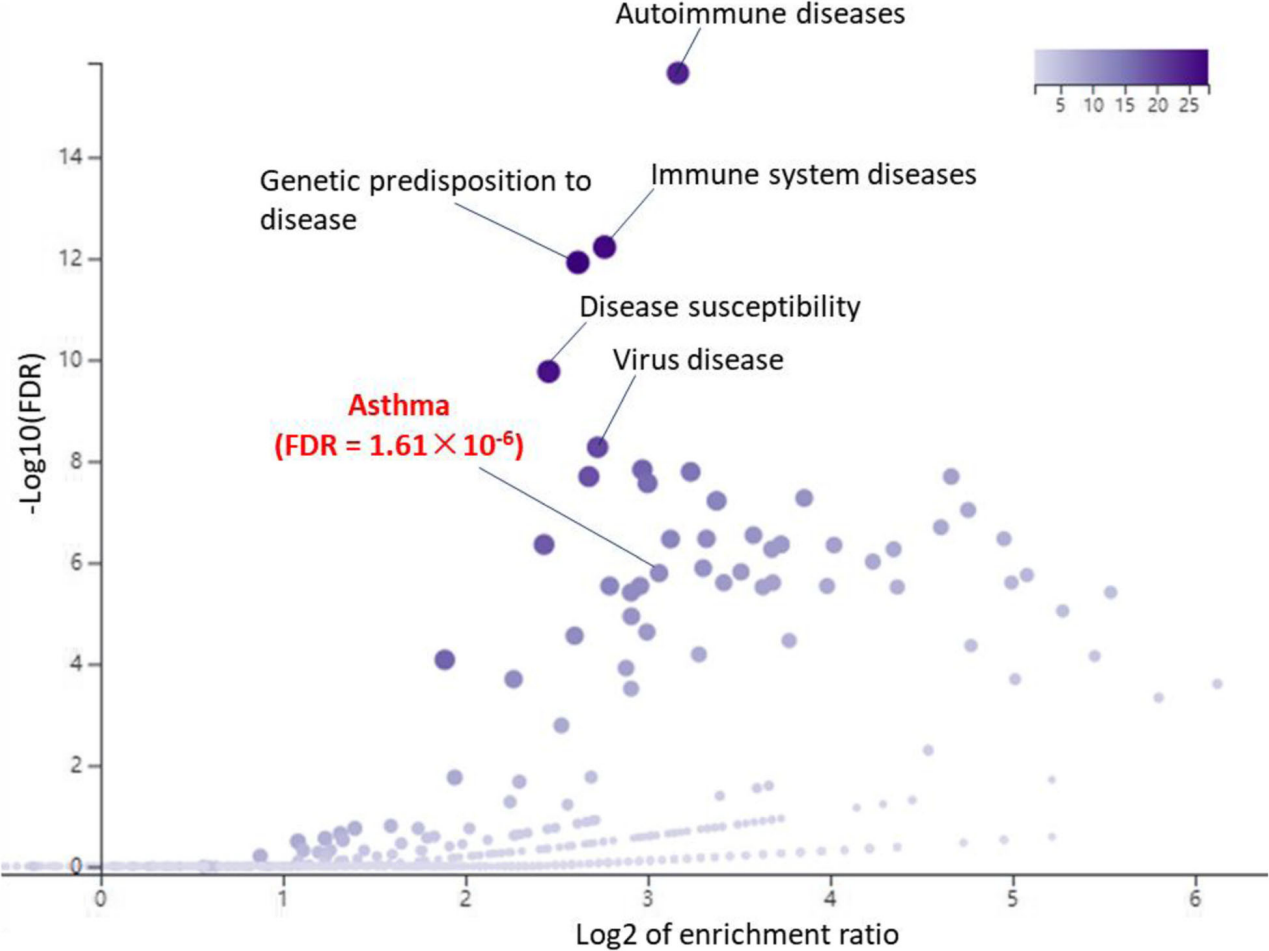
Disease-based enrichment analysis of 83 childhood-onset asthma-related genes. The scatter diagram was plotted based on the database of GLAD4U by using the WebGestalt software. y axis represents the significant value of each enriched pathway based on the negative log10(FDR). x axis represents the enrichment value of each enriched pathway based on the log2(Enrichment ratio). The intensity of the color stands for the negative log10(FDR) of each enriched pathway, as indicated on the bar on the right of scatter plot. Each dot represents a given pathway, and the size of dot showed the gene set size of each enriched pathway

In addition, we performed a functional enrichment analysis of disease-related gene sets based on two databases of GLAD4U and DisGeNET. We found that 69 significant gene sets relevant to different diseases were enriched by these identified 83 genes (Fig. 3, Supplemental Fig. S1 and Supplemental Tables S7-S8). For example, the top-ranked enriched diseases were Autoimmune diseases (FDR < 1.0 × 10^− 16^), Immune system diseases (FDR = 6.0 × 10^− 16^), Asthma (FDR = 1.61 × 10^− 6^), and Drug allergy (FDR = 9.02 × 10^− 3^). Subsequently, we also conducted a functional enrichment analysis of drug-related gene sets based on two databases of GeneBank and GLAD4U. A number of 29 significant gene sets relevant to different drugs were significantly overrepresented by these 83 genes (Supplemental Figs. S2-S3 and Supplemental Tables S9-S10).

### Integrative genomics analysis based on an independent dataset in the replication stage

**Fig. 4:**
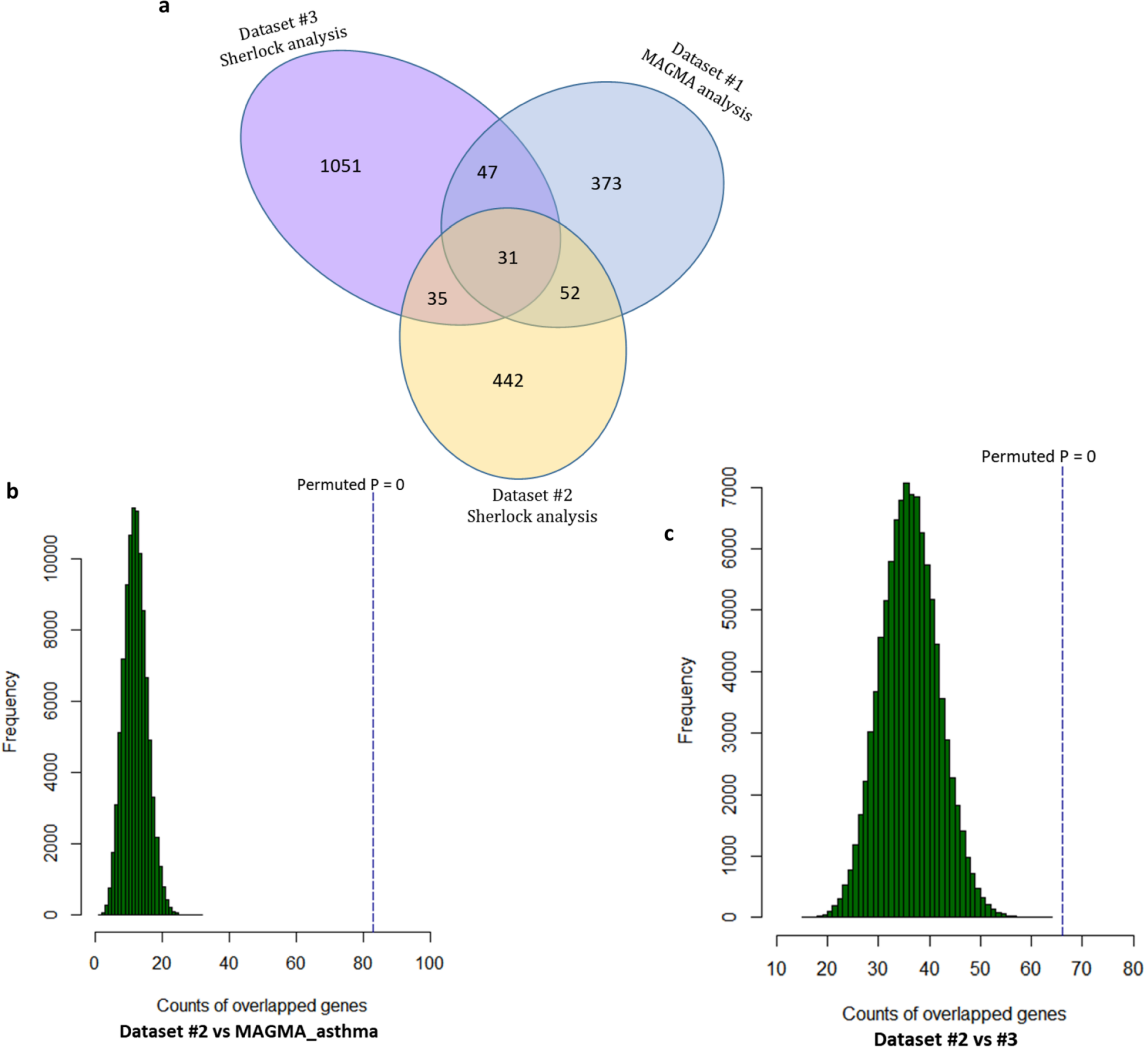
Consistent evidence of childhood-onset asthma-relevant genes based on independent datasets and techniques. **a** Venn diagram of three identified childhood-onset asthma-relevant gene sets: Dataset #1 based on MAGMA analysis of GWAS summary data (*P* value of each gene with Bonferroni correction), Dataset #2 based on Sherlock integrative analysis of integrating GWAS and Zeller et al. eQTL data (P value of each gene with FDR correction), and Dataset #3 based on Sherlock analysis of integrating GWAS and Dixon et al. eQTL data (raw P value of each gene was applied). **b** Computer-based permutation analysis of 10^5^ times for the comparison of genes with adjusted *P*-values from Dataset #2 with that from MAGMA analysis. **c** Computer-based permutation analysis of 10^5^ times for the comparison of genes with adjusted P-values from Dataset #2 with that from Dataset #3

To further confirm the validity of above identified genes, we re-conducted the Sherlock Bayesian analysis with using the same parameter settings based on an independent eQTL dataset (Dataset #3). We found a number of 1164 significant or suggestive asthma-associated genes from the dataset #3 (Sherlock-based *P* < 0.05; Fig. 4a). Compared identified genes from Dataset #2 in the discovery stage with those from Datasets #3 and #1, we found that there existed a high number of overlapped genes across three identified gene sets (Fig. 4a and Supplemental Fig. S4a). Based on the 10^5^ times permutation analysis, we found the number of genes in Dataset #2 overlapped with that in Datasets #1 (Permuted *P* = 0, Fig. 4b and Supplemental Fig. S4b) and #3 (Permuted *P* = 0, Fig. 4c and Supplemental Fig. S4c) were very significantly higher than genes randomly selected from background genes.

Based on independent biological and technical replications, we prioritized a number of 31 childhood-onset asthma-risk genes with multiple significant eSNPs across all analyses (Fig. 4a and Table 1). A number of 13 genes have been documented to implicate in asthma risk in the GWAS catalog and previous studies; for example, *HLA-DQA1*, *HLA-DRB5*, *HLA-DRB1*, *TLR6*, and *MPHOSPH9*. Interestingly, there were 18 genes newly identified to be associated with childhood-onset asthma; e.g., *PSMB9*, *TAP2*, *PMM1*, and *ACTR1A*. Except for *PSMD3*, none of these 31 genes obtained any significant or suggestive association signals from MAGMA analysis of GWAS data on Null phenotype (Table 1). For each identified gene, one or more eSNPs were identified to be significantly associated with expression level of the gene and childhood-onset asthma risk (Supplemental Table S11). For example, two cis-eSNPs of rs4148882 (*P*_eQTL_ = 2.32 × 10^− 19^ and *P*_GWAS_ = 5.80 × 10^− 9^) and rs2071534 (*P*_eQTL_ = 1.30 × 10^− 8^ and *P*_GWAS_ = 8.49 × 10^− 6^) have regulatory effects on *PSMB9* gene. With regard to *TAP2* gene, one trans-regulatory eSNPs of rs10447456 (*P*_eQTL_ = 1.23 × 10^− 6^ and *P*_GWAS_ = 5.79 × 10^− 3^) and three cis-regulatory eSNPs of rs9267798 (*P*_eQTL_ = 6.68 × 10^− 6^ and *P*_GWAS_ = 8.72 × 10^− 4^), rs4148882 (*P*_eQTL_ = 3.71 × 10^− 11^ and *P*_GWAS_ = 5.80 × 10^− 9^), and rs241456 (*P*_eQTL_ = 1.40 × 10^− 16^ and *P*_GWAS_ = 2.16 × 10^− 27^) were detected.

**Table 1:** The results of 31 childhood-onset asthma-relevant risk genes based on current integrative genomics analysis

Gene name	Risk eSNPs	LBF	Sherlock-based P-value (Dataset #3)	FDR value (Dataset #3)	MAGMA-based P-value (Dataset #1)	MAGMA-based corrected P-value (Dataset #1)	Sherlock-based P-value (Dataset #4)	GWAS Catalog
*HLA-DQA1*	rs17426593, rs1150753, rs660895	12.91	7.87 × 10^− 7^	2.05 × 10^− 4^	1.76 × 10^− 68^	3.38 × 10^− 64^	3.79 × 10^− 4^	Reported genes
*PSMB9*	rs2071534, rs4148882	12.17	7.87 × 10^− 7^	2.05 × 10^− 4^	1.08 × 10^− 9^	2.09 × 10^− 5^	2.43 × 10^− 4^	Novel genes
*HLA-DRB5*	rs9272346, rs2071295	11.52	7.87 × 10^− 7^	2.05 × 10^− 4^	9.80 × 10^−17^	1.88 × 10^− 12^	3.19 × 10^− 5^	Reported genes
*HLA-DPB1*	rs429916, rs2855430	11.26	7.87 × 10^− 7^	2.05 × 10^− 4^	5.12 × 10^− 11^	9.85 × 10^− 7^	1.52 × 10^− 6^	Novel genes
*HLA-DRB1*	rs9272346, rs660895	10.91	7.87 × 10^− 7^	2.05 × 10^− 4^	1.47 × 10^− 24^	2.83 × 10^− 20^	7.59 × 10^− 6^	Reported genes
*TAP2*	rs4148882, rs241456	8.70	7.87 × 10^−7^	2.05 × 10^− 4^	1.07 × 10^− 33^	2.06 × 10^− 29^	1.52 × 10^− 6^	Novel genes
*TLR6*	rs5743592, rs5743595	7.38	7.87 × 10^−7^	2.05 × 10^− 4^	1.83 × 10^− 26^	3.51 × 10^− 22^	1.52 × 10^− 6^	Reported genes
*PMM1*	rs203319, rs132774	7.36	7.87 × 10^−7^	2.05 × 10^− 4^	5.24 × 10^−9^	1.01 × 10^− 4^	1.52 × 10^− 6^	Novel genes
*MPHOSPH9*	rs7299943, rs1716160	7.20	7.87 × 10^−7^	2.05 × 10^− 4^	3.51 × 10^−9^	6.75 × 10^− 5^	1.67 × 10^− 5^	Reported genes
*PSMD3*	rs12311409, rs8077456	6.83	1.57 × 10^− 6^	2.05 × 10^−4^	6.49 × 10^− 42^	1.25 × 10^− 37^	3.27 × 10^−2^	Novel genes
*HLA-DOB*	rs2071474, rs2621332	6.67	1.57 × 10^− 6^	2.05 × 10^−4^	6.25 × 10^− 32^	1.20 × 10^−27^	1.52 × 10^− 6^	Novel genes
*ME2*	rs1810129, rs584357	6.60	1.57 × 10^−6^	2.05 × 10^−4^	1.26 × 10^−9^	2.42 × 10^− 5^	1.88 × 10^− 4^	Novel genes
*ACTR1A*	rs2281879, rs10883723	6.55	1.57 × 10^−6^	2.05 × 10^−4^	3.42 × 10^−9^	6.58 × 10^−5^	3.03 × 10^− 6^	Novel genes
*DEXI*	rs12935657, rs741175	6.42	1.57 × 10^−6^	2.05 × 10^−4^	2.32 × 10^−13^	4.45 × 10^−9^	1.52 × 10^− 6^	Novel genes
*NSMCE1*	rs3024530, rs6498001	6.22	1.57 × 10^−6^	2.05 × 10^−4^	6.37 × 10^−12^	1.22 × 10^−7^	6.37 × 10^−5^	Novel genes
*JAZF1*	rs2189966, rs1635852	6.18	1.57 × 10^−6^	2.05 × 10^−4^	2.67 × 10^−9^	5.13 × 10^−5^	5.61 × 10^− 5^	Reported genes
*TDRKH*	rs868867, rs1054475	6.18	1.57 × 10^−6^	2.05 × 10^−4^	5.17 × 10^− 11^	9.95 × 10^−7^	3.03 × 10^− 6^	Reported genes
*SMARCE1*	rs1358174, rs757411	6.06	1.57 × 10^−6^	2.05 × 10^−4^	4.18 × 10^− 10^	8.05 × 10^− 6^	7.59 × 10^− 6^	Reported genes
*AHI1*	rs2757649, rs11154801	5.89	1.57 × 10^−6^	2.05 × 10^−4^	2.10 × 10^−6^	4.03 × 10^−2^	1.84 × 10^− 4^	Novel genes
*SLC15A2*	rs9826473, rs1806656	5.77	1.57 × 10^−6^	2.05 × 10^−4^	3.32 × 10^−9^	6.39 × 10^−5^	3.03 × 10^− 6^	Novel genes
*RAD50*	rs2069812	5.60	1.57 × 10^−6^	2.05 × 10^−4^	8.32 × 10^−25^	1.60 × 10^− 20^	6.22 × 10^−5^	Reported genes
*HLA-B*	rs3130564, rs3130564	5.50	1.57 × 10^−6^	2.05 × 10^−4^	5.61 × 10^−29^	1.08 × 10^−24^	2.88 × 10^− 4^	Reported genes
*LST1*	rs2071593, rs2855812, rs1861856	5.14	6.30 × 10^−6^	7.39 × 10^−4^	1.31 × 10^−15^	2.52 × 10^−11^	1.52 × 10^− 6^	Novel genes
*POLI*	rs2276182, rs3730783	5.12	6.30 × 10^−6^	7.39 × 10^−4^	9.06 × 10^−12^	1.74 × 10^−7^	1.52 × 10^− 6^	Reported genes
*HSPA1A*	rs2276133, rs4143780	4.80	7.87 × 10^−6^	8.10 × 10^−4^	4.66 × 10^−13^	8.97 × 10^−9^	3.75 × 10^−2^	Novel genes
*SLC22A5*	rs17622656, rs11950562	4.54	1.42 × 10^−5^	1.33 × 10^−3^	1.77 × 10^−7^	3.41 × 10^−3^	3.03 × 10^−6^	Reported genes
*CTSW*	rs659824, rs494003	4.15	2.52 × 10^−5^	2.06 × 10^−3^	1.08 × 10^−7^	2.08 × 10^−4^	3.94 × 10^− 5^	Novel genes
*NDFIP1*	rs6498142, rs449454	3.93	4.25 × 10^−5^	2.99 × 10^−3^	4.41 × 10^−13^	8.49 × 10^−9^	3.03 × 10^−6^	Reported genes
*HLA-C*	rs1063646, rs3130564	3.71	5.83 × 10^−5^	3.73 × 10^−3^	1.32 × 10^−15^	2.54 × 10^−11^	1.52 × 10^− 5^	Novel genes
*CD52*	rs6703878, rs1071849, rs3780032	1.42	7.75 × 10^−4^	2.19 × 10^−2^	2.04 × 10^−6^	3.93 × 10^− 2^	9.21 × 10^− 4^	Novel genes
*ARL3*	rs10786679, rs2160203	0.55	1.91 × 10^−3^	4.48 × 10^−2^	3.07 × 10^−8^	5.90 × 10^−4^	2.58 × 10^− 4^	Novel genes

### GeneMANIA-based PPI network analysis of identified 31 asthma-associated genes

**Fig. 5:**
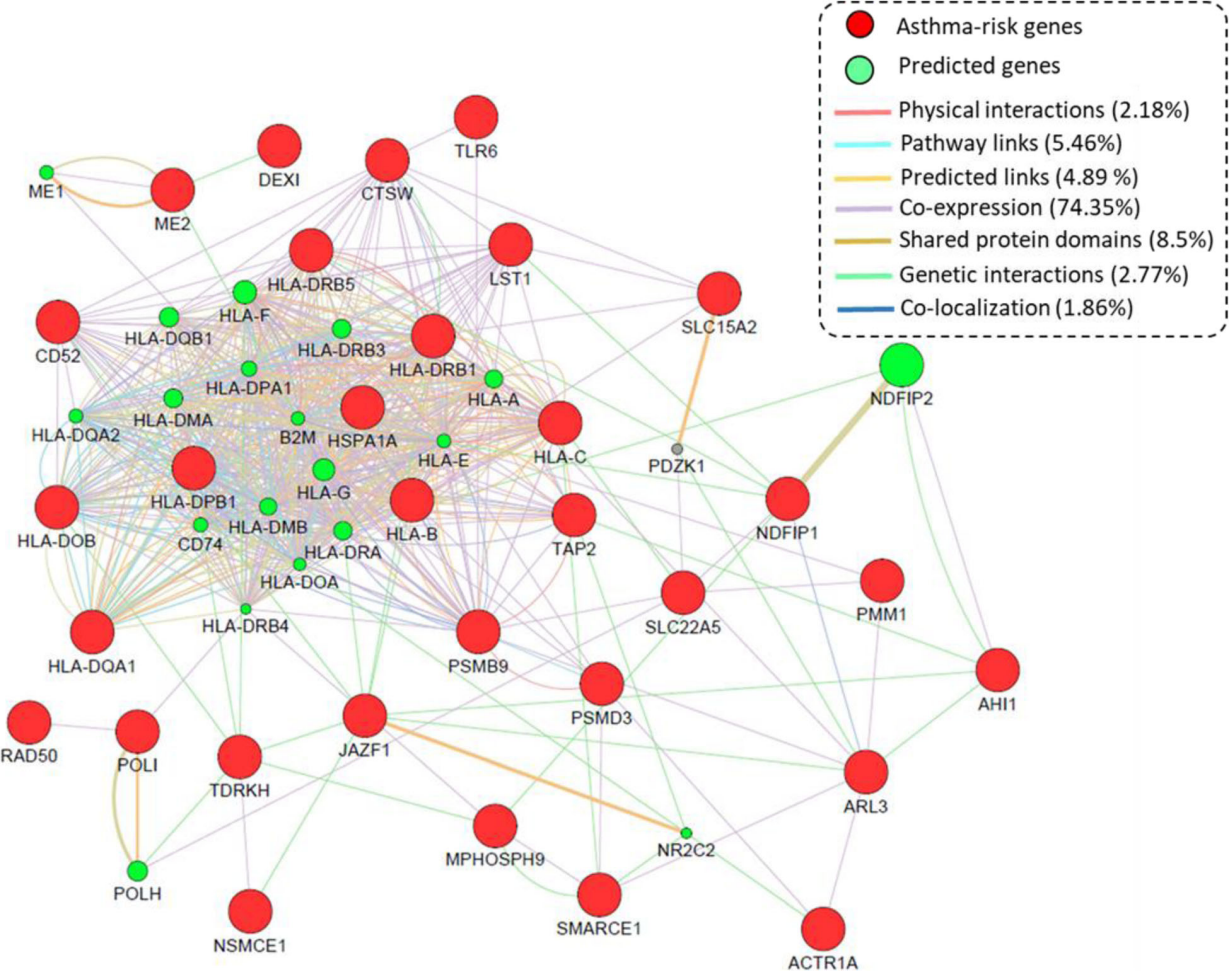
GeneMANIA-based PPI network of 31 identified childhood-onset asthma-relevant genes. The 31 asthma-associated risk genes are colored with red color, and the predicted genes are colored with green color. The underlying molecular links among these identified genes were attributed based on the physical interactions, pathway links, predicted links, co-expression, genetic interactions, co-localization, and shared protein domains

To further identify the underlying molecular links of these 31 childhood-onset asthma-associated genes, we conducted a GeneMANIA-based PPI network analysis via using multiple layers of existing evidence. Figure 5 shows that these identified risk genes are built a biological subnetwork, demonstrating that there were highly biological interactions among these susceptibility genes. The co-expression links among these identified genes account for the largest proportion of 74.35% (Fig. 5). The genes of *HLA-DRB5*, *HLA-DQA1*, *HLA-DPB1*, *CTSW*, *PSMB9*, and *TAP2* have the most number of edges with both predicted genes and childhood-onset asthma-related genes. For example, *PSMB9* gene showed a remarkably co-expression link with *TAP2* gene, as well as there existed a physical interaction between these two genes.

### Gene expression profiles of 31 identified genes between childhood-onset asthma and control groups

**Fig. 6:**
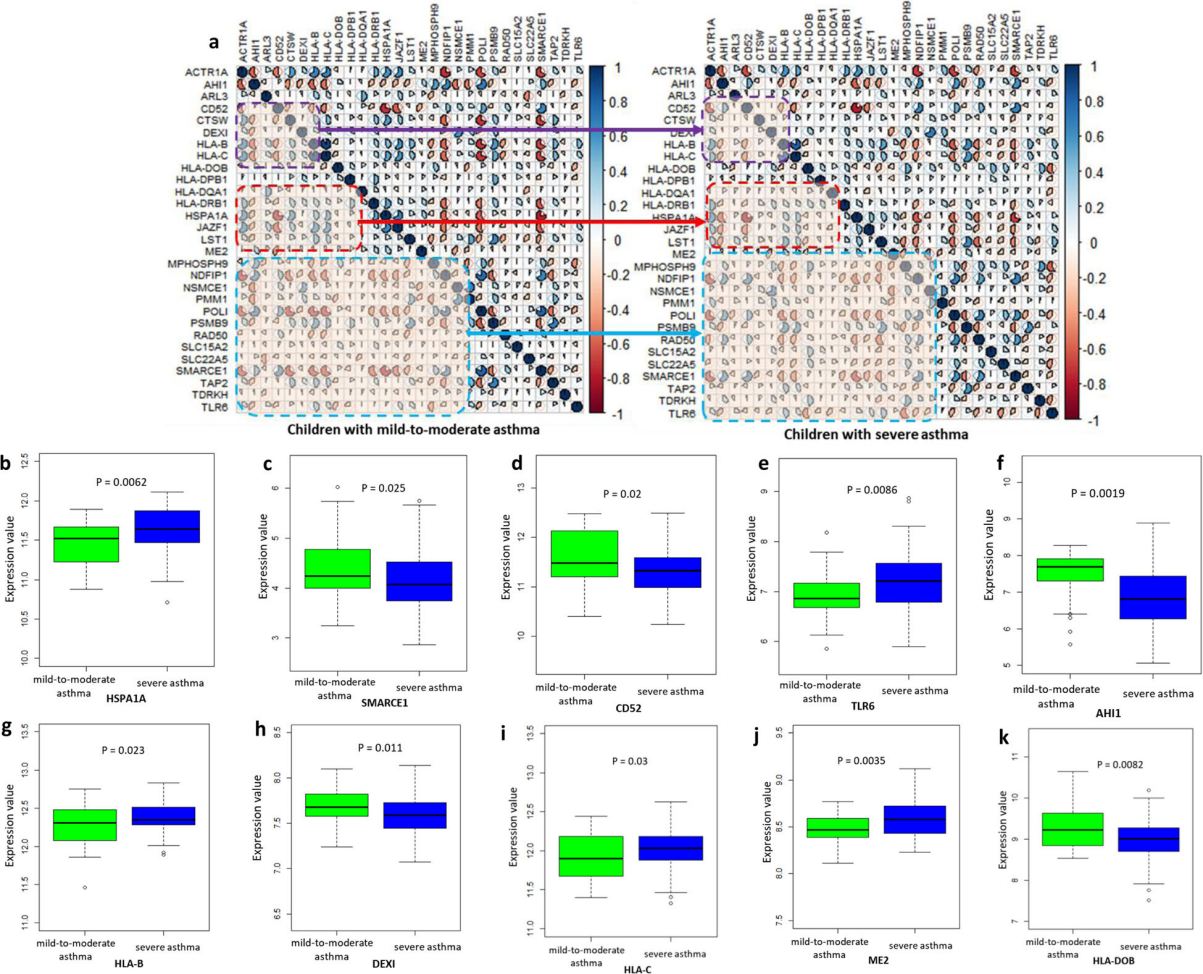
Differential expression profiles of 31 identified genes between mild-to-moderate asthma and severe asthma group. **a** Co-expression patterns of 31 identified genes between mild-to-moderate asthma and severe asthma group. **b**-**k** Boxplots show the differential expression profiles of 10 genes between mild-to-moderate asthma and severe asthma group. **b** HSPA1A, **c** SMARCE1, **d** CD52, **e** TLR6, **f** AHI1, **g** HLA-B, **h** DEXI, **i** HLA-C, **j** ME2, **k** HLA-DOB. The significance of each gene was calculated by using the Student’s t test

To test the co-expression patterns and differential gene expression of these 31 identified genes, we performed both co-expression analysis and differential gene expression (DGE) analysis in two RNA expression datasets (i.e., GSE123750 and GSE103166) on childhood-onset asthma. For the dataset of GSE123750, by using the Pearson correlation analysis, we observed that the co-expression patterns of 31 genes were obviously changed in severe asthma group compared with mild-to-moderate asthma group (*P* = 0.0024; Fig. 6a and Supplemental Fig. S5). Subsequently, by performing a DGE analysis, we found that the expression levels of 14 genes showed significant or suggestive differences in severe asthmatic samples compared with that in mild-to-moderate asthmatic samples (Fig. 6b-k and Supplemental Fig. S6a-d); For example, *HSPA1A* (*P* = 0.0062), *SMARCE1* (*P* = 0.025), *CD52* (*P* = 0.02), *TLR6* (*P* = 0.0086), and *AHI1* (*P* = 0.0019).

**Fig. 7:**
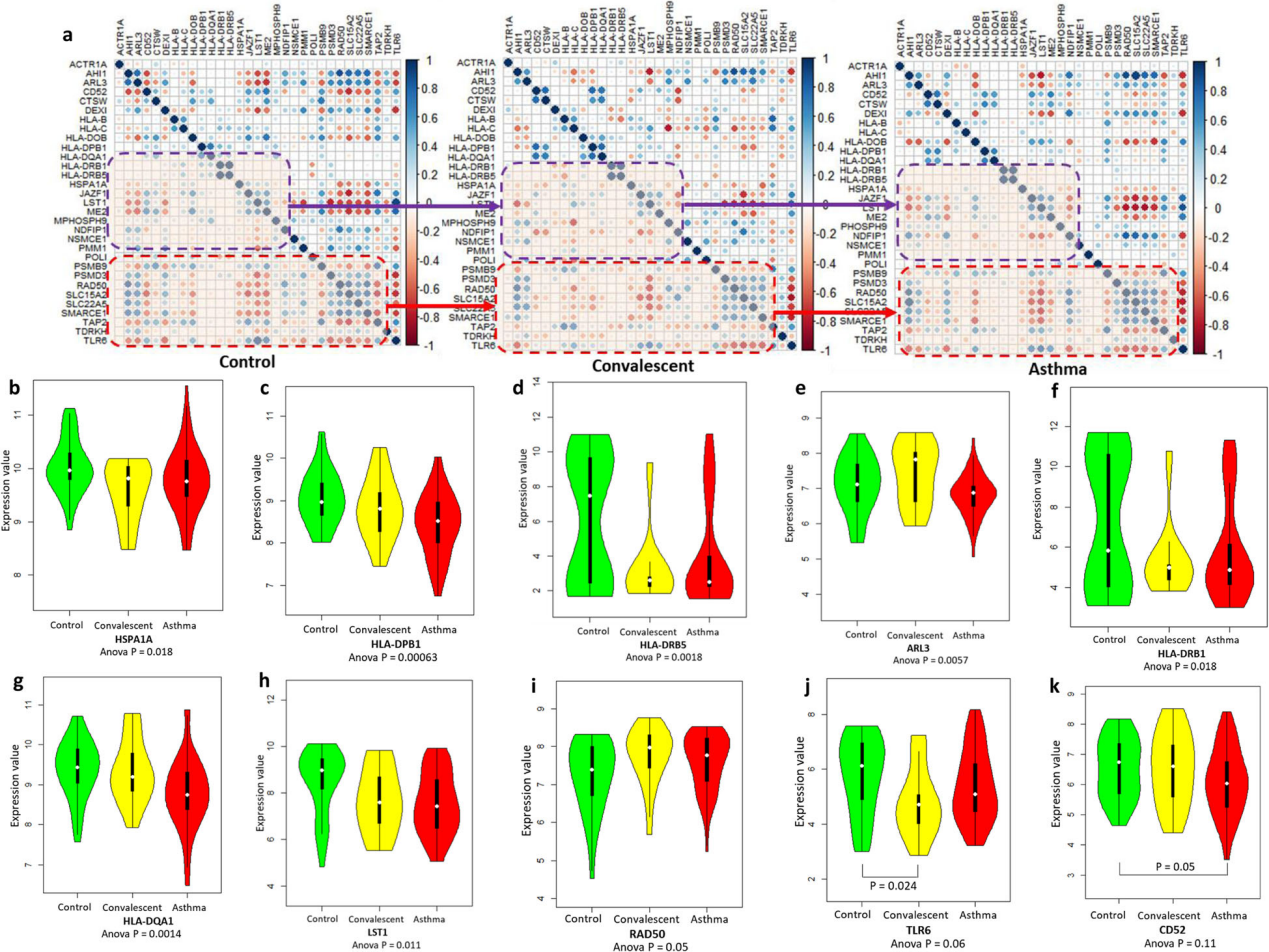
Differential expression profiles of 31 identified genes among control, convalescence, and severe asthma group. **a**) Co-expression patterns of 31 identified genes among control, convalescence, and severe asthma group. **b**) - **k**) Boxplots show the differential expression profiles of 10 genes among control, convalescence, and severe asthma group. **b** HSPA1A, **c** HLA-DPB1, **d** HLA-DRB5, **e** ARL3, **f** HLA-DRB1, **g** HLA-DQA1, **h** LST1, **i** RAD50, **j** TLR6, **k** CD52. The significance of each gene was calculated by using one-way ANOVA analysis

With regard to the dataset of GSE103166, we also applied the Pearson correlation analysis and identified the distinct co-expression patterns of 31 genes among control, convalescence, and asthma groups (Fig. 7a), which is consistent with our above results from the GSE123750 dataset. We further found 8 genes of *HSPA1A* (Anova *P* = 0.018), *HLA-DPB1* (Anova *P* = 0.00063), *HLA-DRB5* (Anova *P* = 0.0018), *ARL3* (Anova *P* = 0.0057), *HLA-DRB1* (Anova *P* = 0.018), *HLA-DQA1* (Anova *P* = 0.0014), *LST1* (Anova *P* = 0.011), and *RAD50* (Anova *P* = 0.05) were significantly differentially expressed across three groups (Fig. 7b-i), and detected other 8 genes showed suggestively differential expressions across three groups (Fig. 7j-k and Supplemental Fig. S7a-f).

## Discussion

Childhood-onset asthma is influenced by the combination of environmental and genetic factors [6–9]. Many GWASs have been conducted for revealing the genetic determinants underlying childhood-onset asthma [7, 13–21]. However, the detailed molecular functions of identified genetic variants on childhood-onset asthma risk remain largely ambiguous. Due to the cause of linkage disequilibrium (LD) between SNPs, these GWAS-identified SNPs to a large degree encompassed many highly LD SNPs with similar significant levels of association signals, which enhance the difficulty of pinpointing the causative variants. In addition, since GWASs for complex diseases have typically yielded a large number of genetic loci with limited annotations and no remarkable functional consequences, mostly located in noncoding regions [23, 24], it is reasonable to speculate that these variants prone to regulate RNA expression or transcription level of a specific gene rather than its protein function [48–51]. Alterations in RNA expression or transcription levels have important roles in complex diseases [12, 52, 53]. Since only depended on the typical GWAS for identifying the significant association signal of each single SNP is impossible to uncover the complex regulatory mechanisms of diseases-relevant SNPs, more comprehensive integrative genomics-based studies are needed to understand the genetic mechanisms of childhood-onset asthma susceptibility.

In the current investigation, we applied a two-stage designed integrative genomics analysis to reveal the functional effects of genetic variants from the whole genome on regulating transcriptional abundance as well as childhood-onset asthma risk. In the discovery stage, based on the Sherlock-based Bayesian analysis by integrating a large-scale GWAS summary dataset with an eQTL dataset, we identified 560 significant genes associated with childhood-onset asthma. Among these significant genes, 37 genes have been documented to be associated with asthma in the GWAS Catalog database. Furthermore, a recent GWAS study on childhood-onset asthma [15] reported by Pividori and coworkers has applied the PrediXcan method based on five tissues including whole blood, lung, skin, small intestine, and spleen to identify genes whose expressions were predicted by variants associated with asthma. The authors found 113 unique causal genes at 22 GWAS loci for asthma. Compared with their results, we found 40 of 113 genes were replicated by our Sherlock analysis (Supplemental Table S12). In addition, we also found that there were 26 genes reported to be associated with type 1 diabetes or rheumatoid arthritis (Supplemental Table S12). For example, the genes of *HLA-DRB1* [54–56], *TAP2* [57], *DEXI* [58], and *JAZF1* [59]. Consistently, we performed a MAGMA gene-based analysis of current-used GWAS for independent technical replication. There were 83 Sherlock-identified genes significantly replicated.

In addition, we selected six autoimmune diseases including type I diabetes, rheumatoid arthritis, multiple sclerosis, Crohn’s disease, Coeliac disease, and primary biliary cirrhosis with GWAS summary statistics from the UK-Biobank database to calculate the genetic correlations with childhood onset asthma by using LD score regression [60], and found there were non-significant LD regression scores between childhood onset asthma and six autoimmune diseases (Supplemental Table S13), which is in agreement with an earlier study on adult asthma and autoimmune diseases [18]. By performing a colocalization analysis using *coloc* R package [61], we found only a few of SNPs showed low or moderate posterior possibility between childhood onset asthma and six autoimmune diseases (Supplemental Table S14), suggesting that these identified association signals between risk genes and childhood-onset asthma not suffer remarkable influence from other autoimmune diseases.

Subsequently, we used these 83 identified genes to perform functional enrichment analyses, and identified a number of significant enriched pathways and GO-terms, including the pathways of Antigen processing and presentation, type I diabetes mellitus, and asthma. Further, based on the disease-based enrichment analysis, we observed these identified genes were overrepresented in gene sets associated with numerous diseases, including autoimmune diseases, immune system diseases, and asthma. These enriched functional terms, pathways and disease-related gene sets provide a reference clue for guiding future genetic or genomics-based researches. As the approach used in previous studies [31, 32], we re-conducted the Sherlock analysis in an independent eQTL dataset for biological replication. Among the 83 genes, there were 31 genes were significantly replicated. Furthermore, in silico permutation analysis showed that these identified disease-risk genes are attributed to genetic determinants rather than false positives or random events. Given that the basic concept of Sherlock integrative analysis is on the basis of abnormal expression or transcription levels of risk genes contribute risk to the development of complex diseases [62], we further carried out both co-expression analysis and DGE analysis in two independent RNA datasets and found that most of these 31 genes (25/31 = 80.65%) showed significantly or suggestively differential expressions according to asthma status. Taken together, the two-stage designed analysis used in the present study ensures the reliability and specificity of our findings.

Based on aforementioned systematical genomics analysis, we highlighted 31 convincing genes associated with childhood-onset asthma. Among them, 13 of 31 genes have been reported to be significantly associated with asthma-related phenotypes, including age of asthma onset, childhood-onset asthma, adult-onset asthma, diisocyanate-induced asthma, and pleiotropy of asthma and allergic diseases; namely, *HLA-DQA1* [15–18, 21, 63], *HLA-DRB5* [16, 63], *HLA-DRB1* [16, 18, 21], *TLR6* [15, 18], *MPHOSPH9* [16], *JAZF1* [15, 16, 64], *TDRKH* [16], *SMARCE1* [15, 16, 18], *RAD50* [16, 18, 63, 65–67], *HLA-B* [15, 16], *POLI* [16], *SLC22A5* [7, 15, 18, 21], and *NDFIP1* [16, 21, 63, 68]. Furthermore, it should be noted that 18 childhood-onset asthma-associated genes, which were not documented in the GWAS Catalog database, were newly identified from our current comprehensive genomics analysis. For example, the genes of *PSMB9*, *TAP2*, *HLA-DPB1*, *PMM1*, and *PSMD3*. Among these identified 31 convincing genes associated with childhood-onset asthma risk, we found that there existed multiple risk eSNPs associated with transcriptional abundance of a specific gene and disease risk per se simultaneously. To name a few, rs4148882 and rs2071534 exert cis effects on regulating the expression level of *PSMB9*. Rs9267798, rs4148882, and rs241456 also have cis-regulatory function in modulating the gene of *TAP2* expression. We noticed that the eSNP of rs4148882 has cis-regulatory roles in influencing both *PSMB9* and *TAP2* expression, indicating that these two genes may have convergent effects on childhood-onset asthma susceptibility, which is in line with the findings in our GeneMANIA-based PPI network analysis.

A growing number of studies have demonstrated that disease-associated genes with similar functions may collectively contribute risk to complex diseases [69–73], including asthma [74]. Consistently, our GeneMANIA-based PPI network analysis demonstrated that these 31 genes were highly interacted with each other based on multiple layers of evidence. For example, the hub gene of *PSMB9* is significantly co-expressed with identified genes of *TAP2*, *ARL3*, *TLR6*, *HLA-DRB1*, *CD52*, and *HLA-DOB*, and predicted genes of *ME1*, *CD74*, *HLA-G*, and *HLA-DRB4* based on previous reported studies [75–78]. Additionally, the identified childhood-onset asthma-associated gene of *JAZF1* has evidence of genetic interactions with identified genes of *AHI1*, *NSMCE1*, *TDRKH*, *CD52*, and *HLA-DRB1* based on a genome-wide map of genetic interaction inferred from radiation hybrid genotypes [79]. Although multiple evidence support there exist highly biological connections among these identified genes, it should be cautious that these biological relationships provided by GeneMANIA tool were based on multiple tissues, which were not filtered for tissues specifically related to asthma.

For the hub gene of *PSMB9*, which is located in the class II region of the major histocompatibility complex (MHC), its protein of proteasome is a multicatalytic proteinase complex with a highly ordered ring-shaped 20S core structure. Gamma interferon induced the expression of *PSMB9* gene, of which product replaces catalytic subunit 1 in the immunoproteasome. A recent genome-wide methylation study [80] indicated the top-associated CpG site of cg04908668 in the *PSMB9* gene might implicate in nitrogen dioxide (NO2)-exposure-related lung function damage or respiratory disease. Abnormal expressed of *PSMB9* and *TAP2* gene are prominently associated with POCD, and both *PSMB9* and *TAP2* gene accompanied with other COPD-expressed genes such as *PSMB8* and *TAP1* involved in the antigen processing and presentation pathway, which might change phenotypes of alveolar epithelial type II cells in COPD lungs [81]. Consistently, our results indicate the top-ranked pathway of antigen processing and presentation pathway enriched by identified risk genes potentially implicated in the aetiology of childhood-onset asthma. With regard to the hub gene of *TAP2*, it encodes a membrane-associated protein, which is a member of the superfamily of ATP-binding cassette (ABC) transporters. Many genetic variants in *TAP2* gene have been reported to contribute susceptibility to pulmonary tuberculosis [82, 83], diffuse panbronchiolitis [84], aspirin exacerbated respiratory disease [85], and idiopathic bronchiectasis [86]. Down-regulated expression of *TAP2* and *TAP1* may partially deficient HLA Class I expression and then deficient antigen processing in small cell lung cancer lines (SCLC) [87]. Together, these results indicate these identified eSNPs and risk genes are more likely to be functional candidates for further molecular experiments.

## Conclusions

In sum, current integrative genomics analysis provides an effective approach to connect genetic variants across the whole genome with genes through their cis- and/or trans-regulatory effects on expression, which is more biologically relevant and interpretable than a pure GWAS analysis for individual association signals. Based on multiple lines of evidence, we highlighted 31 genes including *PSMB9* and *TAP2* with multiple eSNPs as childhood-onset asthma-associated causative candidates. More molecular experiments are warranted to be conducted for uncovering the detailed biological mechanisms of these prioritized genes for childhood-onset asthma risk.

## Supplementary information

**Additional file 1: Table S1.** Sherlock Bayesian analysis identifies 560 genes as childhood-onset asthma-risk genes in discovery samples (FDR < 0.05, Dataset #2). **Table S2.** 83 Sherlock-identified genes from discovery Dataset #2 overlapped with MAGMA-identified genes. **Table S3.** Significant KEGG pathways enriched by childhood-onset asthma-relevant genes (*N* = 83). **Table S4.** Significant GO-terms of molecular function enriched by childhood-onset asthma-relevant genes (N = 83). **Table S5.** Significant GO-terms of cellular component enriched by childhood-onset asthma-relevant genes (N = 83). **Table S6.** Significant GO-terms of biological process enriched by childhood-onset asthma-relevant genes (N = 83). **Table S7.** Disease-related gene sets in GLAD4U database significantly enriched by childhood-onset asthma-relevant genes (N = 83). **Table S8.** Disease-related gene sets in DisGeNET database significantly enriched by childhood-onset asthma-relevant genes (N = 83). **Table S9.** Drug-related gene sets in GeneBank database significantly enriched by childhood-onset asthma-relevant genes (N = 83). **Table S10.** Drug-related gene sets in GLAD4U database significantly enriched by childhood-onset asthma-relevant genes (N = 83). **Table S11.** Multiple top-ranked eSNPs identified in 31 candidate genes implicated in childhood-onset asthma risk. **Table S12.** Sherlock-identified genes in the discovery stage reported in previous studies. **Table S13.** Genetic correlations between childhood onset asthma and other six autoimmune diseases. **Table S14.** Colocalization analysis for childhood onset asthma with other six autoimmune diseases.

**Additional file 2: Figure S1.** Disease-based enrichment analysis of 83 childhood-onset asthma-related genes based on the DisGeNET database. **Figure S2.** Drug-based enrichment analysis of 83 childhood-onset asthma-related genes based on the Drugbank database. **Figure S3.** Drug-based enrichment analysis of 83 childhood-onset asthma-related genes based on the GLAD4U database. **Figure S4.** Consistent evidence of childhood-onset asthma-relevant genes based on independent datasets and techniques. a) Venn diagram of three identified childhood-onset asthma-relevant gene sets. b) Computer-based permutation analysis of 10^5^ times for the comparison of genes from dataset #3 with that from MAGMA analysis (raw *P* value of each gene was applied). c) Computer-based permutation analysis of 10^5^ times for the comparison of genes from dataset #3 with that from dataset #4 (raw P value of each gene was applied). **Figure S5.** Density plot show the differences of co-expression patterns between childhood-onset asthma (CoA) and matched controls. **Figure S6.** Boxplots show the differential expression profiles of 4 genes between mild-to-moderate asthma and severe asthma group. **Figure S7.** Boxplots show the differential expression profiles of 6 genes among control, convalescence, and severe asthma group.

## Data Availability

GWAS summary dataset on childhood-onset was downloaded from the UK-Biobank resource (https://genepi.qimr.edu.au/staff/manuelF/gwas_results/main.html). The eQTL data for discovery was available in the official website (http://sherlock.ucsf.edu/submit.html, Zeller_10). The eQTL data for independent validation was available in the official website (http://sherlock.ucsf.edu/submit.html, Dixon_7). The RNA expression datasets (Accession Nos. GSE123750 and GSE103166) were downloaded from the NCBI GEO database (https://www.ncbi.nlm.nih.gov/geo/).

## Abbreviations

GWAS: Genome-Wide Association Studies
HWE: Hardy-Weinberg Equilibrium
GCR: Genotype Calling Rate
MAF: Minor Allele Frequency
eQTL: Expression quantitative trait loci
LBF: The Logarithm of the Bayes Factor
MAGMA: Multi-marker Analysis of GenoMic Annotation
LD: Linkage disequilibrium
DAVID: The Database for Annotation, Visualization, and Integrated Discovery
KEGG: The Kyoto Encyclopedia of Genes and Genomes
GO: Gene ontology
BP: Biological process
CC: Cellular component
MF: Molecular function
FDR: False discovery rate
WebGestalt: WEB-based Gene SeT AnaLysis Toolkit
PPI: Protein-protein interaction
GEO: The Gene Expression Omnibus repository
