# Supplementary material for: Integrative genomics analysis of various omics data and networks identify risk genes and variants vulnerable to childhood-onset asthma
Source: BMC Med Genomics. 2020 Aug 31;13:123. doi: 10.1186/s12920-020-00768-z (PMC7457797; doi:10.1186/s12920-020-00768-z)
Supplement: Supplementary file 1 — Additional file 1: Table S1. Sherlock Bayesian analysis identifies 560 genes as childhood-onset asthma-risk genes in discovery samples (FDR < 0.05, Dataset #2). Table S2. 83 Sherlock-identified genes from discovery Dataset #2 overlapped with MAGMA-identified genes. Table S3. Significant KEGG pathways enriched by childhood-onset asthma-relevant genes (N = 83). Table S4. Significant GO-terms of molecular function enriched by childhood-onset asthma-relevant genes (N = 83). Table S5. Significant GO-terms of cellular component enriched by childhood-onset asthma-relevant genes (N = 83). Table S6. Significant GO-terms of biological process enriched by childhood-onset asthma-relevant genes (N = 83). Table S7. Disease-related gene sets in GLAD4U database significantly enriched by childhood-onset asthma-relevant genes (N = 83). Table S8. Disease-related gene sets in DisGeNET database significantly enriched by childhood-onset asthma-relevant genes (N = 83). Table S9. Drug-related gene sets in GeneBank database significantly enriched by childhood-onset asthma-relevant genes (N = 83). Table S10. Drug-related gene sets in GLAD4U database significantly enriched by childhood-onset asthma-relevant genes (N = 83). Table S11. Multiple top-ranked eSNPs identified in 31 candidate genes implicated in childhood-onset asthma risk. Table S12. Sherlock-identified genes in the discovery stage reported in previous studies. Table S13. Genetic correlations between childhood onset asthma and other six autoimmune diseases. Table S14. Colocalization analysis for childhood onset asthma with other six autoimmune diseases. [file 12920_2020_768_MOESM1_ESM.docx]

**Supplemental Tables**

**Supplemental Table S1**. **Sherlock Bayesian analysis identifies 560 genes as childhood-onset asthma-risk genes in discovery samples (FDR < 0.05, Dataset #2)**

| **Gene Name** | **LBF** | **Sherlock-based P-value** | **FDR-value** | **GWAS Catalog** |
| --- | --- | --- | --- | --- |
| *HLA-DRB3* | 13.46 | 7.87E-07 | 2.05E-04 | Non-documented gene |
| *HLA-DQA1* | 12.91 | 7.87E-07 | 2.05E-04 | Reported gene |
| *HLA-DRB4* | 12.71 | 7.87E-07 | 2.05E-04 | Non-documented gene |
| *NOTCH4* | 12.37 | 7.87E-07 | 2.05E-04 | Reported gene |
| *PSMB9* | 12.17 | 7.87E-07 | 2.05E-04 | Non-documented gene |
| *FALZ* | 11.59 | 7.87E-07 | 2.05E-04 | Non-documented gene |
| *HLA-DRB5* | 11.52 | 7.87E-07 | 2.05E-04 | Reported gene |
| *HLA-DPB1* | 11.26 | 7.87E-07 | 2.05E-04 | Non-documented gene |
| *HLA-DRB1* | 10.91 | 7.87E-07 | 2.05E-04 | Reported gene |
| *EBI2* | 9.48 | 7.87E-07 | 2.05E-04 | Non-documented gene |
| *MAP2K5* | 9.18 | 7.87E-07 | 2.05E-04 | Non-documented gene |
| *VARS2* | 9.00 | 7.87E-07 | 2.05E-04 | Non-documented gene |
| *HLA-DMA* | 8.79 | 7.87E-07 | 2.05E-04 | Non-documented gene |
| *TAP2* | 8.70 | 7.87E-07 | 2.05E-04 | Non-documented gene |
| *LGALS3BP* | 7.90 | 7.87E-07 | 2.05E-04 | Non-documented gene |
| *KRT83* | 7.85 | 7.87E-07 | 2.05E-04 | Non-documented gene |
| *CRKRS* | 7.85 | 7.87E-07 | 2.05E-04 | Non-documented gene |
| *ADORA1* | 7.79 | 7.87E-07 | 2.05E-04 | Reported gene |
| *HCG27* | 7.72 | 7.87E-07 | 2.05E-04 | Non-documented gene |
| *SERPINE1* | 7.70 | 7.87E-07 | 2.05E-04 | Non-documented gene |
| *APOM* | 7.49 | 7.87E-07 | 2.05E-04 | Non-documented gene |
| *TLR6* | 7.38 | 7.87E-07 | 2.05E-04 | Reported gene |
| *PMM1* | 7.36 | 7.87E-07 | 2.05E-04 | Non-documented gene |
| *HLA-A29.1* | 7.25 | 7.87E-07 | 2.05E-04 | Non-documented gene |
| *IL18R1* | 7.22 | 7.87E-07 | 2.05E-04 | Reported gene |
| *MPHOSPH9* | 7.20 | 7.87E-07 | 2.05E-04 | Reported gene |
| *LYPLA3* | 7.20 | 7.87E-07 | 2.05E-04 | Non-documented gene |
| *SUOX* | 7.11 | 7.87E-07 | 2.05E-04 | Reported gene |
| *IKZF3* | 7.02 | 7.87E-07 | 2.05E-04 | Reported gene |
| *ZNF76* | 6.96 | 7.87E-07 | 2.05E-04 | Non-documented gene |
| *BCL6* | 6.95 | 7.87E-07 | 2.05E-04 | Non-documented gene |
| *PSMD3* | 6.83 | 1.57E-06 | 2.05E-04 | Non-documented gene |
| *PERLD1* | 6.83 | 1.57E-06 | 2.05E-04 | Non-documented gene |
| *STARD3* | 6.81 | 1.57E-06 | 2.05E-04 | Non-documented gene |
| *MSH5* | 6.80 | 1.57E-06 | 2.05E-04 | Non-documented gene |
| *ID2* | 6.73 | 1.57E-06 | 2.05E-04 | Non-documented gene |
| *MEI1* | 6.70 | 1.57E-06 | 2.05E-04 | Non-documented gene |
| *BHLHB2* | 6.69 | 1.57E-06 | 2.05E-04 | Non-documented gene |
| *HLA-DOB* | 6.67 | 1.57E-06 | 2.05E-04 | Non-documented gene |
| *NSF* | 6.66 | 1.57E-06 | 2.05E-04 | Non-documented gene |
| *TNFSF4* | 6.60 | 1.57E-06 | 2.05E-04 | Reported gene |
| *ME2* | 6.60 | 1.57E-06 | 2.05E-04 | Non-documented gene |
| *PHF5A* | 6.56 | 1.57E-06 | 2.05E-04 | Reported gene |
| *ACTR1A* | 6.55 | 1.57E-06 | 2.05E-04 | Non-documented gene |
| *GNGT2* | 6.48 | 1.57E-06 | 2.05E-04 | Non-documented gene |
| *NOL3* | 6.48 | 1.57E-06 | 2.05E-04 | Non-documented gene |
| *SERPINB2* | 6.45 | 1.57E-06 | 2.05E-04 | Non-documented gene |
| *DEXI* | 6.42 | 1.57E-06 | 2.05E-04 | Non-documented gene |
| *RERE* | 6.40 | 1.57E-06 | 2.05E-04 | Reported gene |
| *EAF2* | 6.37 | 1.57E-06 | 2.05E-04 | Non-documented gene |
| *D2HGDH* | 6.34 | 1.57E-06 | 2.05E-04 | Reported gene |
| *RUVBL1* | 6.30 | 1.57E-06 | 2.05E-04 | Non-documented gene |
| *DPEP2* | 6.26 | 1.57E-06 | 2.05E-04 | Non-documented gene |
| *MUS81* | 6.26 | 1.57E-06 | 2.05E-04 | Non-documented gene |
| *BRD2* | 6.25 | 1.57E-06 | 2.05E-04 | Reported gene |
| *IER3* | 6.23 | 1.57E-06 | 2.05E-04 | Non-documented gene |
| *NSMCE1* | 6.22 | 1.57E-06 | 2.05E-04 | Non-documented gene |
| *JAZF1* | 6.18 | 1.57E-06 | 2.05E-04 | Reported gene |
| *TDRKH* | 6.18 | 1.57E-06 | 2.05E-04 | Reported gene |
| *IL4R* | 6.15 | 1.57E-06 | 2.05E-04 | Reported gene |
| *HLA-DRB6* | 6.14 | 1.57E-06 | 2.05E-04 | Reported gene |
| *SLC22A4* | 6.13 | 1.57E-06 | 2.05E-04 | Non-documented gene |
| *LCAT* | 6.12 | 1.57E-06 | 2.05E-04 | Non-documented gene |
| *HCLS1* | 6.08 | 1.57E-06 | 2.05E-04 | Non-documented gene |
| *STAT6* | 6.08 | 1.57E-06 | 2.05E-04 | Reported gene |
| *SMARCE1* | 6.06 | 1.57E-06 | 2.05E-04 | Reported gene |
| *DPEP3* | 6.03 | 1.57E-06 | 2.05E-04 | Non-documented gene |
| *DEF6* | 6.03 | 1.57E-06 | 2.05E-04 | Non-documented gene |
| *JMJD5* | 6.02 | 1.57E-06 | 2.05E-04 | Non-documented gene |
| *GLB1* | 6.01 | 1.57E-06 | 2.05E-04 | Reported gene |
| *GFOD2* | 5.96 | 1.57E-06 | 2.05E-04 | Non-documented gene |
| *PARD6A* | 5.94 | 1.57E-06 | 2.05E-04 | Non-documented gene |
| *AHI1* | 5.89 | 1.57E-06 | 2.05E-04 | Non-documented gene |
| *CSNK2B* | 5.81 | 1.57E-06 | 2.05E-04 | Non-documented gene |
| *BTN3A2* | 5.81 | 1.57E-06 | 2.05E-04 | Non-documented gene |
| *FCER1G* | 5.79 | 1.57E-06 | 2.05E-04 | Reported gene |
| *PSMD5* | 5.77 | 1.57E-06 | 2.05E-04 | Non-documented gene |
| *SLC15A2* | 5.77 | 1.57E-06 | 2.05E-04 | Non-documented gene |
| *C5* | 5.76 | 1.57E-06 | 2.05E-04 | Non-documented gene |
| *RAD51L1* | 5.73 | 1.57E-06 | 2.05E-04 | Non-documented gene |
| *IRF1* | 5.72 | 1.57E-06 | 2.05E-04 | Non-documented gene |
| *ZNRF1* | 5.66 | 1.57E-06 | 2.05E-04 | Non-documented gene |
| *CXCL1* | 5.63 | 1.57E-06 | 2.05E-04 | Non-documented gene |
| *RAD50* | 5.60 | 1.57E-06 | 2.05E-04 | Reported gene |
| *GSN* | 5.60 | 1.57E-06 | 2.05E-04 | Non-documented gene |
| *TGIF2* | 5.56 | 1.57E-06 | 2.05E-04 | Non-documented gene |
| *DNAJC7* | 5.55 | 1.57E-06 | 2.05E-04 | Non-documented gene |
| *HSPA1B* | 5.54 | 1.57E-06 | 2.05E-04 | Non-documented gene |
| *TCF25* | 5.52 | 1.57E-06 | 2.05E-04 | Non-documented gene |
| *FADS6* | 5.50 | 1.57E-06 | 2.05E-04 | Non-documented gene |
| *HLA-B* | 5.50 | 1.57E-06 | 2.05E-04 | Reported gene |
| *COL9A2* | 5.45 | 1.57E-06 | 2.05E-04 | Non-documented gene |
| *DENND1A* | 5.36 | 1.57E-06 | 2.05E-04 | Non-documented gene |
| *MYBPH* | 5.36 | 1.57E-06 | 2.05E-04 | Non-documented gene |
| *MANBA* | 5.30 | 1.57E-06 | 2.05E-04 | Non-documented gene |
| *MTMR11* | 5.27 | 1.57E-06 | 2.05E-04 | Non-documented gene |
| *MAP3K11* | 5.26 | 1.57E-06 | 2.05E-04 | Non-documented gene |
| *PSMD13* | 5.22 | 3.15E-06 | 4.03E-04 | Non-documented gene |
| *IQCB1* | 5.21 | 3.15E-06 | 4.03E-04 | Non-documented gene |
| *UMPS* | 5.19 | 4.72E-06 | 5.98E-04 | Non-documented gene |
| *LST1* | 5.14 | 6.30E-06 | 7.39E-04 | Non-documented gene |
| *POLI* | 5.12 | 6.30E-06 | 7.39E-04 | Reported gene |
| *AMMECR1* | 5.04 | 6.30E-06 | 7.39E-04 | Non-documented gene |
| *MICA* | 5.03 | 6.30E-06 | 7.39E-04 | Non-documented gene |
| *MYO5C* | 4.99 | 6.30E-06 | 7.39E-04 | Non-documented gene |
| *TOMM40L* | 4.98 | 6.30E-06 | 7.39E-04 | Non-documented gene |
| *CRIP1* | 4.98 | 6.30E-06 | 7.39E-04 | Non-documented gene |
| *KLHL5* | 4.96 | 6.30E-06 | 7.39E-04 | Reported gene |
| *ARSG* | 4.96 | 7.87E-06 | 8.10E-04 | Non-documented gene |
| *MBIP* | 4.93 | 7.87E-06 | 8.10E-04 | Non-documented gene |
| *CIC* | 4.92 | 7.87E-06 | 8.10E-04 | Non-documented gene |
| *VPS13D* | 4.87 | 7.87E-06 | 8.10E-04 | Non-documented gene |
| *RPL11* | 4.87 | 7.87E-06 | 8.10E-04 | Non-documented gene |
| *CAMKK2* | 4.83 | 7.87E-06 | 8.10E-04 | Non-documented gene |
| *NCOA1* | 4.83 | 7.87E-06 | 8.10E-04 | Non-documented gene |
| *ITPR3* | 4.81 | 7.87E-06 | 8.10E-04 | Non-documented gene |
| *PHF19* | 4.81 | 7.87E-06 | 8.10E-04 | Non-documented gene |
| *HSPA1A* | 4.80 | 7.87E-06 | 8.10E-04 | Non-documented gene |
| *DUS2L* | 4.79 | 7.87E-06 | 8.10E-04 | Non-documented gene |
| *MAPK10* | 4.79 | 7.87E-06 | 8.10E-04 | Non-documented gene |
| *INTS4* | 4.78 | 7.87E-06 | 8.10E-04 | Non-documented gene |
| *PARP3* | 4.75 | 7.87E-06 | 8.10E-04 | Non-documented gene |
| *CD1E* | 4.72 | 7.87E-06 | 8.10E-04 | Non-documented gene |
| *WBSCR22* | 4.68 | 9.45E-06 | 9.57E-04 | Non-documented gene |
| *TIPARP* | 4.65 | 9.45E-06 | 9.57E-04 | Non-documented gene |
| *RPAP1* | 4.61 | 1.10E-05 | 1.10E-03 | Non-documented gene |
| *DCAKD* | 4.59 | 1.10E-05 | 1.10E-03 | Non-documented gene |
| *CHST3* | 4.57 | 1.26E-05 | 1.25E-03 | Non-documented gene |
| *RBM26* | 4.55 | 1.42E-05 | 1.33E-03 | Non-documented gene |
| *CD160* | 4.54 | 1.42E-05 | 1.33E-03 | Non-documented gene |
| *SLC22A5* | 4.54 | 1.42E-05 | 1.33E-03 | Reported gene |
| *MPZ* | 4.54 | 1.42E-05 | 1.33E-03 | Non-documented gene |
| *NBPF20* | 4.53 | 1.42E-05 | 1.33E-03 | Non-documented gene |
| *KCTD11* | 4.50 | 1.42E-05 | 1.33E-03 | Non-documented gene |
| *ZNF20* | 4.50 | 1.42E-05 | 1.33E-03 | Non-documented gene |
| *RPA2* | 4.44 | 1.57E-05 | 1.44E-03 | Non-documented gene |
| *DOK2* | 4.42 | 1.57E-05 | 1.44E-03 | Non-documented gene |
| *TTC27* | 4.40 | 1.57E-05 | 1.44E-03 | Non-documented gene |
| *ZIC5* | 4.36 | 1.89E-05 | 1.64E-03 | Non-documented gene |
| *TUBB2A* | 4.33 | 1.89E-05 | 1.64E-03 | Non-documented gene |
| *CCDC4* | 4.32 | 1.89E-05 | 1.64E-03 | Non-documented gene |
| *RAB11FIP1* | 4.32 | 1.89E-05 | 1.64E-03 | Non-documented gene |
| *RTCD1* | 4.31 | 1.89E-05 | 1.64E-03 | Non-documented gene |
| *ZC3H10* | 4.29 | 1.89E-05 | 1.64E-03 | Non-documented gene |
| *MADCAM1* | 4.28 | 1.89E-05 | 1.64E-03 | Non-documented gene |
| *NFYA* | 4.28 | 1.89E-05 | 1.64E-03 | Non-documented gene |
| *TRAF1* | 4.26 | 2.05E-05 | 1.75E-03 | Non-documented gene |
| *CCDC66* | 4.22 | 2.05E-05 | 1.75E-03 | Non-documented gene |
| *TUFM* | 4.21 | 2.20E-05 | 1.84E-03 | Non-documented gene |
| *SLC7A6* | 4.20 | 2.20E-05 | 1.84E-03 | Non-documented gene |
| *SSRP1* | 4.20 | 2.20E-05 | 1.84E-03 | Non-documented gene |
| *ZNF576* | 4.16 | 2.52E-05 | 2.06E-03 | Non-documented gene |
| *NCSTN* | 4.16 | 2.52E-05 | 2.06E-03 | Non-documented gene |
| *CTSW* | 4.15 | 2.52E-05 | 2.06E-03 | Non-documented gene |
| *ZFAND6* | 4.15 | 2.52E-05 | 2.06E-03 | Non-documented gene |
| *SF3A2* | 4.14 | 2.83E-05 | 2.24E-03 | Non-documented gene |
| *REC8* | 4.14 | 2.83E-05 | 2.24E-03 | Non-documented gene |
| *CLK3* | 4.12 | 2.83E-05 | 2.24E-03 | Non-documented gene |
| *EFEMP2* | 4.12 | 2.83E-05 | 2.24E-03 | Non-documented gene |
| *SDSL* | 4.11 | 2.83E-05 | 2.24E-03 | Non-documented gene |
| *OVCH1* | 4.10 | 3.15E-05 | 2.42E-03 | Non-documented gene |
| *CCT6A* | 4.09 | 3.15E-05 | 2.42E-03 | Non-documented gene |
| *TMEM116* | 4.08 | 3.15E-05 | 2.42E-03 | Non-documented gene |
| *FAM3A* | 4.08 | 3.15E-05 | 2.42E-03 | Non-documented gene |
| *SLC7A4* | 4.06 | 3.15E-05 | 2.42E-03 | Non-documented gene |
| *CTDSPL2* | 4.06 | 3.31E-05 | 2.48E-03 | Non-documented gene |
| *RGP1* | 4.06 | 3.31E-05 | 2.48E-03 | Non-documented gene |
| *EPC1* | 4.06 | 3.31E-05 | 2.48E-03 | Non-documented gene |
| *RPL32P3* | 4.05 | 3.31E-05 | 2.48E-03 | Non-documented gene |
| *CACNA1H* | 4.04 | 3.46E-05 | 2.53E-03 | Non-documented gene |
| *HNRPDL* | 4.04 | 3.46E-05 | 2.53E-03 | Non-documented gene |
| *MYADM* | 4.03 | 3.46E-05 | 2.53E-03 | Non-documented gene |
| *OR7C2* | 4.03 | 3.46E-05 | 2.53E-03 | Non-documented gene |
| *ABCB7* | 4.03 | 3.62E-05 | 2.60E-03 | Non-documented gene |
| *PDIK1L* | 4.03 | 3.62E-05 | 2.60E-03 | Non-documented gene |
| *AYP1P1* | 4.03 | 3.62E-05 | 2.60E-03 | Non-documented gene |
| *POU2F1* | 3.99 | 3.78E-05 | 2.70E-03 | Non-documented gene |
| *GTF2I* | 3.94 | 4.25E-05 | 2.99E-03 | Non-documented gene |
| *HEXIM2* | 3.94 | 4.25E-05 | 2.99E-03 | Non-documented gene |
| *NDFIP1* | 3.93 | 4.25E-05 | 2.99E-03 | Reported gene |
| *GCNT1* | 3.92 | 4.72E-05 | 3.28E-03 | Non-documented gene |
| *ENDOG* | 3.91 | 4.72E-05 | 3.28E-03 | Non-documented gene |
| *SERPINB10* | 3.91 | 4.88E-05 | 3.36E-03 | Non-documented gene |
| *LRRC41* | 3.91 | 4.88E-05 | 3.36E-03 | Non-documented gene |
| *TRIM56* | 3.90 | 5.04E-05 | 3.45E-03 | Non-documented gene |
| *LRRC33* | 3.87 | 5.20E-05 | 3.45E-03 | Non-documented gene |
| *FLT3LG* | 3.86 | 5.20E-05 | 3.45E-03 | Non-documented gene |
| *BAZ2A* | 3.86 | 5.20E-05 | 3.45E-03 | Non-documented gene |
| *HDC* | 3.84 | 5.20E-05 | 3.45E-03 | Non-documented gene |
| *PLA2G10* | 3.83 | 5.20E-05 | 3.45E-03 | Non-documented gene |
| *SPNS1* | 3.83 | 5.20E-05 | 3.45E-03 | Non-documented gene |
| *GALNT11* | 3.81 | 5.35E-05 | 3.53E-03 | Non-documented gene |
| *DEF8* | 3.76 | 5.51E-05 | 3.60E-03 | Non-documented gene |
| *MAL* | 3.74 | 5.51E-05 | 3.60E-03 | Non-documented gene |
| *CRSP2* | 3.74 | 5.67E-05 | 3.68E-03 | Non-documented gene |
| *HLA-C* | 3.71 | 5.83E-05 | 3.73E-03 | Non-documented gene |
| *PRCP* | 3.71 | 5.83E-05 | 3.73E-03 | Non-documented gene |
| *MMP1* | 3.70 | 5.83E-05 | 3.73E-03 | Non-documented gene |
| *PROM1* | 3.66 | 6.30E-05 | 3.97E-03 | Non-documented gene |
| *AOAH* | 3.66 | 6.30E-05 | 3.97E-03 | Non-documented gene |
| *ALG5* | 3.65 | 6.30E-05 | 3.97E-03 | Non-documented gene |
| *THOC5* | 3.63 | 6.77E-05 | 4.14E-03 | Non-documented gene |
| *ZNHIT2* | 3.62 | 6.77E-05 | 4.14E-03 | Non-documented gene |
| *RPS26P10* | 3.62 | 6.77E-05 | 4.14E-03 | Non-documented gene |
| *FBLN2* | 3.62 | 6.77E-05 | 4.14E-03 | Non-documented gene |
| *NBPF10* | 3.61 | 6.77E-05 | 4.14E-03 | Non-documented gene |
| *SULT1A2* | 3.61 | 6.77E-05 | 4.14E-03 | Non-documented gene |
| *DUSP10* | 3.56 | 8.03E-05 | 4.82E-03 | Non-documented gene |
| *SERPINF1* | 3.56 | 8.03E-05 | 4.82E-03 | Non-documented gene |
| *VWCE* | 3.54 | 8.19E-05 | 4.82E-03 | Non-documented gene |
| *ESPN* | 3.52 | 8.19E-05 | 4.82E-03 | Non-documented gene |
| *SERPINB11* | 3.50 | 8.19E-05 | 4.82E-03 | Non-documented gene |
| *ANKRD47* | 3.50 | 8.19E-05 | 4.82E-03 | Non-documented gene |
| *HSD17B10* | 3.50 | 8.19E-05 | 4.82E-03 | Non-documented gene |
| *TRPC4* | 3.49 | 8.19E-05 | 4.82E-03 | Non-documented gene |
| *PRMT7* | 3.48 | 8.35E-05 | 4.90E-03 | Non-documented gene |
| *EIF4EBP2* | 3.47 | 8.50E-05 | 4.91E-03 | Non-documented gene |
| *B9D2* | 3.45 | 8.50E-05 | 4.91E-03 | Non-documented gene |
| *TINP1* | 3.44 | 8.50E-05 | 4.91E-03 | Non-documented gene |
| *FCER1A* | 3.43 | 8.66E-05 | 4.92E-03 | Non-documented gene |
| *ZFP57* | 3.43 | 8.66E-05 | 4.92E-03 | Non-documented gene |
| *N6AMT1* | 3.41 | 8.66E-05 | 4.92E-03 | Non-documented gene |
| *GSK3B* | 3.41 | 8.66E-05 | 4.92E-03 | Non-documented gene |
| *ZNF672* | 3.35 | 9.13E-05 | 5.13E-03 | Non-documented gene |
| *BRUNOL6* | 3.32 | 9.45E-05 | 5.13E-03 | Non-documented gene |
| *LRRC3* | 3.30 | 9.45E-05 | 5.13E-03 | Non-documented gene |
| *FABP2* | 3.29 | 9.45E-05 | 5.13E-03 | Non-documented gene |
| *SUSD3* | 3.29 | 9.45E-05 | 5.13E-03 | Non-documented gene |
| *SASH1* | 3.28 | 9.45E-05 | 5.13E-03 | Non-documented gene |
| *AIG1* | 3.28 | 9.45E-05 | 5.13E-03 | Non-documented gene |
| *HCG4* | 3.28 | 9.45E-05 | 5.13E-03 | Non-documented gene |
| *RDH8* | 3.27 | 9.61E-05 | 5.13E-03 | Non-documented gene |
| *ORMDL1* | 3.27 | 9.61E-05 | 5.13E-03 | Non-documented gene |
| *ADCK1* | 3.26 | 9.61E-05 | 5.13E-03 | Non-documented gene |
| *CCND2* | 3.26 | 9.61E-05 | 5.13E-03 | Non-documented gene |
| *RBMS2* | 3.26 | 9.61E-05 | 5.13E-03 | Non-documented gene |
| *USF1* | 3.26 | 9.61E-05 | 5.13E-03 | Non-documented gene |
| *AS3MT* | 3.24 | 9.76E-05 | 5.19E-03 | Non-documented gene |
| *CNP* | 3.24 | 9.92E-05 | 5.26E-03 | Non-documented gene |
| *GFRA2* | 3.23 | 1.01E-04 | 5.32E-03 | Reported gene |
| *DNASE1L1* | 3.22 | 1.02E-04 | 5.33E-03 | Non-documented gene |
| *RAB14* | 3.22 | 1.02E-04 | 5.33E-03 | Non-documented gene |
| *SART1* | 3.22 | 1.02E-04 | 5.33E-03 | Non-documented gene |
| *PROK2* | 3.21 | 1.06E-04 | 5.41E-03 | Non-documented gene |
| *SOX15* | 3.20 | 1.06E-04 | 5.41E-03 | Non-documented gene |
| *DNAH5* | 3.20 | 1.06E-04 | 5.41E-03 | Non-documented gene |
| *CSDE1* | 3.20 | 1.06E-04 | 5.41E-03 | Non-documented gene |
| *HERC4* | 3.19 | 1.10E-04 | 5.63E-03 | Non-documented gene |
| *EIF4E1B* | 3.16 | 1.15E-04 | 5.85E-03 | Non-documented gene |
| *SERPINA1* | 3.13 | 1.18E-04 | 5.98E-03 | Non-documented gene |
| *PSTPIP1* | 3.12 | 1.26E-04 | 6.28E-03 | Non-documented gene |
| *NEK6* | 3.12 | 1.26E-04 | 6.28E-03 | Non-documented gene |
| *PRPF38B* | 3.12 | 1.26E-04 | 6.28E-03 | Non-documented gene |
| *PCSK7* | 3.10 | 1.26E-04 | 6.28E-03 | Non-documented gene |
| *PID1* | 3.08 | 1.28E-04 | 6.33E-03 | Non-documented gene |
| *HLA-A* | 3.07 | 1.29E-04 | 6.34E-03 | Non-documented gene |
| *FZD7* | 3.06 | 1.29E-04 | 6.34E-03 | Non-documented gene |
| *JMJD1A* | 3.05 | 1.29E-04 | 6.34E-03 | Non-documented gene |
| *DRAM* | 3.04 | 1.31E-04 | 6.39E-03 | Non-documented gene |
| *MYST4* | 3.03 | 1.32E-04 | 6.42E-03 | Non-documented gene |
| *UBXD5* | 3.02 | 1.32E-04 | 6.42E-03 | Non-documented gene |
| *MICB* | 3.02 | 1.34E-04 | 6.47E-03 | Reported gene |
| *CCDC55* | 3.01 | 1.35E-04 | 6.52E-03 | Non-documented gene |
| *FTHL12* | 3.00 | 1.37E-04 | 6.52E-03 | Non-documented gene |
| *NPAT* | 3.00 | 1.37E-04 | 6.52E-03 | Non-documented gene |
| *GLI3* | 3.00 | 1.37E-04 | 6.52E-03 | Reported gene |
| *EPN1* | 3.00 | 1.39E-04 | 6.52E-03 | Non-documented gene |
| *MTERFD1* | 2.99 | 1.40E-04 | 6.52E-03 | Non-documented gene |
| *PPFIA4* | 2.99 | 1.40E-04 | 6.52E-03 | Non-documented gene |
| *SLC46A3* | 2.98 | 1.40E-04 | 6.52E-03 | Non-documented gene |
| *FAM86C* | 2.98 | 1.40E-04 | 6.52E-03 | Non-documented gene |
| *LASS5* | 2.96 | 1.40E-04 | 6.52E-03 | Non-documented gene |
| *HLA-G* | 2.95 | 1.43E-04 | 6.65E-03 | Non-documented gene |
| *MAML2* | 2.93 | 1.48E-04 | 6.84E-03 | Non-documented gene |
| *ANKRD34A* | 2.90 | 1.51E-04 | 6.96E-03 | Non-documented gene |
| *ARHGAP12* | 2.88 | 1.54E-04 | 7.08E-03 | Non-documented gene |
| *TRPC2* | 2.87 | 1.59E-04 | 7.24E-03 | Non-documented gene |
| *ALDH2* | 2.87 | 1.59E-04 | 7.24E-03 | Non-documented gene |
| *PGM2* | 2.85 | 1.64E-04 | 7.41E-03 | Non-documented gene |
| *SLC18A1* | 2.84 | 1.64E-04 | 7.41E-03 | Non-documented gene |
| *SLC38A5* | 2.83 | 1.67E-04 | 7.46E-03 | Non-documented gene |
| *EIF2S1* | 2.83 | 1.67E-04 | 7.46E-03 | Non-documented gene |
| *SKIV2L2* | 2.83 | 1.67E-04 | 7.46E-03 | Non-documented gene |
| *PEX14* | 2.81 | 1.68E-04 | 7.46E-03 | Reported gene |
| *TOM1* | 2.81 | 1.68E-04 | 7.46E-03 | Non-documented gene |
| *EXOD1* | 2.81 | 1.68E-04 | 7.46E-03 | Non-documented gene |
| *FTHL2* | 2.80 | 1.72E-04 | 7.56E-03 | Non-documented gene |
| *ZNF417* | 2.79 | 1.73E-04 | 7.56E-03 | Non-documented gene |
| *ABHD14B* | 2.79 | 1.73E-04 | 7.56E-03 | Non-documented gene |
| *ACTR6* | 2.78 | 1.73E-04 | 7.56E-03 | Non-documented gene |
| *TWSG1* | 2.78 | 1.75E-04 | 7.61E-03 | Non-documented gene |
| *ZNF490* | 2.77 | 1.76E-04 | 7.65E-03 | Non-documented gene |
| *PRPF39* | 2.75 | 1.83E-04 | 7.89E-03 | Non-documented gene |
| *ORC3L* | 2.73 | 1.89E-04 | 8.14E-03 | Non-documented gene |
| *WWP2* | 2.73 | 1.91E-04 | 8.15E-03 | Non-documented gene |
| *VIPR1* | 2.72 | 1.91E-04 | 8.15E-03 | Non-documented gene |
| *IARS2* | 2.72 | 1.92E-04 | 8.19E-03 | Non-documented gene |
| *NUPR1* | 2.71 | 1.94E-04 | 8.23E-03 | Non-documented gene |
| *TMEM16J* | 2.65 | 2.00E-04 | 8.41E-03 | Non-documented gene |
| *PGM1* | 2.65 | 2.00E-04 | 8.41E-03 | Non-documented gene |
| *MAPKAPK5* | 2.65 | 2.00E-04 | 8.41E-03 | Non-documented gene |
| *SENP7* | 2.64 | 2.02E-04 | 8.45E-03 | Non-documented gene |
| *PPP1R3D* | 2.62 | 2.06E-04 | 8.62E-03 | Non-documented gene |
| *RCSD1* | 2.62 | 2.08E-04 | 8.66E-03 | Non-documented gene |
| *CDK5R1* | 2.61 | 2.09E-04 | 8.67E-03 | Non-documented gene |
| *TNPO3* | 2.60 | 2.09E-04 | 8.67E-03 | Non-documented gene |
| *LY75* | 2.58 | 2.13E-04 | 8.74E-03 | Non-documented gene |
| *ADSL* | 2.58 | 2.13E-04 | 8.74E-03 | Non-documented gene |
| *SLFN13* | 2.56 | 2.14E-04 | 8.75E-03 | Non-documented gene |
| *NKX2-8* | 2.55 | 2.14E-04 | 8.75E-03 | Non-documented gene |
| *KRBA2* | 2.54 | 2.22E-04 | 8.95E-03 | Non-documented gene |
| *MAGOHB* | 2.53 | 2.22E-04 | 8.95E-03 | Non-documented gene |
| *NAT13* | 2.52 | 2.22E-04 | 8.95E-03 | Non-documented gene |
| *PTGFRN* | 2.52 | 2.22E-04 | 8.95E-03 | Non-documented gene |
| *RBM18* | 2.51 | 2.25E-04 | 9.02E-03 | Non-documented gene |
| *HNRNPL* | 2.50 | 2.25E-04 | 9.02E-03 | Non-documented gene |
| *HECTD1* | 2.49 | 2.31E-04 | 9.25E-03 | Non-documented gene |
| *KIT* | 2.47 | 2.33E-04 | 9.28E-03 | Non-documented gene |
| *METTL7A* | 2.46 | 2.36E-04 | 9.38E-03 | Non-documented gene |
| *NAT9* | 2.44 | 2.47E-04 | 9.78E-03 | Non-documented gene |
| *BSND* | 2.42 | 2.49E-04 | 9.81E-03 | Non-documented gene |
| *DOCK8* | 2.39 | 2.68E-04 | 1.05E-02 | Non-documented gene |
| *DDX26B* | 2.38 | 2.74E-04 | 1.07E-02 | Non-documented gene |
| *CDC42SE2* | 2.38 | 2.74E-04 | 1.07E-02 | Non-documented gene |
| *POLR2B* | 2.37 | 2.74E-04 | 1.07E-02 | Non-documented gene |
| *VENTXP7* | 2.37 | 2.77E-04 | 1.07E-02 | Non-documented gene |
| *GTF2IP1* | 2.36 | 2.77E-04 | 1.07E-02 | Non-documented gene |
| *FAM86A* | 2.36 | 2.77E-04 | 1.07E-02 | Non-documented gene |
| *KIF11* | 2.36 | 2.79E-04 | 1.07E-02 | Reported gene |
| *CDKL1* | 2.35 | 2.85E-04 | 1.08E-02 | Non-documented gene |
| *COIL* | 2.34 | 2.85E-04 | 1.08E-02 | Non-documented gene |
| *NHEDC1* | 2.34 | 2.85E-04 | 1.08E-02 | Non-documented gene |
| *GAGE4* | 2.33 | 2.85E-04 | 1.08E-02 | Non-documented gene |
| *SFT2D2* | 2.33 | 2.85E-04 | 1.08E-02 | Non-documented gene |
| *ZNF683* | 2.33 | 2.87E-04 | 1.08E-02 | Non-documented gene |
| *ADI1* | 2.32 | 2.91E-04 | 1.10E-02 | Non-documented gene |
| *PTMA* | 2.31 | 2.94E-04 | 1.11E-02 | Non-documented gene |
| *RABEP1* | 2.30 | 2.96E-04 | 1.11E-02 | Non-documented gene |
| *P2RX4* | 2.28 | 3.01E-04 | 1.12E-02 | Non-documented gene |
| *GOLGA2L1* | 2.27 | 3.04E-04 | 1.13E-02 | Non-documented gene |
| *FKSG24* | 2.26 | 3.07E-04 | 1.13E-02 | Non-documented gene |
| *GGTLC2* | 2.26 | 3.07E-04 | 1.13E-02 | Non-documented gene |
| *SPPL3* | 2.25 | 3.07E-04 | 1.13E-02 | Non-documented gene |
| *FAM3C* | 2.24 | 3.10E-04 | 1.14E-02 | Non-documented gene |
| *FOXRED2* | 2.22 | 3.12E-04 | 1.14E-02 | Non-documented gene |
| *SETD5* | 2.21 | 3.23E-04 | 1.18E-02 | Non-documented gene |
| *RHPN2* | 2.20 | 3.26E-04 | 1.19E-02 | Non-documented gene |
| *HBEGF* | 2.20 | 3.28E-04 | 1.19E-02 | Non-documented gene |
| *RPS3* | 2.18 | 3.37E-04 | 1.22E-02 | Non-documented gene |
| *ITK* | 2.18 | 3.40E-04 | 1.23E-02 | Non-documented gene |
| *OVCA2* | 2.16 | 3.48E-04 | 1.26E-02 | Non-documented gene |
| *SCLT1* | 2.13 | 3.54E-04 | 1.27E-02 | Non-documented gene |
| *CCL4L2* | 2.11 | 3.62E-04 | 1.30E-02 | Non-documented gene |
| *MS4A3* | 2.11 | 3.67E-04 | 1.31E-02 | Non-documented gene |
| *NUAK2* | 2.09 | 3.70E-04 | 1.31E-02 | Non-documented gene |
| *TREM1* | 2.08 | 3.70E-04 | 1.31E-02 | Non-documented gene |
| *PELI1* | 2.08 | 3.70E-04 | 1.31E-02 | Non-documented gene |
| *TMEM117* | 2.08 | 3.75E-04 | 1.33E-02 | Non-documented gene |
| *MT1E* | 2.06 | 3.81E-04 | 1.34E-02 | Non-documented gene |
| *THEM2* | 2.05 | 3.84E-04 | 1.35E-02 | Non-documented gene |
| *GPKOW* | 2.03 | 3.91E-04 | 1.37E-02 | Non-documented gene |
| *COPB1* | 2.02 | 3.98E-04 | 1.39E-02 | Non-documented gene |
| *P2RY5* | 2.01 | 3.98E-04 | 1.39E-02 | Non-documented gene |
| *ZNF593* | 2.00 | 4.02E-04 | 1.40E-02 | Non-documented gene |
| *FAM49B* | 2.00 | 4.05E-04 | 1.40E-02 | Non-documented gene |
| *TIMP2* | 2.00 | 4.06E-04 | 1.40E-02 | Non-documented gene |
| *ARIH2* | 1.99 | 4.06E-04 | 1.40E-02 | Non-documented gene |
| *ZNF236* | 1.99 | 4.06E-04 | 1.40E-02 | Non-documented gene |
| *TXNL1* | 1.97 | 4.19E-04 | 1.44E-02 | Non-documented gene |
| *SLBP* | 1.97 | 4.25E-04 | 1.45E-02 | Non-documented gene |
| *PPP1R13B* | 1.96 | 4.25E-04 | 1.45E-02 | Non-documented gene |
| *PCNXL2* | 1.95 | 4.38E-04 | 1.49E-02 | Non-documented gene |
| *NUDCD2* | 1.94 | 4.39E-04 | 1.49E-02 | Non-documented gene |
| *SH3BGRL3* | 1.93 | 4.46E-04 | 1.51E-02 | Non-documented gene |
| *RPS28* | 1.93 | 4.49E-04 | 1.51E-02 | Non-documented gene |
| *GIMAP1* | 1.92 | 4.50E-04 | 1.51E-02 | Non-documented gene |
| *ETFDH* | 1.92 | 4.50E-04 | 1.51E-02 | Non-documented gene |
| *CD163* | 1.92 | 4.54E-04 | 1.52E-02 | Non-documented gene |
| *ANTXR2* | 1.92 | 4.57E-04 | 1.52E-02 | Non-documented gene |
| *PPP2R3C* | 1.91 | 4.57E-04 | 1.52E-02 | Non-documented gene |
| *LCMT1* | 1.91 | 4.60E-04 | 1.53E-02 | Non-documented gene |
| *POP1* | 1.91 | 4.65E-04 | 1.54E-02 | Non-documented gene |
| *KIF1B* | 1.90 | 4.66E-04 | 1.54E-02 | Non-documented gene |
| *LARP7* | 1.90 | 4.69E-04 | 1.55E-02 | Non-documented gene |
| *PHTF1* | 1.89 | 4.76E-04 | 1.56E-02 | Non-documented gene |
| *PPP1R8* | 1.87 | 4.88E-04 | 1.59E-02 | Non-documented gene |
| *RNASEH2B* | 1.87 | 4.88E-04 | 1.59E-02 | Non-documented gene |
| *LYCAT* | 1.87 | 4.88E-04 | 1.59E-02 | Non-documented gene |
| *HCCS* | 1.87 | 4.90E-04 | 1.59E-02 | Non-documented gene |
| *LPGAT1* | 1.87 | 4.91E-04 | 1.60E-02 | Non-documented gene |
| *ZNF99* | 1.86 | 4.94E-04 | 1.60E-02 | Non-documented gene |
| *STX16* | 1.85 | 4.98E-04 | 1.61E-02 | Non-documented gene |
| *CASP2* | 1.83 | 5.09E-04 | 1.64E-02 | Non-documented gene |
| *LAPTM4A* | 1.82 | 5.13E-04 | 1.65E-02 | Non-documented gene |
| *COL23A1* | 1.82 | 5.16E-04 | 1.65E-02 | Non-documented gene |
| *PFDN4* | 1.80 | 5.23E-04 | 1.65E-02 | Non-documented gene |
| *MYEOV* | 1.80 | 5.23E-04 | 1.65E-02 | Non-documented gene |
| *KSR2* | 1.80 | 5.23E-04 | 1.65E-02 | Non-documented gene |
| *SLC25A12* | 1.79 | 5.23E-04 | 1.65E-02 | Non-documented gene |
| *NUS1* | 1.79 | 5.24E-04 | 1.65E-02 | Non-documented gene |
| *DMXL1* | 1.79 | 5.26E-04 | 1.65E-02 | Non-documented gene |
| *MTMR4* | 1.78 | 5.26E-04 | 1.65E-02 | Non-documented gene |
| *BRWD2* | 1.78 | 5.26E-04 | 1.65E-02 | Non-documented gene |
| *APITD1* | 1.76 | 5.35E-04 | 1.68E-02 | Non-documented gene |
| *KIR3DL2* | 1.75 | 5.39E-04 | 1.68E-02 | Non-documented gene |
| *CLIC3* | 1.75 | 5.43E-04 | 1.69E-02 | Non-documented gene |
| *PXMP3* | 1.74 | 5.51E-04 | 1.71E-02 | Non-documented gene |
| *PTGDR* | 1.74 | 5.53E-04 | 1.71E-02 | Non-documented gene |
| *PHEX* | 1.73 | 5.53E-04 | 1.71E-02 | Non-documented gene |
| *NDUFAB1* | 1.73 | 5.54E-04 | 1.71E-02 | Non-documented gene |
| *FTHL3* | 1.72 | 5.65E-04 | 1.74E-02 | Non-documented gene |
| *ZNF544* | 1.71 | 5.68E-04 | 1.75E-02 | Non-documented gene |
| *PEX1* | 1.70 | 5.76E-04 | 1.76E-02 | Non-documented gene |
| *GLE1* | 1.69 | 5.76E-04 | 1.76E-02 | Non-documented gene |
| *TEDDM1* | 1.68 | 5.87E-04 | 1.79E-02 | Non-documented gene |
| *FAM54A* | 1.68 | 5.87E-04 | 1.79E-02 | Non-documented gene |
| *RPL10A* | 1.67 | 5.97E-04 | 1.81E-02 | Non-documented gene |
| *ERG* | 1.66 | 5.97E-04 | 1.81E-02 | Non-documented gene |
| *EARS2* | 1.65 | 6.13E-04 | 1.85E-02 | Non-documented gene |
| *RNGTT* | 1.63 | 6.25E-04 | 1.88E-02 | Non-documented gene |
| *SPDYC* | 1.61 | 6.36E-04 | 1.91E-02 | Non-documented gene |
| *FTHL11* | 1.61 | 6.41E-04 | 1.92E-02 | Non-documented gene |
| *CEP63* | 1.61 | 6.41E-04 | 1.92E-02 | Non-documented gene |
| *ZNF484* | 1.59 | 6.55E-04 | 1.96E-02 | Non-documented gene |
| *OBSCN* | 1.58 | 6.63E-04 | 1.97E-02 | Non-documented gene |
| *MUC4* | 1.57 | 6.63E-04 | 1.97E-02 | Non-documented gene |
| *MAGEB3* | 1.56 | 6.69E-04 | 1.98E-02 | Non-documented gene |
| *CPT1B* | 1.56 | 6.74E-04 | 1.99E-02 | Non-documented gene |
| *RAPGEFL1* | 1.55 | 6.74E-04 | 1.99E-02 | Non-documented gene |
| *ANKRD40* | 1.54 | 6.76E-04 | 1.99E-02 | Non-documented gene |
| *FTHL8* | 1.53 | 6.83E-04 | 2.01E-02 | Non-documented gene |
| *RPS6KA2* | 1.53 | 6.88E-04 | 2.02E-02 | Non-documented gene |
| *TAAR1* | 1.52 | 6.91E-04 | 2.02E-02 | Non-documented gene |
| *TOP2B* | 1.52 | 6.96E-04 | 2.03E-02 | Non-documented gene |
| *NOS3* | 1.51 | 7.01E-04 | 2.04E-02 | Non-documented gene |
| *PLD4* | 1.51 | 7.05E-04 | 2.05E-02 | Non-documented gene |
| *ALS2CR16* | 1.49 | 7.10E-04 | 2.06E-02 | Non-documented gene |
| *EDG8* | 1.49 | 7.12E-04 | 2.06E-02 | Non-documented gene |
| *IL23A* | 1.49 | 7.15E-04 | 2.06E-02 | Non-documented gene |
| *FAM120B* | 1.48 | 7.24E-04 | 2.08E-02 | Non-documented gene |
| *AKTIP* | 1.47 | 7.31E-04 | 2.10E-02 | Non-documented gene |
| *FRMD3* | 1.45 | 7.48E-04 | 2.14E-02 | Non-documented gene |
| *LIME1* | 1.44 | 7.54E-04 | 2.16E-02 | Non-documented gene |
| *ZNF138* | 1.43 | 7.65E-04 | 2.18E-02 | Non-documented gene |
| *SRF* | 1.43 | 7.65E-04 | 2.18E-02 | Non-documented gene |
| *IER3IP1* | 1.42 | 7.72E-04 | 2.19E-02 | Non-documented gene |
| *CD52* | 1.42 | 7.75E-04 | 2.19E-02 | Non-documented gene |
| *SLC45A4* | 1.41 | 7.87E-04 | 2.23E-02 | Non-documented gene |
| *HSD17B4* | 1.39 | 7.98E-04 | 2.25E-02 | Non-documented gene |
| *POLR3C* | 1.39 | 8.06E-04 | 2.27E-02 | Non-documented gene |
| *CHES1* | 1.37 | 8.28E-04 | 2.33E-02 | Non-documented gene |
| *TEAD4* | 1.36 | 8.36E-04 | 2.34E-02 | Non-documented gene |
| *SBDS* | 1.34 | 8.44E-04 | 2.36E-02 | Non-documented gene |
| *CD7* | 1.33 | 8.46E-04 | 2.36E-02 | Non-documented gene |
| *FKBP3* | 1.32 | 8.49E-04 | 2.36E-02 | Non-documented gene |
| *STAP2* | 1.31 | 8.53E-04 | 2.37E-02 | Non-documented gene |
| *RNPC3* | 1.29 | 8.69E-04 | 2.41E-02 | Non-documented gene |
| *EIF3H* | 1.27 | 8.91E-04 | 2.46E-02 | Non-documented gene |
| *PPP1R9B* | 1.24 | 9.26E-04 | 2.55E-02 | Non-documented gene |
| *CYP4F3* | 1.23 | 9.42E-04 | 2.59E-02 | Non-documented gene |
| *CPSF6* | 1.22 | 9.45E-04 | 2.60E-02 | Non-documented gene |
| *HYAL3* | 1.20 | 9.70E-04 | 2.66E-02 | Non-documented gene |
| *SNORA65* | 1.20 | 9.78E-04 | 2.67E-02 | Non-documented gene |
| *TACR3* | 1.19 | 9.86E-04 | 2.69E-02 | Non-documented gene |
| *SLC6A13* | 1.17 | 1.00E-03 | 2.73E-02 | Non-documented gene |
| *RSRC2* | 1.17 | 1.01E-03 | 2.74E-02 | Non-documented gene |
| *HSD17B11* | 1.16 | 1.01E-03 | 2.74E-02 | Non-documented gene |
| *PLCL1* | 1.16 | 1.01E-03 | 2.74E-02 | Non-documented gene |
| *HNRPH3* | 1.15 | 1.03E-03 | 2.78E-02 | Non-documented gene |
| *MRPS24* | 1.14 | 1.04E-03 | 2.79E-02 | Non-documented gene |
| *KIR3DL1* | 1.14 | 1.04E-03 | 2.81E-02 | Non-documented gene |
| *RASSF4* | 1.10 | 1.10E-03 | 2.94E-02 | Non-documented gene |
| *STAT4* | 1.08 | 1.11E-03 | 2.97E-02 | Non-documented gene |
| *RAVER2* | 1.08 | 1.12E-03 | 2.98E-02 | Non-documented gene |
| *SNORD55* | 1.07 | 1.12E-03 | 2.98E-02 | Non-documented gene |
| *ZNF570* | 1.07 | 1.12E-03 | 2.98E-02 | Non-documented gene |
| *UBE2Q1* | 1.07 | 1.13E-03 | 2.99E-02 | Non-documented gene |
| *SNAI3* | 1.06 | 1.14E-03 | 3.02E-02 | Non-documented gene |
| *ACOT7* | 1.06 | 1.15E-03 | 3.03E-02 | Non-documented gene |
| *RPS6KA1* | 1.04 | 1.17E-03 | 3.08E-02 | Non-documented gene |
| *ENOPH1* | 1.02 | 1.19E-03 | 3.12E-02 | Non-documented gene |
| *CCBL1* | 1.02 | 1.19E-03 | 3.12E-02 | Non-documented gene |
| *IL18RAP* | 1.01 | 1.19E-03 | 3.13E-02 | Non-documented gene |
| *KCNQ2* | 0.97 | 1.25E-03 | 3.27E-02 | Non-documented gene |
| *SF3B5* | 0.96 | 1.26E-03 | 3.30E-02 | Non-documented gene |
| *GNLY* | 0.94 | 1.28E-03 | 3.33E-02 | Non-documented gene |
| *TMEM161A* | 0.93 | 1.29E-03 | 3.35E-02 | Non-documented gene |
| *YY1AP1* | 0.91 | 1.31E-03 | 3.40E-02 | Non-documented gene |
| *SNORD68* | 0.89 | 1.33E-03 | 3.44E-02 | Non-documented gene |
| *KCNK6* | 0.89 | 1.33E-03 | 3.44E-02 | Non-documented gene |
| *RPS6KB1* | 0.89 | 1.34E-03 | 3.45E-02 | Non-documented gene |
| *E2F4* | 0.88 | 1.34E-03 | 3.45E-02 | Non-documented gene |
| *SUMO1P1* | 0.88 | 1.34E-03 | 3.45E-02 | Non-documented gene |
| *ERAP1* | 0.87 | 1.36E-03 | 3.49E-02 | Non-documented gene |
| *SEC24C* | 0.86 | 1.37E-03 | 3.50E-02 | Non-documented gene |
| *GFI1B* | 0.86 | 1.38E-03 | 3.52E-02 | Non-documented gene |
| *BDP1* | 0.86 | 1.38E-03 | 3.52E-02 | Non-documented gene |
| *MOAP1* | 0.84 | 1.41E-03 | 3.58E-02 | Non-documented gene |
| *TMEM115* | 0.83 | 1.42E-03 | 3.60E-02 | Non-documented gene |
| *ELMO3* | 0.82 | 1.44E-03 | 3.64E-02 | Non-documented gene |
| *CRABP2* | 0.82 | 1.44E-03 | 3.65E-02 | Non-documented gene |
| *GEMIN7* | 0.81 | 1.45E-03 | 3.66E-02 | Non-documented gene |
| *EXOSC4* | 0.81 | 1.46E-03 | 3.66E-02 | Non-documented gene |
| *MED4* | 0.80 | 1.46E-03 | 3.66E-02 | Non-documented gene |
| *NEDD8* | 0.80 | 1.47E-03 | 3.66E-02 | Non-documented gene |
| *SP3* | 0.80 | 1.47E-03 | 3.66E-02 | Non-documented gene |
| *ADAM15* | 0.80 | 1.47E-03 | 3.66E-02 | Non-documented gene |
| *ALS2CR13* | 0.80 | 1.47E-03 | 3.66E-02 | Non-documented gene |
| *CDC42BPB* | 0.76 | 1.51E-03 | 3.76E-02 | Non-documented gene |
| *PRF1* | 0.75 | 1.54E-03 | 3.82E-02 | Non-documented gene |
| *ZNF154* | 0.73 | 1.56E-03 | 3.87E-02 | Non-documented gene |
| *HS2ST1* | 0.72 | 1.58E-03 | 3.91E-02 | Non-documented gene |
| *TFDP2* | 0.71 | 1.60E-03 | 3.96E-02 | Non-documented gene |
| *WFS1* | 0.68 | 1.64E-03 | 4.03E-02 | Non-documented gene |
| *CLDN23* | 0.68 | 1.65E-03 | 4.06E-02 | Non-documented gene |
| *TNIP2* | 0.67 | 1.68E-03 | 4.12E-02 | Non-documented gene |
| *ATF2* | 0.66 | 1.68E-03 | 4.12E-02 | Non-documented gene |
| *LRRC29* | 0.66 | 1.69E-03 | 4.13E-02 | Non-documented gene |
| *PLP2* | 0.65 | 1.70E-03 | 4.15E-02 | Non-documented gene |
| *GPR89C* | 0.65 | 1.70E-03 | 4.15E-02 | Non-documented gene |
| *IL2RB* | 0.64 | 1.73E-03 | 4.19E-02 | Reported gene |
| *FCHO1* | 0.64 | 1.73E-03 | 4.20E-02 | Non-documented gene |
| *SFRS11* | 0.63 | 1.74E-03 | 4.21E-02 | Non-documented gene |
| *NUBP2* | 0.63 | 1.74E-03 | 4.21E-02 | Non-documented gene |
| *ZNF121* | 0.62 | 1.76E-03 | 4.25E-02 | Non-documented gene |
| *GPR156* | 0.61 | 1.78E-03 | 4.28E-02 | Non-documented gene |
| *HLA-DOA* | 0.61 | 1.79E-03 | 4.30E-02 | Reported gene |
| *ACCN1* | 0.60 | 1.79E-03 | 4.30E-02 | Non-documented gene |
| *GPR18* | 0.59 | 1.82E-03 | 4.35E-02 | Non-documented gene |
| *MOBK1B* | 0.58 | 1.83E-03 | 4.36E-02 | Non-documented gene |
| *TNNT2* | 0.58 | 1.83E-03 | 4.36E-02 | Non-documented gene |
| *OR12D2* | 0.58 | 1.84E-03 | 4.36E-02 | Non-documented gene |
| *FOXC2* | 0.58 | 1.84E-03 | 4.36E-02 | Non-documented gene |
| *LASP1* | 0.58 | 1.84E-03 | 4.36E-02 | Non-documented gene |
| *EMX2* | 0.57 | 1.85E-03 | 4.37E-02 | Non-documented gene |
| *MALAT1* | 0.57 | 1.85E-03 | 4.37E-02 | Non-documented gene |
| *HIST1H2AM* | 0.56 | 1.88E-03 | 4.42E-02 | Non-documented gene |
| *EVC* | 0.56 | 1.88E-03 | 4.42E-02 | Non-documented gene |
| *NUSAP1* | 0.56 | 1.88E-03 | 4.42E-02 | Non-documented gene |
| *ARL3* | 0.55 | 1.91E-03 | 4.48E-02 | Non-documented gene |
| *BBS12* | 0.54 | 1.92E-03 | 4.49E-02 | Non-documented gene |
| *PLEKHN1* | 0.53 | 1.93E-03 | 4.51E-02 | Non-documented gene |
| *HSPA4* | 0.52 | 1.94E-03 | 4.52E-02 | Non-documented gene |
| *ANXA4* | 0.51 | 1.96E-03 | 4.55E-02 | Non-documented gene |
| *FEM1C* | 0.51 | 1.96E-03 | 4.55E-02 | Non-documented gene |
| *MARS2* | 0.51 | 1.96E-03 | 4.55E-02 | Non-documented gene |
| *SMAD7* | 0.50 | 1.99E-03 | 4.59E-02 | Non-documented gene |
| *SLC9A3* | 0.50 | 1.99E-03 | 4.59E-02 | Non-documented gene |
| *PMFBP1* | 0.50 | 1.99E-03 | 4.59E-02 | Non-documented gene |
| *PDZD4* | 0.50 | 2.00E-03 | 4.59E-02 | Non-documented gene |
| *MFN2* | 0.50 | 2.00E-03 | 4.59E-02 | Non-documented gene |
| *SPERT* | 0.50 | 2.00E-03 | 4.59E-02 | Non-documented gene |
| *SNRP70* | 0.49 | 2.01E-03 | 4.60E-02 | Non-documented gene |
| *ELA1* | 0.48 | 2.06E-03 | 4.71E-02 | Non-documented gene |
| *SMN2* | 0.47 | 2.09E-03 | 4.75E-02 | Non-documented gene |
| *ZBTB25* | 0.47 | 2.09E-03 | 4.75E-02 | Non-documented gene |
| *OSGIN1* | 0.47 | 2.09E-03 | 4.75E-02 | Non-documented gene |
| *ARRDC1* | 0.44 | 2.15E-03 | 4.88E-02 | Reported gene |
| *KLRD1* | 0.43 | 2.19E-03 | 4.97E-02 | Non-documented gene |
| *CSAD* | 0.43 | 2.20E-03 | 4.98E-02 | Non-documented gene |

**Supplemental Table S2. 83 Sherlock-identified genes from discovery Dataset #2 overlapped with MAGMA-identified genes**

| **Gene Name** | **LBF** | **Sherlock-based P-value** | **FDR-value** | **MAGMA-based P-value** | **Bonferroni adjusted P- value** | **GWAS Catalog** |
| --- | --- | --- | --- | --- | --- | --- |
| *HLA-DQA1* | 12.91 | 7.87E-07 | 2.05E-04 | 1.76E-68 | 3.38E-64 | Reported gene |
| *NOTCH4* | 12.37 | 7.87E-07 | 2.05E-04 | 1.87E-22 | 3.60E-18 | Reported gene |
| *PSMB9* | 12.17 | 7.87E-07 | 2.05E-04 | 1.08E-09 | 2.09E-05 | Non-documented gene |
| *HLA-DRB5* | 11.52 | 7.87E-07 | 2.05E-04 | 9.80E-17 | 1.88E-12 | Reported gene |
| *HLA-DPB1* | 11.26 | 7.87E-07 | 2.05E-04 | 5.12E-11 | 9.85E-07 | Non-documented gene |
| *HLA-DRB1* | 10.91 | 7.87E-07 | 2.05E-04 | 1.47E-24 | 2.83E-20 | Reported gene |
| *HLA-DMA* | 8.79 | 7.87E-07 | 2.05E-04 | 1.30E-09 | 2.51E-05 | Non-documented gene |
| *TAP2* | 8.70 | 7.87E-07 | 2.05E-04 | 1.07E-33 | 2.06E-29 | Non-documented gene |
| *ADORA1* | 7.79 | 7.87E-07 | 2.05E-04 | 7.37E-10 | 1.42E-05 | Reported gene |
| *APOM* | 7.49 | 7.87E-07 | 2.05E-04 | 2.31E-10 | 4.44E-06 | Non-documented gene |
| *TLR6* | 7.38 | 7.87E-07 | 2.05E-04 | 1.83E-26 | 3.51E-22 | Reported gene |
| *PMM1* | 7.36 | 7.87E-07 | 2.05E-04 | 5.24E-09 | 1.01E-04 | Non-documented gene |
| *IL18R1* | 7.22 | 7.87E-07 | 2.05E-04 | 1.36E-48 | 2.61E-44 | Reported gene |
| *MPHOSPH9* | 7.20 | 7.87E-07 | 2.05E-04 | 3.51E-09 | 6.75E-05 | Reported gene |
| *SUOX* | 7.11 | 7.87E-07 | 2.05E-04 | 9.23E-13 | 1.78E-08 | Reported gene |
| *IKZF3* | 7.02 | 7.87E-07 | 2.05E-04 | 3.30E-15 | 6.35E-11 | Reported gene |
| *ZNF76* | 6.96 | 7.87E-07 | 2.05E-04 | 1.36E-08 | 2.62E-04 | Non-documented gene |
| *BCL6* | 6.95 | 7.87E-07 | 2.05E-04 | 7.35E-11 | 1.41E-06 | Non-documented gene |
| *PSMD3* | 6.83 | 1.57E-06 | 2.05E-04 | 6.49E-42 | 1.25E-37 | Non-documented gene |
| *STARD3* | 6.81 | 1.57E-06 | 2.05E-04 | 1.18E-45 | 2.26E-41 | Non-documented gene |
| *MSH5* | 6.80 | 1.57E-06 | 2.05E-04 | 2.54E-13 | 4.89E-09 | Non-documented gene |
| *MEI1* | 6.70 | 1.57E-06 | 2.05E-04 | 6.16E-09 | 1.19E-04 | Non-documented gene |
| *HLA-DOB* | 6.67 | 1.57E-06 | 2.05E-04 | 6.25E-32 | 1.20E-27 | Non-documented gene |
| *NSF* | 6.66 | 1.57E-06 | 2.05E-04 | 1.46E-07 | 2.80E-03 | Non-documented gene |
| *TNFSF4* | 6.60 | 1.57E-06 | 2.05E-04 | 4.08E-14 | 7.84E-10 | Reported gene |
| *ME2* | 6.60 | 1.57E-06 | 2.05E-04 | 1.26E-09 | 2.42E-05 | Non-documented gene |
| *PHF5A* | 6.56 | 1.57E-06 | 2.05E-04 | 3.73E-10 | 7.18E-06 | Reported gene |
| *ACTR1A* | 6.55 | 1.57E-06 | 2.05E-04 | 3.42E-09 | 6.58E-05 | Non-documented gene |
| *DEXI* | 6.42 | 1.57E-06 | 2.05E-04 | 2.32E-13 | 4.45E-09 | Non-documented gene |
| *RERE* | 6.40 | 1.57E-06 | 2.05E-04 | 5.70E-12 | 1.10E-07 | Reported gene |
| *D2HGDH* | 6.34 | 1.57E-06 | 2.05E-04 | 5.42E-40 | 1.04E-35 | Reported gene |
| *RUVBL1* | 6.30 | 1.57E-06 | 2.05E-04 | 9.73E-09 | 1.87E-04 | Non-documented gene |
| *MUS81* | 6.26 | 1.57E-06 | 2.05E-04 | 1.81E-07 | 3.48E-03 | Non-documented gene |
| *BRD2* | 6.25 | 1.57E-06 | 2.05E-04 | 9.31E-13 | 1.79E-08 | Reported gene |
| *NSMCE1* | 6.22 | 1.57E-06 | 2.05E-04 | 6.37E-12 | 1.22E-07 | Non-documented gene |
| *JAZF1* | 6.18 | 1.57E-06 | 2.05E-04 | 2.67E-09 | 5.13E-05 | Reported gene |
| *TDRKH* | 6.18 | 1.57E-06 | 2.05E-04 | 5.17E-11 | 9.95E-07 | Reported gene |
| *IL4R* | 6.15 | 1.57E-06 | 2.05E-04 | 2.62E-19 | 5.03E-15 | Reported gene |
| *SLC22A4* | 6.13 | 1.57E-06 | 2.05E-04 | 1.84E-07 | 3.54E-03 | Non-documented gene |
| *STAT6* | 6.08 | 1.57E-06 | 2.05E-04 | 1.63E-24 | 3.14E-20 | Reported gene |
| *SMARCE1* | 6.06 | 1.57E-06 | 2.05E-04 | 4.18E-10 | 8.05E-06 | Reported gene |
| *DEF6* | 6.03 | 1.57E-06 | 2.05E-04 | 6.32E-07 | 1.21E-02 | Non-documented gene |
| *GLB1* | 6.01 | 1.57E-06 | 2.05E-04 | 4.82E-13 | 9.27E-09 | Reported gene |
| *AHI1* | 5.89 | 1.57E-06 | 2.05E-04 | 2.10E-06 | 4.03E-02 | Non-documented gene |
| *CSNK2B* | 5.81 | 1.57E-06 | 2.05E-04 | 1.06E-09 | 2.05E-05 | Non-documented gene |
| *FCER1G* | 5.79 | 1.57E-06 | 2.05E-04 | 3.55E-09 | 6.83E-05 | Reported gene |
| *SLC15A2* | 5.77 | 1.57E-06 | 2.05E-04 | 3.32E-09 | 6.39E-05 | Non-documented gene |
| *IRF1* | 5.72 | 1.57E-06 | 2.05E-04 | 3.00E-12 | 5.77E-08 | Non-documented gene |
| *RAD50* | 5.60 | 1.57E-06 | 2.05E-04 | 8.32E-25 | 1.60E-20 | Reported gene |
| *HSPA1B* | 5.54 | 1.57E-06 | 2.05E-04 | 3.64E-13 | 7.01E-09 | Non-documented gene |
| *HLA-B* | 5.50 | 1.57E-06 | 2.05E-04 | 5.61E-29 | 1.08E-24 | Reported gene |
| *LST1* | 5.14 | 6.30E-06 | 7.39E-04 | 1.31E-15 | 2.52E-11 | Non-documented gene |
| *POLI* | 5.12 | 6.30E-06 | 7.39E-04 | 9.06E-12 | 1.74E-07 | Reported gene |
| *MICA* | 5.03 | 6.30E-06 | 7.39E-04 | 3.39E-22 | 6.51E-18 | Non-documented gene |
| *ITPR3* | 4.81 | 7.87E-06 | 8.10E-04 | 1.94E-11 | 3.74E-07 | Non-documented gene |
| *PHF19* | 4.81 | 7.87E-06 | 8.10E-04 | 7.02E-09 | 1.35E-04 | Non-documented gene |
| *HSPA1A* | 4.80 | 7.87E-06 | 8.10E-04 | 4.66E-13 | 8.97E-09 | Non-documented gene |
| *SLC22A5* | 4.54 | 1.42E-05 | 1.33E-03 | 1.77E-07 | 3.41E-03 | Reported gene |
| *ZC3H10* | 4.29 | 1.89E-05 | 1.64E-03 | 7.11E-07 | 1.37E-02 | Non-documented gene |
| *TRAF1* | 4.26 | 2.05E-05 | 1.75E-03 | 5.35E-08 | 1.03E-03 | Non-documented gene |
| *CCDC66* | 4.22 | 2.05E-05 | 1.75E-03 | 5.43E-07 | 1.04E-02 | Non-documented gene |
| *CTSW* | 4.15 | 2.52E-05 | 2.06E-03 | 1.08E-07 | 2.08E-03 | Non-documented gene |
| *EFEMP2* | 4.12 | 2.83E-05 | 2.24E-03 | 6.20E-07 | 1.19E-02 | Non-documented gene |
| *FAM3A* | 4.08 | 3.15E-05 | 2.42E-03 | 5.84E-08 | 1.12E-03 | Non-documented gene |
| *POU2F1* | 3.99 | 3.78E-05 | 2.70E-03 | 6.64E-08 | 1.28E-03 | Non-documented gene |
| *HEXIM2* | 3.94 | 4.25E-05 | 2.99E-03 | 6.62E-10 | 1.27E-05 | Non-documented gene |
| *NDFIP1* | 3.93 | 4.25E-05 | 2.99E-03 | 4.41E-13 | 8.49E-09 | Reported gene |
| *HLA-C* | 3.71 | 5.83E-05 | 3.73E-03 | 1.32E-15 | 2.54E-11 | Non-documented gene |
| *HLA-A* | 3.07 | 1.29E-04 | 6.34E-03 | 2.79E-09 | 5.37E-05 | Non-documented gene |
| *MICB* | 3.02 | 1.34E-04 | 6.47E-03 | 9.39E-12 | 1.81E-07 | Reported gene |
| *KIF11* | 2.36 | 2.79E-04 | 1.07E-02 | 1.78E-06 | 3.43E-02 | Reported gene |
| *SPPL3* | 2.25 | 3.07E-04 | 1.13E-02 | 1.14E-17 | 2.20E-13 | Non-documented gene |
| *RAPGEFL1* | 1.55 | 6.74E-04 | 1.99E-02 | 4.18E-08 | 8.04E-04 | Non-documented gene |
| *LIME1* | 1.44 | 7.54E-04 | 2.16E-02 | 8.82E-08 | 1.70E-03 | Non-documented gene |
| *CD52* | 1.42 | 7.75E-04 | 2.19E-02 | 2.04E-06 | 3.93E-02 | Non-documented gene |
| *PLCL1* | 1.16 | 1.01E-03 | 2.74E-02 | 6.95E-07 | 1.34E-02 | Non-documented gene |
| *IL18RAP* | 1.01 | 1.19E-03 | 3.13E-02 | 2.01E-29 | 3.87E-25 | Non-documented gene |
| *YY1AP1* | 0.91 | 1.31E-03 | 3.40E-02 | 2.10E-06 | 4.04E-02 | Non-documented gene |
| *HLA-DOA* | 0.61 | 1.79E-03 | 4.30E-02 | 1.90E-15 | 3.65E-11 | Reported gene |
| *GPR18* | 0.59 | 1.82E-03 | 4.35E-02 | 5.04E-14 | 9.69E-10 | Non-documented gene |
| *NUSAP1* | 0.56 | 1.88E-03 | 4.42E-02 | 4.45E-09 | 8.56E-05 | Non-documented gene |
| *ARL3* | 0.55 | 1.91E-03 | 4.48E-02 | 3.07E-08 | 5.90E-04 | Non-documented gene |
| *SMAD7* | 0.50 | 1.99E-03 | 4.59E-02 | 1.16E-06 | 2.24E-02 | Non-documented gene |

**Supplemental Table S3. Significant KEGG pathways enriched by childhood-onset asthma-relevant genes (N = 83) identified from Sherlock Bayesian analysis in the discovery dataset and MAGMA-based analysis**

| **KEGG-ID** | **KEGG pathways** | **Gene number** | **Gene proportion** | **Fold Enrichment** | **P-value** | **FDR** |
| --- | --- | --- | --- | --- | --- | --- |
| hsa04612 | Antigen processing and presentation | 13 | 0.12 | 26.74 | 1.57E-14 | 1.77E-11 |
| hsa05332 | Graft-versus-host disease | 10 | 0.09 | 47.38 | 2.07E-13 | 2.34E-10 |
| hsa05330 | Allograft rejection | 10 | 0.09 | 42.25 | 6.55E-13 | 7.41E-10 |
| hsa04940 | Type I diabetes mellitus | 10 | 0.09 | 37.22 | 2.29E-12 | 2.60E-09 |
| hsa05321 | Inflammatory bowel disease (IBD) | 11 | 0.10 | 26.87 | 3.53E-12 | 3.99E-09 |
| hsa05320 | Autoimmune thyroid disease | 10 | 0.09 | 30.07 | 1.81E-11 | 2.05E-08 |
| hsa05416 | Viral myocarditis | 10 | 0.09 | 27.43 | 4.33E-11 | 4.90E-08 |
| hsa05310 | Asthma | 8 | 0.07 | 41.69 | 4.09E-10 | 4.63E-07 |
| hsa05168 | Herpes simplex infection | 13 | 0.12 | 11.11 | 6.55E-10 | 7.41E-07 |
| hsa04145 | Phagosome | 12 | 0.11 | 12.51 | 1.16E-09 | 1.31E-06 |
| hsa04514 | Cell adhesion molecules (CAMs) | 10 | 0.09 | 11.01 | 1.64E-07 | 1.86E-04 |
| hsa05145 | Toxoplasmosis | 9 | 0.08 | 12.79 | 3.01E-07 | 3.41E-04 |
| hsa04672 | Intestinal immune network for IgA production | 7 | 0.06 | 23.28 | 3.69E-07 | 4.17E-04 |
| hsa05150 | Staphylococcus aureus infection | 7 | 0.06 | 20.27 | 8.59E-07 | 9.72E-04 |
| hsa05140 | Leishmaniasis | 7 | 0.06 | 15.41 | 4.40E-06 | 4.98E-03 |
| hsa05169 | Epstein-Barr virus infection | 8 | 0.07 | 10.25 | 8.81E-06 | 9.97E-03 |
| hsa05164 | Influenza A | 9 | 0.08 | 8.09 | 9.71E-06 | 1.10E-02 |
| hsa05152 | Tuberculosis | 9 | 0.08 | 7.95 | 1.10E-05 | 1.25E-02 |
| hsa05323 | Rheumatoid arthritis | 7 | 0.06 | 12.44 | 1.54E-05 | 1.74E-02 |
| hsa05166 | HTLV-I infection | 10 | 0.09 | 6.16 | 2.08E-05 | 2.35E-02 |

**Supplemental Table S4. Significant GO-terms of molecular function enriched by childhood-onset asthma-relevant genes (N = 83) identified from Sherlock Bayesian analysis in the discovery dataset and MAGMA-based analysis**

| **GO-ID** | **GO terms** | **Gene number** | **Gene proportion** | **Fold Enrichment** | **P-value** | **FDR** |
| --- | --- | --- | --- | --- | --- | --- |
| GO:0032395 | MHC class II receptor activity | 7 | 0.06 | 105.04 | 2.80E-11 | 3.48E-08 |
| GO:0042605 | Peptide antigen binding | 7 | 0.06 | 56.27 | 2.01E-09 | 2.51E-06 |
| GO:0023026 | MHC class II protein complex binding | 4 | 0.04 | 56.27 | 4.35E-05 | 5.41E-02 |
| GO:0046977 | TAP binding | 3 | 0.03 | 225.08 | 5.67E-05 | 7.06E-02 |
| GO:0042623 | ATPase activity, coupled | 3 | 0.03 | 56.27 | 1.22E-03 | 1 |
| GO:0016887 | ATPase activity | 5 | 0.05 | 6.15 | 8.53E-03 | 1 |
| GO:0015491 | Cation-cation antiporter activity | 2 | 0.02 | 225.08 | 8.75E-03 | 1 |
| GO:0005515 | Protein binding | 50 | 0.46 | 1.28 | 9.42E-03 | 1 |
| GO:0003823 | Antigen binding | 4 | 0.04 | 8.74 | 1.05E-02 | 1 |
| GO:0015226 | Carnitine transmembrane transporter activity | 2 | 0.02 | 150.05 | 1.31E-02 | 1 |

**Supplemental Table S5. Significant GO-terms of cellular component enriched by childhood-onset asthma-relevant genes (N = 83) identified from Sherlock Bayesian analysis in the discovery dataset and MAGMA-based analysis**

| **GO-ID** | **GO terms** | **Gene number** | **Gene proportion** | **Fold Enrichment** | **P-value** | **FDR** |
| --- | --- | --- | --- | --- | --- | --- |
| GO:0042613 | MHC class II protein complex | 9 | 0.08 | 93.19 | 2.63E-14 | 3.16E-11 |
| GO:0071556 | Integral component of lumenal side of endoplasmic reticulum membrane | 8 | 0.07 | 62.84 | 3.17E-11 | 3.81E-08 |
| GO:0012507 | ER to Golgi transport vesicle membrane | 7 | 0.06 | 30.67 | 9.50E-08 | 1.14E-04 |
| GO:0005765 | Lysosomal membrane | 10 | 0.09 | 8.31 | 2.83E-06 | 3.40E-03 |
| GO:0000139 | Golgi membrane | 13 | 0.12 | 5.01 | 8.53E-06 | 1.03E-02 |
| GO:0005887 | Integral component of plasma membrane | 19 | 0.17 | 3.06 | 2.70E-05 | 3.25E-02 |
| GO:0009986 | Cell surface | 11 | 0.10 | 4.62 | 1.14E-04 | 0.14 |
| GO:0016020 | Membrane | 22 | 0.20 | 2.28 | 3.33E-04 | 0.4 |
| GO:0030658 | Transport vesicle membrane | 4 | 0.04 | 23.98 | 5.93E-04 | 0.7 |
| GO:0005886 | Plasma membrane | 32 | 0.29 | 1.77 | 6.48E-04 | 0.78 |
| GO:0030669 | Clathrin-coated endocytic vesicle membrane | 4 | 0.04 | 22.22 | 7.42E-04 | 0.89 |
| GO:0042612 | MHC class I protein complex | 3 | 0.03 | 62.13 | 9.95E-04 | 1 |

**Supplemental Table S6. Significant GO-terms of biological process enriched by childhood-onset asthma-relevant genes (N = 83) identified from Sherlock Bayesian analysis in the discovery dataset and MAGMA-based analysis**

| **GO-ID** | **GO terms** | **Gene number** | **Gene proportion** | **Fold Enrichment** | **P-value** | **FDR** |
| --- | --- | --- | --- | --- | --- | --- |
| GO:0006955 | Immune response | 17 | 0.16 | 8.81 | 5.02E-11 | 7.46E-08 |
| GO:0002504 | Antigen processing and presentation of peptide or polysaccharide antigen via MHC class II | 7 | 0.06 | 89.80 | 8.36E-11 | 1.24E-07 |
| GO:0019882 | Antigen processing and presentation | 9 | 0.08 | 35.69 | 1.24E-10 | 1.84E-07 |
| GO:0019886 | Antigen processing and presentation of exogenous peptide antigen via MHC class II | 10 | 0.09 | 23.70 | 3.14E-10 | 4.67E-07 |
| GO:0060333 | Interferon-gamma-mediated signaling pathway | 8 | 0.07 | 24.57 | 3.09E-08 | 4.60E-05 |
| GO:0050852 | T cell receptor signaling pathway | 8 | 0.07 | 11.79 | 4.71E-06 | 7.00E-03 |
| GO:0016045 | Detection of bacterium | 4 | 0.04 | 67.10 | 2.47E-05 | 3.66E-02 |
| GO:0002486 | Antigen processing and presentation of endogenous peptide antigen via MHC class I via ER pathway, TAP-independent | 3 | 0.03 | 218.08 | 6.05E-05 | 8.98E-02 |
| GO:0050776 | Regulation of immune response | 7 | 0.06 | 8.58 | 1.54E-04 | 0.23 |
| GO:0002479 | Antigen processing and presentation of exogenous peptide antigen via MHC class I, TAP-dependent | 5 | 0.05 | 17.31 | 1.89E-04 | 0.28 |
| GO:0002474 | Antigen processing and presentation of peptide antigen via MHC class I | 4 | 0.04 | 29.08 | 3.31E-04 | 0.49 |
| GO:0002480 | Antigen processing and presentation of exogenous peptide antigen via MHC class I, TAP-independent | 3 | 0.03 | 72.69 | 7.13E-04 | 1 |
| GO:0032729 | Positive regulation of interferon-gamma production | 4 | 0.04 | 18.96 | 1.18E-03 | 1 |

**Supplemental Table S7. Disease-related gene sets in GLAD4U database significantly enriched by childhood-onset asthma-relevant genes (N = 83) identified from Sherlock Bayesian analysis in the discovery dataset and MAGMA-based analysis**

| **Gene Set** | **Description** | **Gene size** | **Expect** | **Ratio** | **P value** | **FDR** |
| --- | --- | --- | --- | --- | --- | --- |
| PA443464 | Autoimmune Diseases | 497 | 2.67 | 8.98 | 0 | 0 |
| PA444602 | Immune System Diseases | 739 | 3.97 | 6.79 | 4.44E-16 | 6.00E-13 |
| PA446882 | Genetic Predisposition to Disease | 848 | 4.56 | 6.14 | 1.33E-15 | 1.20E-12 |
| PA443919 | Disease Susceptibility | 880 | 4.73 | 5.49 | 2.55E-13 | 1.72E-10 |
| PA446038 | Virus Diseases | 562 | 3.02 | 6.62 | 9.85E-12 | 5.32E-09 |
| PA443430 | Arthritis | 403 | 2.17 | 7.84 | 3.27E-11 | 1.47E-08 |
| PA444546 | Hypersensitivity | 296 | 1.59 | 9.42 | 4.22E-11 | 1.63E-08 |
| PA445265 | Pathologic Processes | 552 | 2.97 | 6.40 | 6.35E-11 | 2.02E-08 |
| PA445579 | Rubella | 66 | 0.35 | 25.36 | 6.73E-11 | 2.02E-08 |
| PA443780 | Connective Tissue Diseases | 372 | 2.00 | 8.00 | 1.01E-10 | 2.73E-08 |
| PA444329 | Graves Disease | 141 | 0.76 | 14.51 | 2.19E-10 | 5.38E-08 |
| PA444819 | Lupus erythematosus | 232 | 1.25 | 10.42 | 2.79E-10 | 6.12E-08 |
| PA444822 | Lupus Erythematosus, Systemic | 233 | 1.25 | 10.37 | 2.94E-10 | 6.12E-08 |
| PA165108939 | Simian acquired immune deficiency syndrome | 55 | 0.30 | 27.05 | 4.79E-10 | 9.25E-08 |
| PA444890 | Measles | 61 | 0.33 | 24.39 | 1.13E-09 | 2.04E-07 |
| PA443888 | Diabetes Mellitus, Type 1 | 171 | 0.92 | 11.96 | 1.73E-09 | 2.92E-07 |
| PA446861 | Hepatitis, Autoimmune | 42 | 0.23 | 30.99 | 2.29E-09 | 3.43E-07 |
| PA443815 | Crohn Disease | 223 | 1.20 | 10.01 | 2.29E-09 | 3.43E-07 |
| PA446116 | Inflammatory Bowel Diseases | 277 | 1.49 | 8.73 | 2.45E-09 | 3.48E-07 |
| PA444614 | Infection | 620 | 3.33 | 5.40 | 3.36E-09 | 4.46E-07 |
| PA128407009 | Mycobacterium Infections | 140 | 0.75 | 13.28 | 3.63E-09 | 4.46E-07 |
| PA165108143 | Mycobacterial infection | 140 | 0.75 | 13.28 | 3.63E-09 | 4.46E-07 |
| PA445724 | Spondylitis, Ankylosing | 103 | 0.55 | 16.25 | 3.89E-09 | 4.58E-07 |
| PA443374 | Ankylosis | 73 | 0.39 | 20.38 | 4.90E-09 | 5.52E-07 |
| PA443250 | Acquired Immunodeficiency Syndrome | 145 | 0.78 | 12.82 | 5.11E-09 | 5.53E-07 |
| PA444111 | Exophthalmos | 79 | 0.42 | 18.83 | 9.26E-09 | 9.63E-07 |
| PA444987 | Multiple Sclerosis | 207 | 1.11 | 9.88 | 1.28E-08 | 1.29E-06 |
| PA443652 | Celiac Disease | 163 | 0.88 | 11.41 | 1.58E-08 | 1.53E-06 |
| PA443450 | Asthma | 267 | 1.44 | 8.36 | 1.73E-08 | 1.61E-06 |
| PA165108310 | Dermatitis medicamentosa | 33 | 0.18 | 33.81 | 1.96E-08 | 1.77E-06 |
| PA443315 | Alopecia Areata | 35 | 0.19 | 31.88 | 2.85E-08 | 2.48E-06 |
| PA166048719 | Gram-Positive Bacterial Infections | 174 | 0.94 | 10.69 | 2.95E-08 | 2.49E-06 |
| PA445723 | Spondylitis | 130 | 0.70 | 12.87 | 3.04E-08 | 2.49E-06 |
| PA443936 | Drug Hypersensitivity | 94 | 0.51 | 15.82 | 3.70E-08 | 2.90E-06 |
| PA443434 | Arthritis, Rheumatoid | 287 | 1.54 | 7.77 | 3.85E-08 | 2.90E-06 |
| PA444661 | Joint Diseases | 349 | 1.88 | 6.93 | 3.86E-08 | 2.90E-06 |
| PA166048822 | Actinomycetales Infections | 135 | 0.73 | 12.40 | 4.23E-08 | 3.05E-06 |
| PA444745 | Leprosy | 63 | 0.34 | 20.66 | 4.28E-08 | 3.05E-06 |
| PA445555 | Rheumatic Diseases | 297 | 1.60 | 7.51 | 5.61E-08 | 3.88E-06 |
| PA445011 | Myelitis | 20 | 0.11 | 46.48 | 5.74E-08 |  |
| PA445738 | Stevens-Johnson Syndrome | 24 | 0.13 | 38.74 | 1.55E-07 | 9.03E-06 |
| PA444253 | Gastroenteritis | 272 | 1.46 | 7.52 | 2.09E-07 | 1.16E-05 |
| PA444435 | Hepatitis | 233 | 1.25 | 7.98 | 4.54E-07 | 2.36E-05 |
| PA445678 | Skin Diseases, Genetic | 368 | 1.98 | 6.06 | 5.68E-07 | 2.79E-05 |
| PA444320 | Graft vs Host Disease | 95 | 0.51 | 13.70 | 7.46E-07 | 3.48E-05 |
| PA446113 | Cholangitis, Sclerosing | 34 | 0.18 | 27.34 | 9.72E-07 | 4.38E-05 |
| PA444485 | Hodgkin Disease | 153 | 0.82 | 9.72 | 1.60E-06 | 6.54E-05 |
| PA166120929 | severe cutaneous adverse reactions | 17 | 0.09 | 43.75 | 1.75E-06 | 7.04E-05 |
| PA447230 | HIV | 854 | 4.59 | 3.70 | 2.16E-06 | 8.35E-05 |
| PA165109019 | Sympathetic uveitis | 6 | 0.03 | 92.97 | 2.96E-06 | 1.07E-04 |
| PA166048890 | Respiratory Hypersensitivity | 227 | 1.22 | 7.37 | 3.42E-06 | 1.22E-04 |
| PA445640 | Sexually Transmitted Diseases | 464 | 2.50 | 4.81 | 6.32E-06 | 2.02E-04 |
| PA445098 | Neuritis | 23 | 0.12 | 32.34 | 6.34E-06 | 2.02E-04 |
| PA444740 | Leishmaniasis, Mucocutaneous | 8 | 0.04 | 69.73 | 8.22E-06 | 2.50E-04 |
| PA443854 | Demyelinating Diseases | 198 | 1.06 | 7.51 | 1.08E-05 | 3.11E-04 |
| PA162316739 | Maculopapular Exanthema | 10 | 0.05 | 55.78 | 1.75E-05 | 4.68E-04 |
| PA443474 | Bacterial Infections | 258 | 1.39 | 5.77 | 7.17E-05 | 1.63E-03 |
| PA445811 | Takayasu Arteritis | 24 | 0.13 | 23.24 | 2.79E-04 | 5.06E-03 |
| PA165108299 | Unspecified optic neuritis | 24 | 0.13 | 23.24 | 2.79E-04 | 5.06E-03 |
| PA133888799 | Organ Transplantation | 144 | 0.77 | 6.46 | 1.06E-03 |  |
| PA444849 | Lymphoproliferative Disorders | 387 | 2.08 | 3.84 | 1.10E-03 | 1.74E-02 |
| PA445418 | Proctitis | 10 | 0.05 | 37.19 | 1.25E-03 | 1.93E-02 |
| PA166048810 | Respiratory Syncytial Virus Infections | 227 | 1.22 | 4.91 | 1.38E-03 | 2.12E-02 |
| PA446227 | Retinal Dysplasia | 44 | 0.24 | 12.68 | 1.69E-03 | 2.54E-02 |
| PA444206 | Food Hypersensitivity | 46 | 0.25 | 12.13 | 1.92E-03 | 2.84E-02 |
| PA166115462 | dry mouth | 53 | 0.29 | 10.53 | 2.89E-03 | 4.03E-02 |
| PA165108509 | Disease due to Hantanvirus | 17 | 0.09 | 21.88 | 3.69E-03 | 4.88E-02 |
| PA166048858 | Red-Cell Aplasia, Pure | 17 | 0.09 | 21.88 | 3.69E-03 | 4.88E-02 |
| PA444704 | Premature Birth | 196 | 1.05 | 4.74 | 4.07E-03 | 5.32E-02 |
| PA445109 | Neuromyelitis Optica | 19 | 0.10 | 19.57 | 4.60E-03 | 5.90E-02 |
| PA443879 | Dermatitis, Atopic | 126 | 0.68 | 5.90 | 4.72E-03 | 6.02E-02 |
| PA446408 | Diabetic Ketoacidosis | 21 | 0.11 | 17.71 | 5.61E-03 | 6.90E-02 |

**Supplemental Table S8. Disease-related gene sets in DisGeNET database significantly enriched by childhood-onset asthma-relevant genes (N = 83) identified from Sherlock Bayesian analysis in the discovery dataset and MAGMA-based analysis**

| **Gene Set** | **Description** | **Gene size** | **Expect** | **Ratio** | **P value** | **FDR** |
| --- | --- | --- | --- | --- | --- | --- |
| C0013182 | Drug Allergy | 35 | 0.22 | 22.27 | 2.48E-06 | 9.02E-03 |
| C4020969 | Inflammatory abnormality of the eye | 10 | 0.064 | 46.76 | 2.89E-05 | 5.25E-02 |
| C0024141 | Lupus Erythematosus, Systemic | 65 | 0.42 | 11.99 | 5.46E-05 | 6.62E-02 |

**Supplemental Table S9. Drug-related gene sets in GeneBank database significantly enriched by childhood-onset asthma-relevant genes (N = 83) identified from Sherlock Bayesian analysis in the discovery dataset and MAGMA-based analysis**

| **Gene Set** | **Description** | **Gene size** | **Expect** | **Ratio** | **P value** | **FDR** |
| --- | --- | --- | --- | --- | --- | --- |
| DB01053 | Benzylpenicillin | 11 | 0.06 | 53.26 | 1.75E-05 | 2.05E-02 |
| DB01165 | Ofloxacin | 7 | 0.04 | 55.79 | 5.07E-04 | 8.49E-02 |
| DB01140 | Cefadroxil | 5 | 0.03 | 78.11 | 2.43E-04 | 8.49E-02 |
| DB00415 | Ampicillin | 6 | 0.03 | 65.09 | 3.63E-04 | 8.49E-02 |
| DB00535 | Cefdinir | 6 | 0.03 | 65.09 | 3.63E-04 | 8.49E-02 |
| DB00332 | Ipratropium | 7 | 0.04 | 55.79 | 5.07E-04 | 8.49E-02 |
| DB01333 | Cefradine | 5 | 0.03 | 78.11 | 2.43E-04 | 8.49E-02 |
| DB08837 | Tetraethylammonium | 8 | 0.04 | 48.82 | 6.74E-04 | 8.78E-02 |
| DB00456 | Cefalotin | 8 | 0.04 | 48.82 | 6.74E-04 | 8.78E-02 |
| DB01409 | Tiotropium | 9 | 0.05 | 43.39 | 8.63E-04 | 9.21E-02 |
| DB00567 | Cephalexin | 9 | 0.05 | 43.39 | 8.63E-04 | 9.21E-02 |
| DB00536 | Guanidine | 10 | 0.05 | 39.05 | 1.08E-03 | 9.71E-02 |
| DB01035 | Procainamide | 10 | 0.05 | 39.05 | 1.08E-03 | 9.71E-02 |

**Supplemental Table S10. Drug-related gene sets in GLAD4U database significantly enriched by childhood-onset asthma-relevant genes (N = 83) identified from Sherlock Bayesian analysis in the discovery dataset and MAGMA-based analysis**

| **Gene Set** | **Description** | **Gene size** | **Expect** | **Ratio** | **P value** | **FDR** |
| --- | --- | --- | --- | --- | --- | --- |
| PA164713242 | Rubella vaccines | 59 | 0.36 | 27.56 | 2.10E-12 | 3.85E-09 |
| PA164712817 | Immunoglobulins | 476 | 2.93 | 6.49 | 4.96E-11 | 3.02E-08 |
| PA164754884 | immune globulin | 476 | 2.93 | 6.49 | 4.96E-11 | 3.02E-08 |
| PA164712887 | Measles vaccines | 43 | 0.26 | 30.25 | 1.73E-10 | 7.89E-08 |
| PA164712916 | Mumps vaccines | 45 | 0.28 | 28.91 | 2.54E-10 | 9.30E-08 |
| PA164713390 | Viral Vaccines | 129 | 0.79 | 12.60 | 5.85E-09 | 1.78E-06 |
| PA164742987 | clavulanate | 7 | 0.04 | 92.91 | 4.57E-08 | 1.19E-05 |
| PA164713092 | Other immunoglobulins | 226 | 1.39 | 7.91 | 1.23E-07 | 2.81E-05 |
| PA164713378 | Vaccines | 184 | 1.13 | 8.84 | 1.73E-07 | 3.52E-05 |
| PA164713015 | Other anticestodals | 201 | 1.24 | 8.09 | 3.94E-07 | 7.20E-05 |
| PA164713274 | Specific immunoglobulins | 286 | 1.76 | 6.25 | 1.29E-06 | 2.14E-04 |
| PA164769031 | lumiracoxib | 5 | 0.03 | 97.55 | 2.22E-06 | 3.38E-04 |
| PA164712789 | Hepatitis vaccines | 36 | 0.22 | 22.58 | 2.51E-06 | 3.53E-04 |
| PA450616 | nevirapine | 17 | 0.10 | 38.26 | 2.97E-06 | 3.87E-04 |
| PA166123556 | beryllium | 19 | 0.12 | 34.23 | 4.78E-06 | 5.83E-04 |
| PA451999 | interferons | 323 | 1.99 | 5.03 | 2.71E-05 | 3.09E-03 |
| PA164712997 | Other Vaccines | 99 | 0.61 | 9.85 | 3.17E-05 | 3.41E-03 |
| PA164713019 | Other antiepileptics | 38 | 0.23 | 17.12 | 8.34E-05 | 8.46E-03 |
| PA450164 | lamotrigine | 41 | 0.25 | 15.86 | 1.13E-04 | 1.08E-02 |
| PA450732 | oxcarbazepine | 16 | 0.10 | 30.49 | 1.18E-04 | 1.08E-02 |
| PA164712554 | Beta-lactamase inhibitors | 18 | 0.11 | 27.10 | 1.71E-04 | 1.42E-02 |
| PA166115521 | trichloroethylene | 18 | 0.11 | 27.10 | 1.71E-04 | 1.42E-02 |
| PA164743471 | adenosine triphosphate | 331 | 2.04 | 4.42 | 1.89E-04 | 1.50E-02 |
| PA164748040 | alefacept | 5 | 0.03 | 65.04 | 3.69E-04 | 2.70E-02 |
| PA450082 | ipratropium | 5 | 0.03 | 65.04 | 3.69E-04 | 2.70E-02 |
| PA164712819 | Immunostimulants | 220 | 1.35 | 5.17 | 4.00E-04 | 2.81E-02 |
| PA452154 | beta-lactam antibacterials, penicillins | 25 | 0.15 | 19.51 | 4.67E-04 | 3.16E-02 |
| PA452174 | antivirals | 394 | 2.42 | 3.71 | 6.75E-04 | 4.40E-02 |

**Supplemental Table S11. Multiple top-ranked eSNPs identified in 31 candidate genes implicated in childhood-onset asthma risk**

| **Gene name** | **SNP ID** | **Chromosome** | **Position** | **Proximity** | **eQTL P values** | **GWAS P values** | **LBF** | **Gene resources** |
| --- | --- | --- | --- | --- | --- | --- | --- | --- |
| *HLA-DQA1* | rs17426593 | 6 | 32716055 | cis | 2.29E-102 | 9.02E-19 | 7.18 | Dataset #2 |
| *HLA-DQA1* | rs1150753 | 6 | 32167845 | cis | 3.28E-26 | 7.29E-07 | 6.47 | Dataset #2 |
| *HLA-DQA1* | rs9393713 | 6 | 26481657 | trans | 8.95E-06 | 4.27E-02 | -0.10 | Dataset #2 |
| *HLA-DQA1* | rs10484399 | 6 | 27642507 | trans | 1.34E-08 | 2.38E-03 | 0.40 | Dataset #2 |
| *HLA-DQA1* | rs660895 | 6 | 32685358 | cis | 4.90E-11 | 9.79E-17 | 4.83 | Dataset #3 |
| *PSMB9* | rs4148882 | 6 | 32924936 | cis | 2.32E-19 | 5.80E-09 | 6.90 | Dataset #2 |
| *PSMB9* | rs2071534 | 6 | 32932296 | cis | 1.30E-08 | 8.49E-06 | 1.73 | Dataset #3 |
| *HLA-DRB5* | rs385492 | 6 | 29757526 | trans | 5.24E-06 | 4.34E-02 | -0.33 | Dataset #2 |
| *HLA-DRB5* | rs1150753 | 6 | 32167845 | cis | 8.99E-09 | 7.29E-07 | 6.12 | Dataset #2 |
| *HLA-DRB5* | rs9272346 | 6 | 32712350 | cis | 2.15E-100 | 4.58E-55 | 6.84 | Dataset #2 |
| *HLA-DRB5* | rs2071295 | 6 | 32146678 | cis | 2.00E-04 | 1.18E-07 | 3.63 | Dataset #3 |
| *HLA-DRB5* | rs7383287 | 6 | 32891064 | cis | 5.00E-04 | 5.11E-03 | -0.03 | Dataset #3 |
| *HLA-DPB1* | rs13195040 | 6 | 27521903 | trans | 9.29E-06 | 9.38E-03 | 0.11 | Dataset #2 |
| *HLA-DPB1* | rs1150753 | 6 | 32167845 | cis | 4.49E-18 | 7.29E-07 | 5.92 | Dataset #2 |
| *HLA-DPB1* | rs429916 | 6 | 33086565 | cis | 1.91E-75 | 8.61E-13 | 5.69 | Dataset #2 |
| *HLA-DPB1* | rs2855430 | 6 | 33249258 | cis | 1.30E-07 | 3.00E-11 | 6.90 | Dataset #3 |
| *HLA-DRB1* | rs10484399 | 6 | 27642507 | trans | 3.45E-11 | 2.38E-03 | 0.33 | Dataset #2 |
| *HLA-DRB1* | rs385492 | 6 | 29757526 | trans | 1.13E-06 | 4.34E-02 | -0.72 | Dataset #2 |
| *HLA-DRB1* | rs1150753 | 6 | 32167845 | cis | 9.88E-26 | 7.29E-07 | 6.10 | Dataset #2 |
| *HLA-DRB1* | rs9272346 | 6 | 32712350 | cis | 2.46E-128 | 4.58E-55 | 6.71 | Dataset #2 |
| *HLA-DRB1* | rs11235093 | 11 | 71200966 | trans | 6.24E-06 | 1.25E-02 | -0.06 | Dataset #2 |
| *HLA-DRB1* | rs3856348 | 2 | 211054241 | trans | 1.00E-05 | 1.75E-02 | -0.07 | Dataset #3 |
| *HLA-DRB1* | rs660895 | 6 | 32685358 | cis | 1.90E-10 | 9.79E-17 | 4.94 | Dataset #3 |
| *TAP2* | rs9267798 | 6 | 32152812 | cis | 6.68E-06 | 8.72E-04 | 3.44 | Dataset #2 |
| *TAP2* | rs4148882 | 6 | 32924936 | cis | 3.71E-11 | 5.80E-09 | 6.21 | Dataset #2 |
| *TAP2* | rs10447456 | 6 | 96689741 | trans | 1.23E-06 | 5.79E-03 | -0.22 | Dataset #2 |
| *TAP2* | rs241456 | 6 | 32903943 | cis | 1.40E-16 | 2.16E-27 | 7.32 | Dataset #3 |
| *TLR6* | rs5743592 | 4 | 38479458 | cis | 1.37E-08 | 2.92E-35 | 7.53 | Dataset #2 |
| *TLR6* | rs5743595 | 4 | 38479039 | cis | 4.50E-07 | 2.54E-35 | 6.45 | Dataset #3 |
| *PMM1* | rs203319 | 22 | 40244539 | cis | 1.23E-11 | 9.19E-10 | 7.42 | Dataset #2 |
| *PMM1* | rs132774 | 22 | 40361899 | cis | 2.50E-06 | 8.57E-08 | 6.36 | Dataset #3 |
| *MPHOSPH9* | rs7299943 | 12 | 122159438 | cis | 2.08E-13 | 8.84E-09 | 7.44 | Dataset #2 |
| *MPHOSPH9* | rs1716160 | 12 | 122266178 | cis | 7.00E-05 | 1.38E-08 | 5.13 | Dataset #2 |
| *MPHOSPH9* | rs1716382 | 12 | 123190418 | cis | 3.00E-04 | 9.43E-03 | -0.06 | Dataset #3 |
| *PSMD3* | rs12311409 | 12 | 79098520 | trans | 6.80E-06 | 8.72E-01 | -0.08 | Dataset #2 |
| *PSMD3* | rs8077456 | 17 | 35382291 | cis | 6.34E-06 | 1.98E-28 | 7.15 | Dataset #3 |
| *HLA-DOB* | rs1343898 | 2 | 143839581 | trans | 8.96E-06 | 1.75E-02 | -0.05 | Dataset #2 |
| *HLA-DOB* | rs2071474 | 6 | 32890560 | cis | 7.75E-49 | 3.83E-28 | 7.06 | Dataset #2 |
| *HLA-DOB* | rs10831284 | 11 | 94307612 | trans | 8.44E-06 | 3.28E-02 | -0.06 | Dataset #2 |
| *HLA-DOB* | rs2621332 | 6 | 32888295 | cis | 2.80E-06 | 7.82E-17 | 6.12 | Dataset #3 |
| *ME2* | rs1810129 | 18 | 46656070 | cis | 1.49E-10 | 9.74E-08 | 6.70 | Dataset #2 |
| *ME2* | rs584357 | 18 | 46699247 | cis | 4.00E-04 | 6.22E-07 | 3.31 | Dataset #3 |
| *ACTR1A* | rs2281879 | 10 | 104258867 | cis | 1.22E-07 | 3.08E-07 | 7.01 | Dataset #2 |
| *ACTR1A* | rs17709558 | 17 | 48694377 | trans | 8.63E-06 | 3.71E-02 | -0.06 | Dataset #2 |
| *ACTR1A* | rs1293426 | 20 | 51171756 | trans | 1.72E-06 | 2.67E-02 | -0.18 | Dataset #2 |
| *ACTR1A* | rs10883723 | 10 | 104215822 | cis | 2.00E-05 | 1.10E-07 | 5.62 | Dataset #3 |
| *DEXI* | rs10847067 | 12 | 125134194 | trans | 8.39E-06 | 3.99E-02 | -0.04 | Dataset #2 |
| *DEXI* | rs12935657 | 16 | 11126542 | cis | 1.71E-18 | 6.13E-26 | 7.00 | Dataset #2 |
| *DEXI* | rs741175 | 16 | 11067186 | cis | 6.00E-06 | 7.85E-12 | 6.49 | Dataset #3 |
| *NSMCE1* | rs3024530 | 16 | 27258188 | cis | 1.28E-14 | 1.10E-13 | 7.25 | Dataset #2 |
| *NSMCE1* | rs6498001 | 16 | 27124740 | cis | 4.00E-04 | 3.32E-10 | 3.24 | Dataset #3 |
| *JAZF1* | rs2189966 | 7 | 28138591 | cis | 1.08E-10 | 8.60E-15 | 6.39 | Dataset #2 |
| *JAZF1* | rs3108397 | 4 | 123237584 | trans | 1.40E-06 | 4.12E-02 | -0.29 | Dataset #3 |
| *JAZF1* | rs1635852 | 7 | 28155936 | cis | 2.00E-04 | 2.41E-07 | 4.24 | Dataset #3 |
| *JAZF1* | rs10469016 | 18 | 48580883 | trans | 6.30E-06 | 4.93E-02 | -0.10 | Dataset #3 |
| *TDRKH* | rs868867 | 1 | 150028489 | cis | 2.01E-14 | 6.55E-11 | 6.38 | Dataset #2 |
| *TDRKH* | rs1054475 | 1 | 150012859 | cis | 7.40E-06 | 2.41E-09 | 5.31 | Dataset #3 |
| *TDRKH* | rs2181590 | 20 | 60056238 | trans | 1.00E-05 | 1.84E-02 | -0.06 | Dataset #3 |
| *SMARCE1* | rs7633974 | 3 | 38640927 | trans | 1.57E-06 | 1.30E-02 | -0.19 | Dataset #2 |
| *SMARCE1* | rs2569885 | 6 | 1609674 | trans | 1.84E-06 | 1.79E-02 | 0.03 | Dataset #2 |
| *SMARCE1* | rs703992 | 10 | 80597657 | trans | 5.82E-06 | 3.41E-03 | -0.07 | Dataset #2 |
| *SMARCE1* | rs1358174 | 17 | 36011285 | cis | 1.87E-36 | 1.27E-13 | 6.65 | Dataset #2 |
| *SMARCE1* | rs757411 | 17 | 36028676 | cis | 4.70E-14 | 3.58E-10 | 6.22 | Dataset #3 |
| *SMARCE1* | rs2145078 | 20 | 29482203 | trans | 8.20E-06 | 3.69E-04 | -0.07 | Dataset #3 |
| *AHI1* | rs2757649 | 6 | 135768306 | cis | 6.06E-26 | 1.03E-07 | 4.64 | Dataset #2 |
| *AHI1* | rs2014355 | 12 | 119659907 | trans | 1.70E-06 | 4.68E-07 | 4.83 | Dataset #2 |
| *AHI1* | rs9393638 | 6 | 10866876 | trans | 4.20E-06 | 3.99E-02 | -0.14 | Dataset #3 |
| *AHI1* | rs11154801 | 6 | 135781048 | cis | 2.20E-30 | 5.31E-07 | 4.02 | Dataset #3 |
| *AHI1* | rs7812506 | 8 | 80699907 | trans | 6.30E-06 | 1.09E-02 | -0.09 | Dataset #3 |
| *AHI1* | rs7006113 | 8 | 99148091 | trans | 1.00E-05 | 2.08E-02 | -0.06 | Dataset #3 |
| *SLC15A2* | rs9826473 | 3 | 123038909 | cis | 2.89E-10 | 2.71E-07 | 5.92 | Dataset #2 |
| *SLC15A2* | rs1806656 | 3 | 123198861 | cis | 2.60E-13 | 6.30E-10 | 6.52 | Dataset #3 |
| *SLC15A2* | rs6991424 | 8 | 98872905 | trans | 7.90E-06 | 3.43E-02 | -0.10 | Dataset #3 |
| *RAD50* | rs2069812 | 5 | 131907815 | cis | 5.18E-11 | 8.66E-10 | 7.60 | Dataset #2 |
| *RAD50* | rs1458979 | 3 | 55125717 | trans | 6.50E-06 | 1.46E-02 | -0.09 | Dataset #3 |
| *RAD50* | rs2069812 | 5 | 131907815 | cis | 1.40E-08 | 8.66E-10 | 6.40 | Dataset #3 |
| *RAD50* | rs7715671 | 5 | 132554450 | cis | 6.00E-04 | 4.57E-02 | -0.03 | Dataset #3 |
| *RAD50* | rs2299460 | 7 | 125982676 | trans | 9.30E-06 | 2.38E-02 | -0.07 | Dataset #3 |
| *HLA-B* | rs3130564 | 6 | 31209653 | cis | 9.35E-09 | 2.12E-15 | 6.50 | Dataset #2 |
| *HLA-B* | rs3130564 | 6 | 31209653 | cis | 7.00E-04 | 2.12E-15 | 2.08 | Dataset #3 |
| *LST1* | rs2071593 | 6 | 31620778 | cis | 5.44E-06 | 3.26E-13 | 5.42 | Dataset #2 |
| *LST1* | rs2855812 | 6 | 31580699 | cis | 2.00E-05 | 6.38E-07 | 4.33 | Dataset #3 |
| *LST1* | rs1861856 | 15 | 83999565 | trans | 8.80E-07 | 1.20E-05 | 3.17 | Dataset #3 |
| *POLI* | rs2276182 | 18 | 50052045 | cis | 4.93E-09 | 5.01E-10 | 6.06 | Dataset #2 |
| *POLI* | rs3730783 | 18 | 50069281 | cis | 9.00E-07 | 4.27E-10 | 6.78 | Dataset #3 |
| *HSPA1A* | rs2276133 | 11 | 65318833 | trans | 2.17E-06 | 1.44E-07 | 5.11 | Dataset #2 |
| *HSPA1A* | rs4143780 | 2 | 80028434 | trans | 6.40E-06 | 3.32E-01 | -0.09 | Dataset #3 |
| *SLC22A5* | rs17622656 | 5 | 131848896 | cis | 1.73E-39 | 2.62E-17 | 6.04 | Dataset #2 |
| *SLC22A5* | rs10490083 | 2 | 202586189 | trans | 1.10E-07 | 4.83E-03 | -0.82 | Dataset #3 |
| *SLC22A5* | rs11950562 | 5 | 131680428 | cis | 1.80E-08 | 5.89E-11 | 5.87 | Dataset #3 |
| *CTSW* | rs659824 | 11 | 65393085 | cis | 8.93E-06 | 1.40E-08 | 5.09 | Dataset #2 |
| *CTSW* | rs1440201 | 14 | 32849704 | trans | 7.16E-06 | 3.80E-02 | -0.05 | Dataset #2 |
| *CTSW* | rs494003 | 11 | 65298874 | cis | 2.00E-04 | 2.03E-08 | 3.14 | Dataset #3 |
| *NDFIP1* | rs1793076 | 11 | 106026860 | trans | 3.50E-06 | 4.48E-02 | -0.12 | Dataset #2 |
| *NDFIP1* | rs6498142 | 16 | 10988750 | trans | 4.20E-06 | 6.18E-14 | 4.51 | Dataset #2 |
| *NDFIP1* | rs449454 | 5 | 141513246 | cis | 1.20E-06 | 2.95E-11 | 7.13 | Dataset #3 |
| *HLA-C* | rs12028496 | 1 | 33470027 | trans | 9.68E-07 | 3.54E-02 | -0.30 | Dataset #2 |
| *HLA-C* | rs1063646 | 6 | 31215627 | trans | 3.14E-32 | 1.16E-12 | 4.37 | Dataset #2 |
| *HLA-C* | rs3130564 | 6 | 31209653 | cis | 2.80E-07 | 2.12E-15 | 5.33 | Dataset #3 |
| *HLA-C* | rs652888 | 6 | 31959213 | cis | 4.00E-04 | 1.89E-02 | -0.05 | Dataset #3 |
| *HLA-C* | rs5935799 | X | 14641184 | trans | 4.00E-06 | 7.83E-03 | -0.13 | Dataset #3 |
| *CD52* | rs6703878 | 1 | 26571525 | cis | 6.70E-41 | 8.25E-06 | 1.92 | Dataset #2 |
| *CD52* | rs6728124 | 2 | 67169024 | trans | 3.39E-06 | 3.28E-02 | -0.11 | Dataset #2 |
| *CD52* | rs1071849 | 1 | 26519313 | cis | 1.70E-07 | 7.14E-05 | 1.62 | Dataset #3 |
| *CD52* | rs3780032 | 8 | 140764557 | trans | 1.10E-06 | 9.22E-04 | -0.33 | Dataset #3 |
| *ARL3* | rs10786679 | 10 | 104305657 | cis | 6.46E-06 | 2.58E-05 | 0.93 | Dataset #2 |
| *ARL3* | rs2160203 | 2 | 102327256 | trans | 9.10E-06 | 1.20E-47 | 2.82 | Dataset #3 |

**Supplemental Table S12. Sherlock-identified genes in the discovery stage reported in previous studies**

| **Gene name** | **LBF** | **P value** | **FDR value** | **GWAS documented genes** | **PrediXcan validated genes** | **Genes associated with T1D** | **Genes associated with RA** |
| --- | --- | --- | --- | --- | --- | --- | --- |
| *NOTCH4* | 12.37 | 7.87E-07 | 2.05E-04 | Yes | Yes | Yes | No |
| *HLA-DRB1* | 10.91 | 7.87E-07 | 2.05E-04 | Yes | Yes | Yes | Yes |
| *TAP2* | 8.70 | 7.87E-07 | 2.05E-04 | No | Yes | Yes | Yes |
| *SUOX* | 7.11 | 7.87E-07 | 2.05E-04 | Yes | Yes | Yes | No |
| *IKZF3* | 7.02 | 7.87E-07 | 2.05E-04 | Yes | Yes | Yes | Yes |
| *APOM* | 7.49 | 7.87E-07 | 2.05E-04 | No | Yes | No | Yes |
| *HLA-DQA1* | 12.91 | 7.87E-07 | 2.05E-04 | Yes | Yes | No | Yes |
| *PSMB9* | 12.17 | 7.87E-07 | 2.05E-04 | No | Yes | No | No |
| *HLA-DRB5* | 11.52 | 7.87E-07 | 2.05E-04 | Yes | Yes | No | No |
| *TLR6* | 7.38 | 7.87E-07 | 2.05E-04 | Yes | Yes | No | No |
| *IL18R1* | 7.22 | 7.87E-07 | 2.05E-04 | Yes | Yes | No | No |
| *ADORA1* | 7.79 | 7.87E-07 | 2.05E-04 | Yes | No | No | No |
| *MPHOSPH9* | 7.20 | 7.87E-07 | 2.05E-04 | Yes | No | No | No |
| *DEXI* | 6.42 | 1.57E-06 | 2.05E-04 | No | No | Yes | No |
| *IER3* | 6.23 | 1.57E-06 | 2.05E-04 | No | No | Yes | No |
| *TNFSF4* | 6.60 | 1.57E-06 | 2.05E-04 | Yes | No | No | Yes |
| *JAZF1* | 6.18 | 1.57E-06 | 2.05E-04 | Yes | No | No | Yes |
| *IRF1* | 5.72 | 1.57E-06 | 2.05E-04 | No | Yes | No | Yes |
| *STARD3* | 6.81 | 1.57E-06 | 2.05E-04 | No | Yes | No | No |
| *HLA-DOB* | 6.67 | 1.57E-06 | 2.05E-04 | No | Yes | No | No |
| *D2HGDH* | 6.34 | 1.57E-06 | 2.05E-04 | Yes | Yes | No | No |
| *NSMCE1* | 6.22 | 1.57E-06 | 2.05E-04 | No | Yes | No | No |
| *TDRKH* | 6.18 | 1.57E-06 | 2.05E-04 | Yes | Yes | No | No |
| *IL4R* | 6.15 | 1.57E-06 | 2.05E-04 | Yes | Yes | No | No |
| *HLA-DRB6* | 6.14 | 1.57E-06 | 2.05E-04 | Yes | Yes | No | No |
| *SMARCE1* | 6.06 | 1.57E-06 | 2.05E-04 | Yes | Yes | No | No |
| *RAD50* | 5.60 | 1.57E-06 | 2.05E-04 | Yes | Yes | No | No |
| *PHF5A* | 6.56 | 1.57E-06 | 2.05E-04 | Yes | No | No | No |
| *RERE* | 6.40 | 1.57E-06 | 2.05E-04 | Yes | No | No | No |
| *BRD2* | 6.25 | 1.57E-06 | 2.05E-04 | Yes | No | No | No |
| *STAT6* | 6.08 | 1.57E-06 | 2.05E-04 | Yes | No | No | No |
| *GLB1* | 6.01 | 1.57E-06 | 2.05E-04 | Yes | No | No | No |
| *FCER1G* | 5.79 | 1.57E-06 | 2.05E-04 | Yes | No | No | No |
| *HLA-B* | 5.50 | 1.57E-06 | 2.05E-04 | Yes | No | No | Yes |
| *C5* | 5.76 | 1.57E-06 | 2.05E-04 | No | No | No | Yes |
| *MICA* | 5.03 | 6.30E-06 | 7.39E-04 | No | Yes | Yes | Yes |
| *POLI* | 5.12 | 6.30E-06 | 7.39E-04 | Yes | No | No | No |
| *KLHL5* | 4.96 | 6.30E-06 | 7.39E-04 | Yes | No | No | No |
| *SLC22A5* | 4.54 | 1.42E-05 | 1.33E-03 | Yes | Yes | No | No |
| *TRAF1* | 4.26 | 2.05E-05 | 1.75E-03 | No | No | No | Yes |
| *NDFIP1* | 3.93 | 4.25E-05 | 2.99E-03 | Yes | No | No | No |
| *HLA-C* | 3.71 | 5.83E-05 | 3.73E-03 | No | Yes | No | No |
| *GFRA2* | 3.23 | 1.01E-04 | 5.32E-03 | Yes | No | No | No |
| *NEK6* | 3.12 | 1.26E-04 | 6.28E-03 | No | Yes | No | No |
| *HLA-A* | 3.07 | 1.29E-04 | 6.34E-03 | No | No | Yes | No |
| *MICB* | 3.02 | 1.34E-04 | 6.47E-03 | Yes | Yes | No | No |
| *GLI3* | 3.00 | 1.37E-04 | 6.52E-03 | Yes | No | No | No |
| *PEX14* | 2.81 | 1.68E-04 | 7.46E-03 | Yes | No | No | No |
| *PGM1* | 2.65 | 2.00E-04 | 8.41E-03 | No | No | Yes | No |
| *TNPO3* | 2.60 | 2.09E-04 | 8.67E-03 | No | No | No | Yes |
| *KIF11* | 2.36 | 2.79E-04 | 1.07E-02 | Yes | No | No | No |
| *SPPL3* | 2.25 | 3.07E-04 | 1.13E-02 | No | Yes | No | No |
| *PHTF1* | 1.89 | 4.76E-04 | 1.56E-02 | No | No | Yes | No |
| *RNASEH2B* | 1.87 | 4.88E-04 | 1.59E-02 | No | No | No | Yes |
| *PLD4* | 1.51 | 7.05E-04 | 2.05E-02 | No | No | No | Yes |
| *FAM120B* | 1.48 | 7.24E-04 | 2.08E-02 | No | No | Yes | No |
| *CD52* | 1.42 | 7.75E-04 | 2.19E-02 | No | Yes | No | No |
| *STAT4* | 1.08 | 1.11E-03 | 2.97E-02 | No | No | No | Yes |
| *IL18RAP* | 1.01 | 1.19E-03 | 3.13E-02 | No | Yes | No | No |
| *IL2RB* | 0.64 | 1.73E-03 | 4.19E-02 | Yes | No | Yes | Yes |
| *HLA-DOA* | 0.61 | 1.79E-03 | 4.30E-02 | Yes | No | No | No |
| *ARRDC1* | 0.44 | 2.15E-03 | 4.88E-02 | Yes | No | No | No |
| *CSAD* | 0.43 | 2.20E-03 | 4.98E-02 | No | No | Yes | No |

Note: T1D represents type 1 diabetes, RA represents rheumatoid arthritis.

**Supplemental Table S13. Genetic correlations between childhood onset asthma and other six autoimmune diseases**

| **Trait1** | **Trait2** | **Genetic correlation** | **SE** | **Z-score** | **P-value** |
| --- | --- | --- | --- | --- | --- |
| Childhood onset asthma | Coeliac disease | 0.09 | 0.09 | 0.98 | 0.33 |
| Childhood onset asthma | Crohn disease | 0.03 | 0.05 | 0.68 | 0.50 |
| Childhood onset asthma | Multiple sclerosis | 0.07 | 0.14 | 0.50 | 0.62 |
| Childhood onset asthma | Primary biliary cirrhosis | -0.20 | 0.14 | -1.43 | 0.15 |
| Childhood onset asthma | Rheumatoid arthritis | -0.03 | 0.09 | -0.29 | 0.77 |
| Childhood onset asthma | Type I diabetes | -0.04 | 0.10 | -0.38 | 0.70 |

**Note:** We selected six autoimmune diseases including Coeliac disease, Crohn disease, multiple sclerosis, primary biliary cirrhosis, and type I diabetes with GWAS summary statistics from the UK-Biobank database to calculate the genetic correlations with childhood onset asthma by using LD score regression (LDSC, version 1.0.1)

**Supplemental Table S14. Colocalization analysis for childhood onset asthma with other six autoimmune diseases**

| **Trait 1** | **Trait 2** | **SNP** | **Posterior probability (PP4)** |
| --- | --- | --- | --- |
| Childhood onset asthma | Coeliac disease | rs4795399 | 0.58 |
| Childhood onset asthma | Coeliac disease | rs11078926 | 0.24 |
| Childhood onset asthma | Crohn disease | rs4795399 | 0.79 |
| Childhood onset asthma | Crohn disease | rs2305480 | 0.21 |
| Childhood onset asthma | Multiple sclerosis | rs4795399 | 0.58 |
| Childhood onset asthma | Multiple sclerosis | rs11078926 | 0.24 |
| Childhood onset asthma | Primary biliary cirrhosis | rs4795399 | 0.64 |
| Childhood onset asthma | Primary biliary cirrhosis | rs11078926 | 0.18 |
| Childhood onset asthma | Primary biliary cirrhosis | rs2305480 | 0.18 |
| Childhood onset asthma | Rheumatoid arthritis | rs4795399 | 0.57 |
| Childhood onset asthma | Rheumatoid arthritis | rs11078926 | 0.24 |
| Childhood onset asthma | Type I diabetes | rs4795399 | 0.44 |
| Childhood onset asthma | Type I diabetes | rs11078926 | 0.32 |
| Childhood onset asthma | Type I diabetes | rs2305480 | 0.24 |

**Note:** We selected six autoimmune diseases including Coeliac disease, Crohn disease, multiple sclerosis, primary biliary cirrhosis, and type I diabetes with GWAS summary statistics from the UK-Biobank database to conduct colocalization analysis with childhood onset asthma by using *coloc* package in R platform.
