# Supplementary material for: Integrative genomics analysis of various omics data and networks identify risk genes and variants vulnerable to childhood-onset asthma
Source: BMC Med Genomics. 2020 Aug 31;13:123. doi: 10.1186/s12920-020-00768-z (PMC7457797; doi:10.1186/s12920-020-00768-z)
Supplement: Supplementary file 2 — Additional file 2: Figure S1. Disease-based enrichment analysis of 83 childhood-onset asthma-related genes based on the DisGeNET database. Figure S2. Drug-based enrichment analysis of 83 childhood-onset asthma-related genes based on the Drugbank database. Figure S3. Drug-based enrichment analysis of 83 childhood-onset asthma-related genes based on the GLAD4U database. Figure S4. Consistent evidence of childhood-onset asthma-relevant genes based on independent datasets and techniques. a) Venn diagram of three identified childhood-onset asthma-relevant gene sets. b) Computer-based permutation analysis of 105 times for the comparison of genes from dataset #3 with that from MAGMA analysis (raw P value of each gene was applied). c) Computer-based permutation analysis of 105 times for the comparison of genes from dataset #3 with that from dataset #4 (raw P value of each gene was applied). Figure S5. Density plot show the differences of co-expression patterns between childhood-onset asthma (CoA) and matched controls. Figure S6. Boxplots show the differential expression profiles of 4 genes between mild-to-moderate asthma and severe asthma group. Figure S7. Boxplots show the differential expression profiles of 6 genes among control, convalescence, and severe asthma group. [file 12920_2020_768_MOESM2_ESM.pptx]

## Slide 1
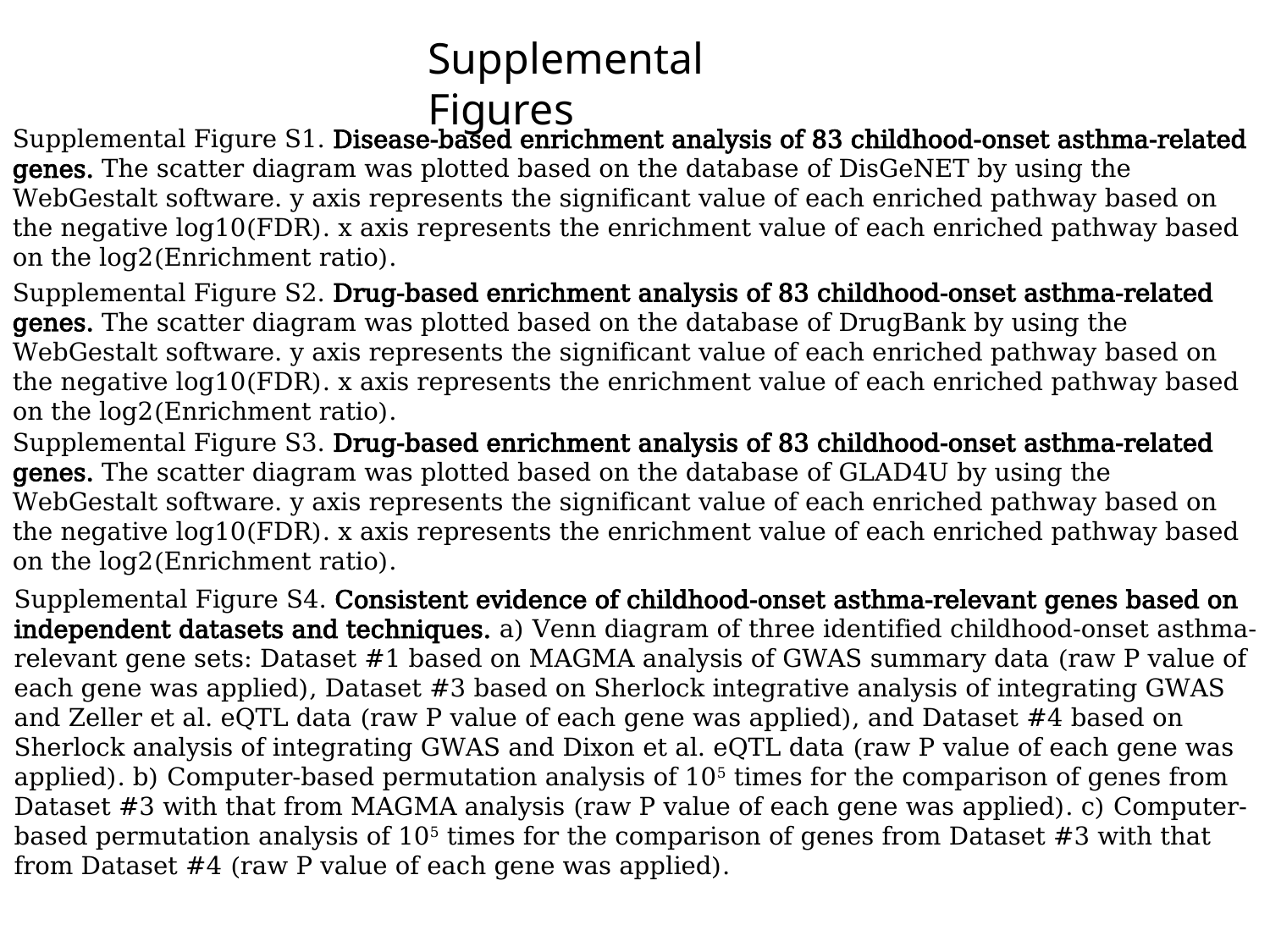

Supplemental Figures
Supplemental Figure S1. Disease-based enrichment analysis of 83 childhood-onset asthma-related genes. The scatter diagram was plotted based on the database of DisGeNET by using the WebGestalt software. y axis represents the significant value of each enriched pathway based on the negative log10(FDR). x axis represents the enrichment value of each enriched pathway based on the log2(Enrichment ratio).
Supplemental Figure S2. Drug-based enrichment analysis of 83 childhood-onset asthma-related genes. The scatter diagram was plotted based on the database of DrugBank by using the WebGestalt software. y axis represents the significant value of each enriched pathway based on the negative log10(FDR). x axis represents the enrichment value of each enriched pathway based on the log2(Enrichment ratio).
Supplemental Figure S3. Drug-based enrichment analysis of 83 childhood-onset asthma-related genes. The scatter diagram was plotted based on the database of GLAD4U by using the WebGestalt software. y axis represents the significant value of each enriched pathway based on the negative log10(FDR). x axis represents the enrichment value of each enriched pathway based on the log2(Enrichment ratio).
Supplemental Figure S4. Consistent evidence of childhood-onset asthma-relevant genes based on independent datasets and techniques. a) Venn diagram of three identified childhood-onset asthma-relevant gene sets: Dataset #1 based on MAGMA analysis of GWAS summary data (raw P value of each gene was applied), Dataset #3 based on Sherlock integrative analysis of integrating GWAS and Zeller et al. eQTL data (raw P value of each gene was applied), and Dataset #4 based on Sherlock analysis of integrating GWAS and Dixon et al. eQTL data (raw P value of each gene was applied). b) Computer-based permutation analysis of 105 times for the comparison of genes from Dataset #3 with that from MAGMA analysis (raw P value of each gene was applied). c) Computer-based permutation analysis of 105 times for the comparison of genes from Dataset #3 with that from Dataset #4 (raw P value of each gene was applied).

## Slide 2
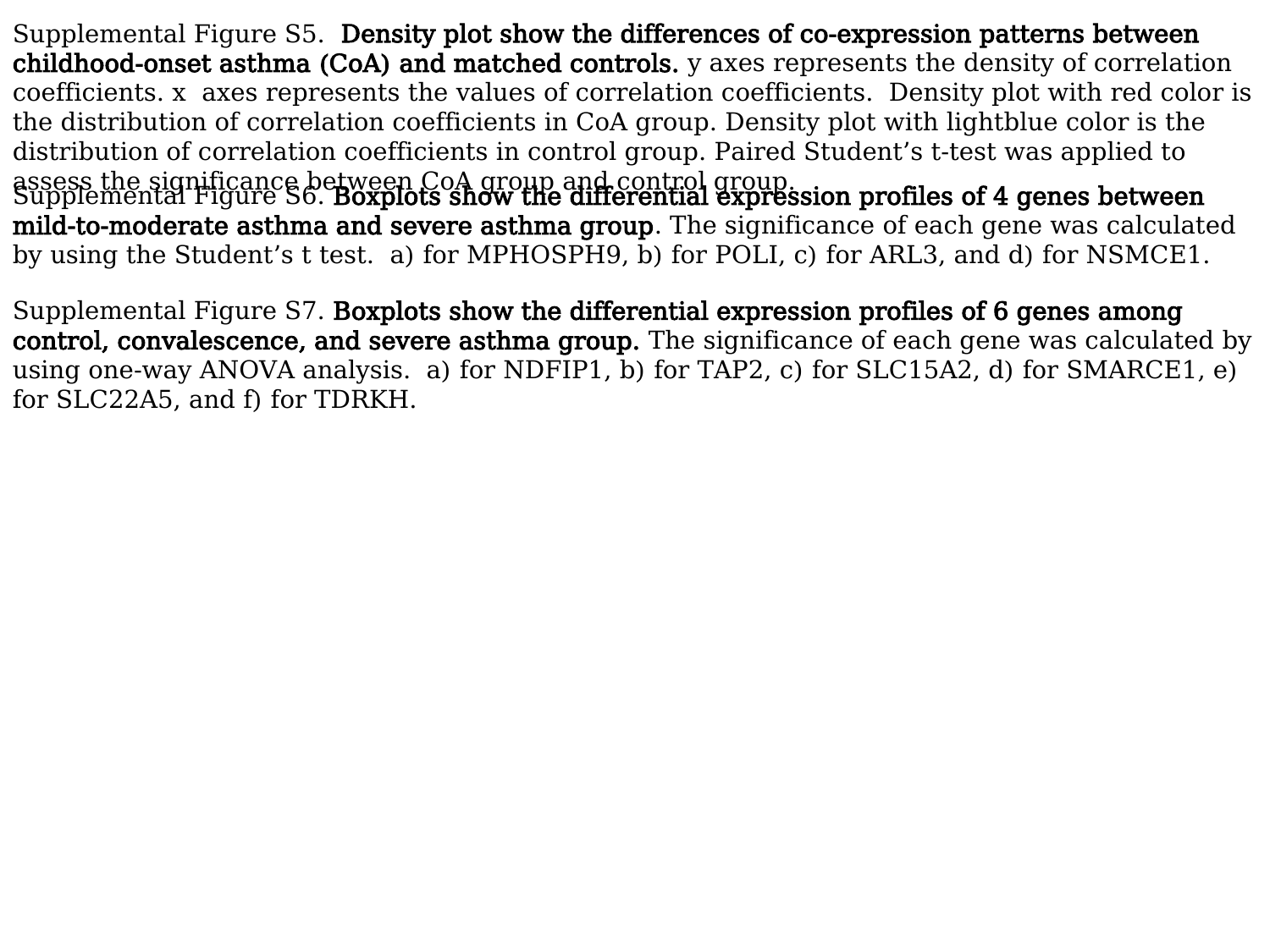

Supplemental Figure S5. Density plot show the differences of co-expression patterns between childhood-onset asthma (CoA) and matched controls. y axes represents the density of correlation coefficients. x axes represents the values of correlation coefficients. Density plot with red color is the distribution of correlation coefficients in CoA group. Density plot with lightblue color is the distribution of correlation coefficients in control group. Paired Student’s t-test was applied to assess the significance between CoA group and control group.
Supplemental Figure S6. Boxplots show the differential expression profiles of 4 genes between mild-to-moderate asthma and severe asthma group. The significance of each gene was calculated by using the Student’s t test. a) for MPHOSPH9, b) for POLI, c) for ARL3, and d) for NSMCE1.
Supplemental Figure S7. Boxplots show the differential expression profiles of 6 genes among control, convalescence, and severe asthma group. The significance of each gene was calculated by using one-way ANOVA analysis. a) for NDFIP1, b) for TAP2, c) for SLC15A2, d) for SMARCE1, e) for SLC22A5, and f) for TDRKH.

## Slide 3
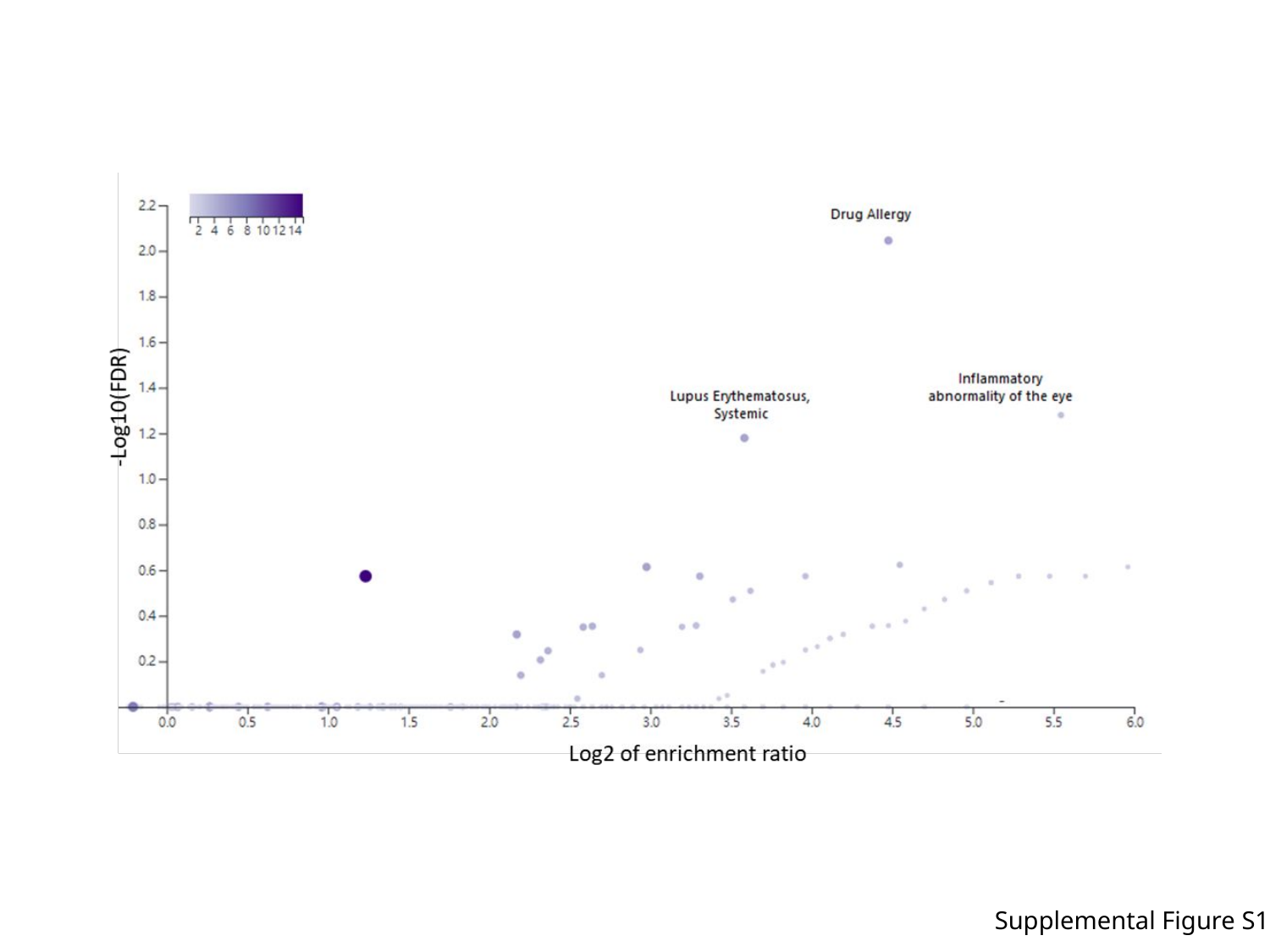

Supplemental Figure S1

## Slide 4
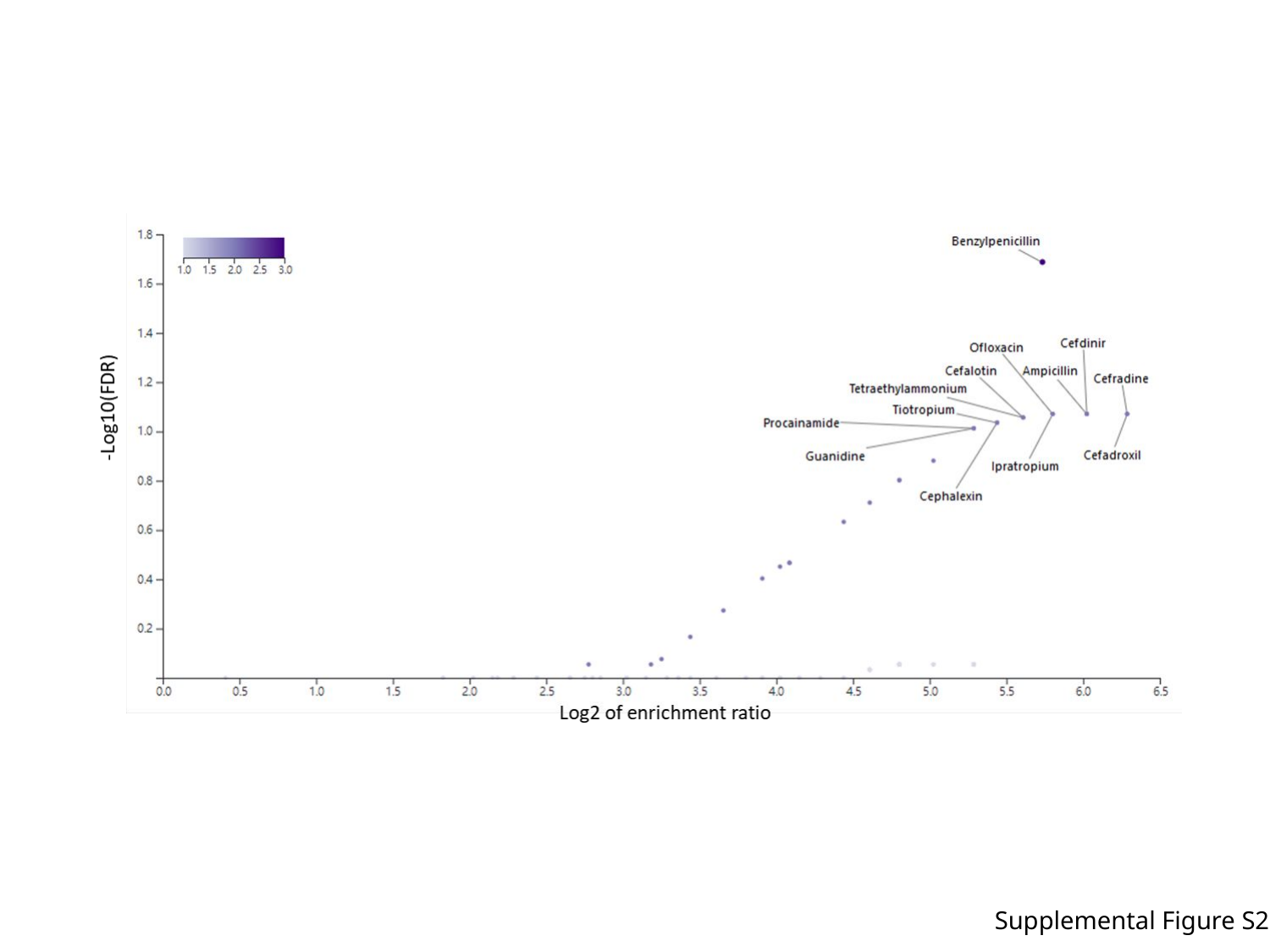

Supplemental Figure S2

## Slide 5
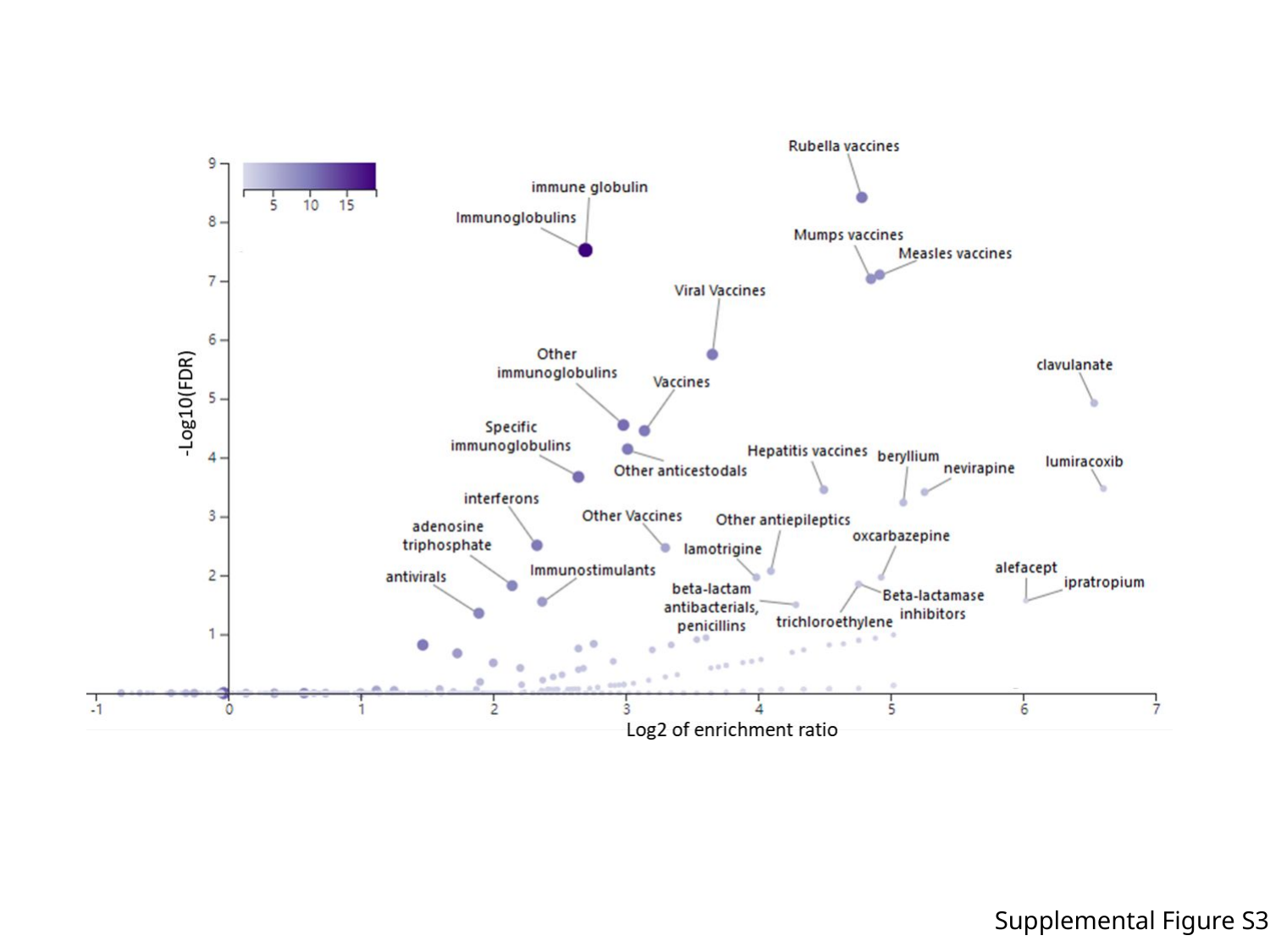

Supplemental Figure S3

## Slide 6
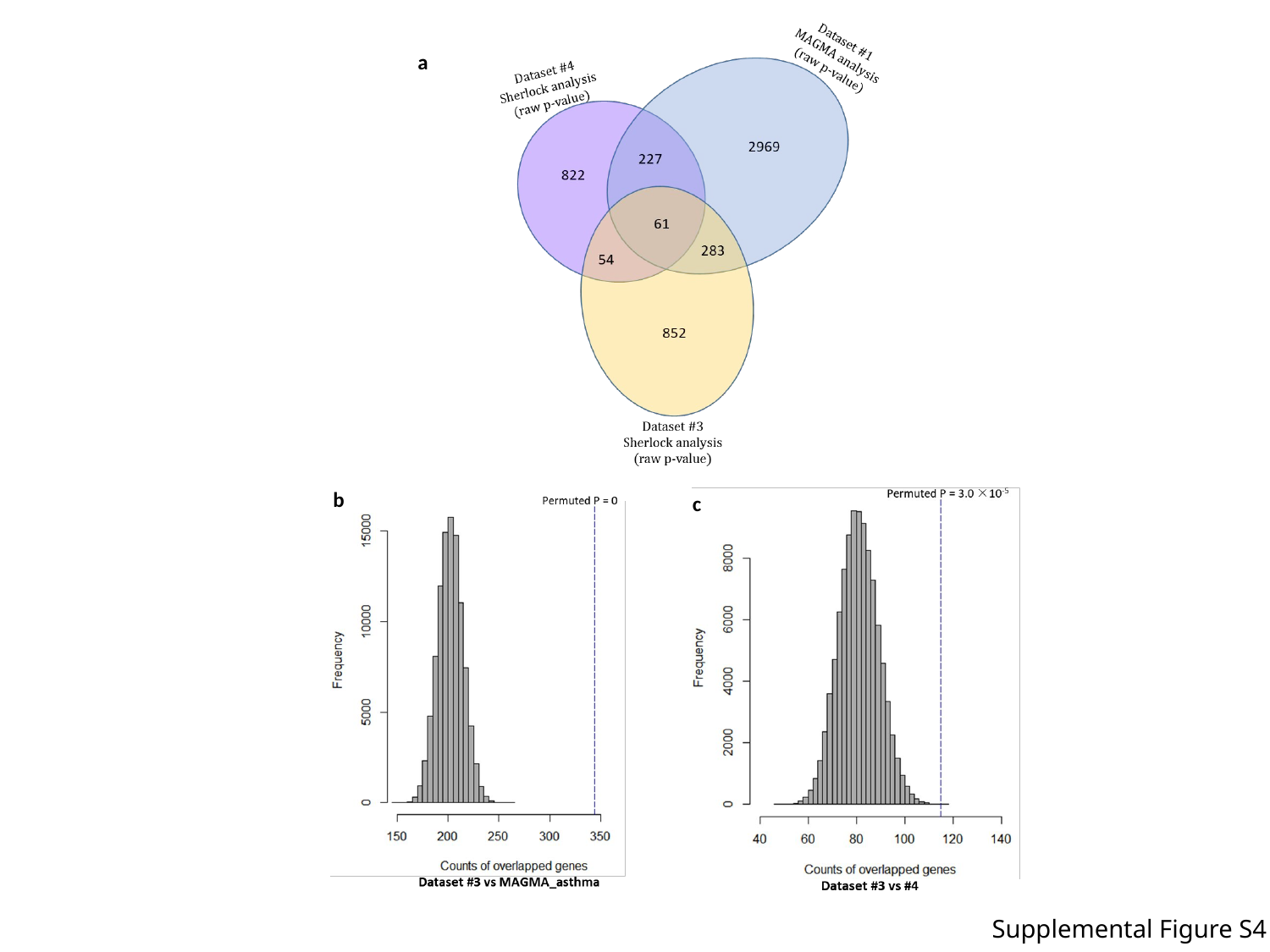

a
b
c
Supplemental Figure S4

## Slide 7
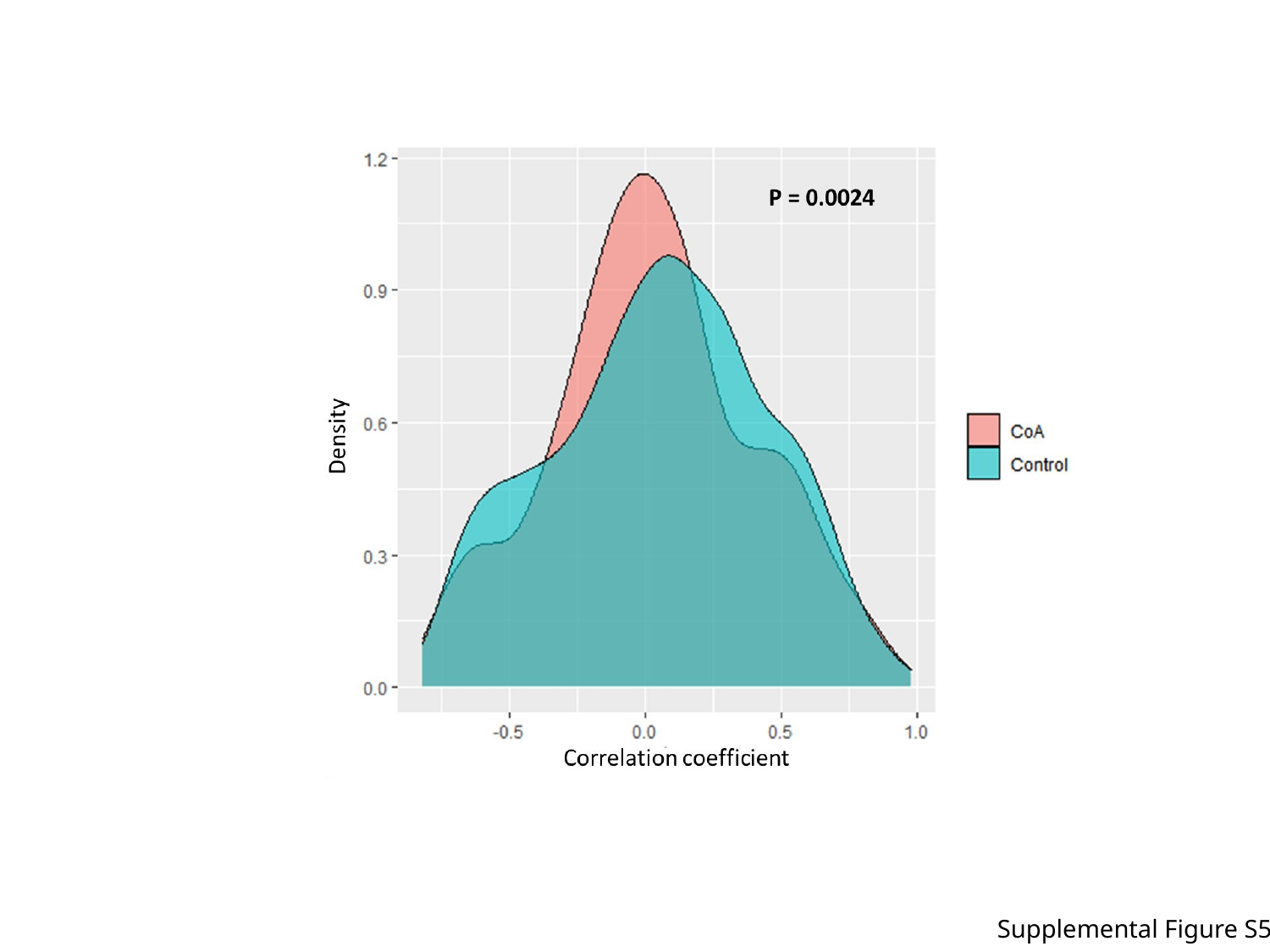

Supplemental Figure S5

## Slide 8
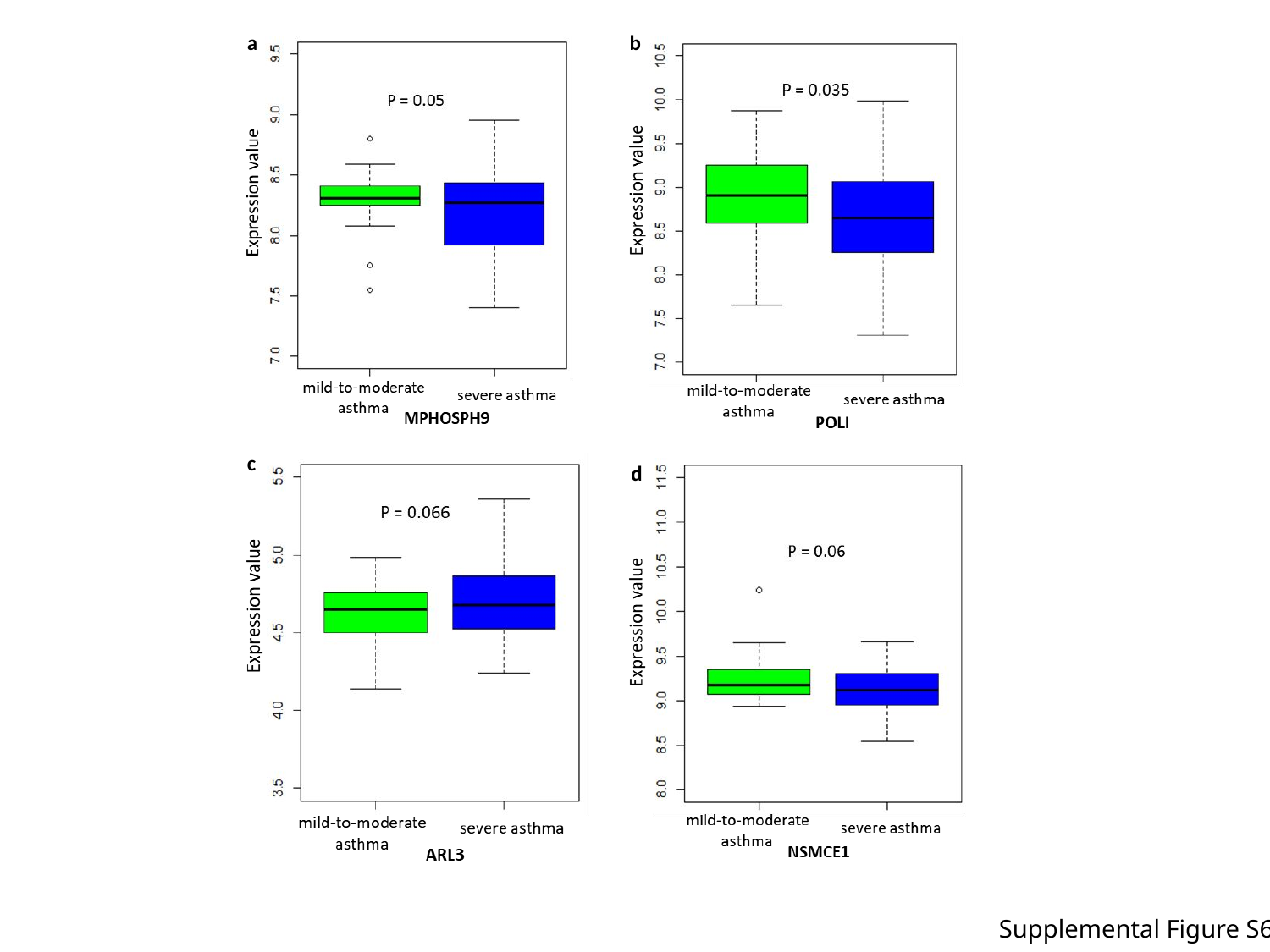

a
b
c
d
Supplemental Figure S6

## Slide 9
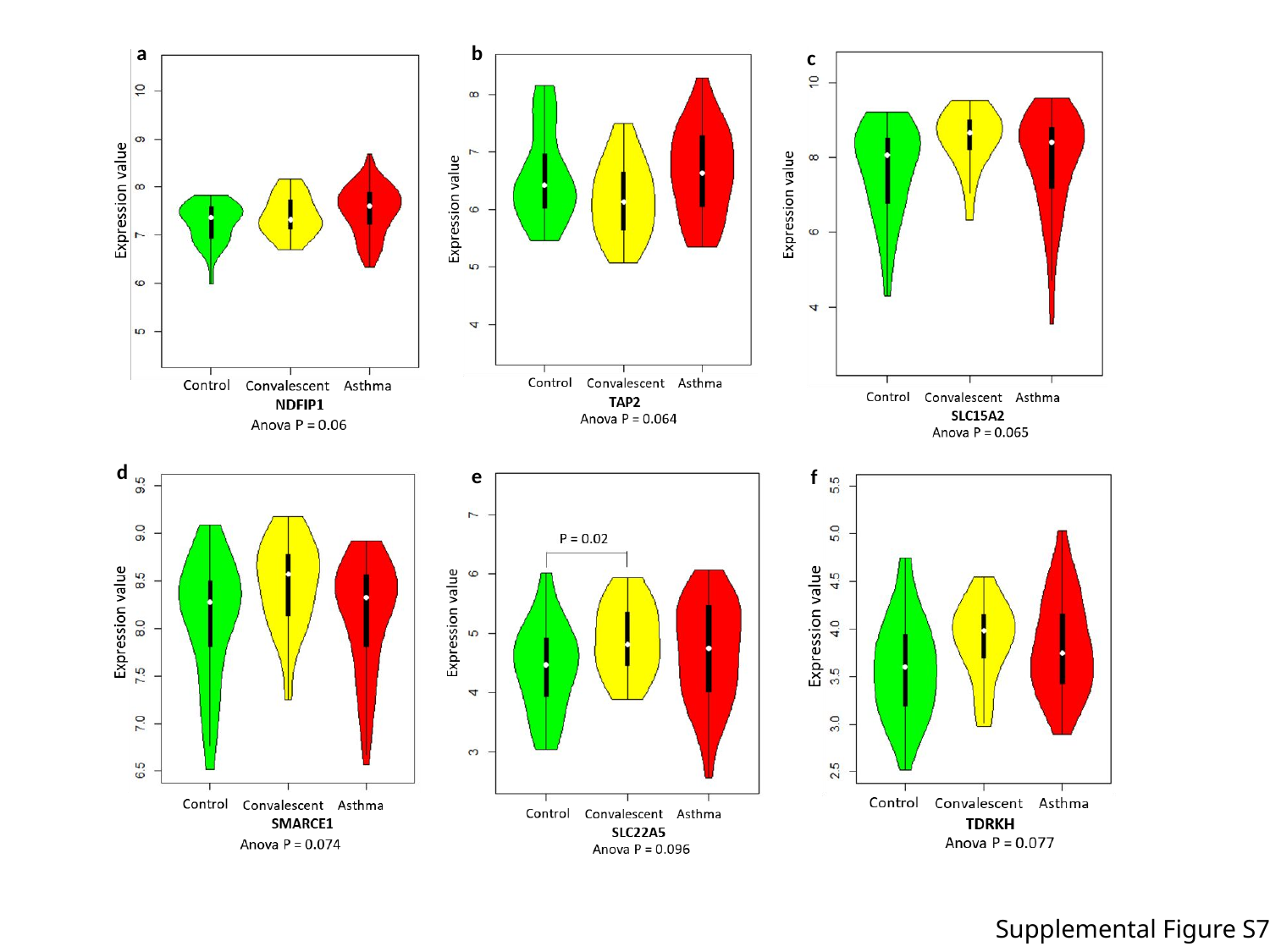

a
b
c
d
e
f
Supplemental Figure S7
